# Supplementary material for: Body shape and performance on the US Army Combat Fitness Test: Insights from a 3D body image scanner
Source: PLoS One. 2023 May 3;18(5):e0283566. doi: 10.1371/journal.pone.0283566 (PMC10155989; doi:10.1371/journal.pone.0283566)
Supplement: S1 File — (DOCX) [file pone.0283566.s001.docx]

To perform the MDL, soldiers use a hexagonal or “trap” bar and lift the maximum possible weight for three consecutive repetitions, lightly tapping the ground before repeating. The minimum passing score for this event is 140 lb (63.5 kg) and the maximum achievable score for record is 340 lb (154.2 kg)^1^.

The SPT requires soldiers to throw a 10 lb (4.5 kg) medicine ball overhead behind them as far as possible. A minimum passing score is achieved if the ball is thrown a distance of 4.5 m and the maximum score is achieved at a distance of 12.5 m.

The HRPU requires completing as many hand-release pushups as possible in two minutes. Each repetition of this event consists of three movements. The soldier begins in the prone position with her hands flat on the ground and directly beneath the shoulders. For the first movement, the soldier pushes the whole body up as a single unit by fully extending the elbows. For the second movement, the soldier bends her elbows to lower the body back to the ground. For the third movement, the soldier immediately moves both arms out to the side, straightening the elbows so that the entire body is in a “T” position. The elbows then bend to move the hands back under the shoulder. This completes one repetition. The minimum passing score for this event is 10 repetitions and the maximum achievable score for record is 60 repetitions^1^.

The SDC event requires soldiers to conduct five 50 m shuttles for time – sprint, drag, lateral, carry, and sprint. The soldier begins this event in the prone position, with his head behind the start line. On the command “go,” the soldier stands and sprints 25 m, touching the 25 m line with his hand and foot, and sprints back to the start line. The soldier then grasps a strap handle with a 90 lb sled attached and pulls it backwards until the sled crosses the 25 m line. The soldier then turns the sled around and pulls it back until the entire sled crosses the start line. After the sled crosses the start line, the soldier then performs a lateral shuffle to the 25 m line, touching the line with his foot and hand, and performs the lateral back to the start line. The soldier then grasps with each hand a 40 lb kettlebell (total 80 lb) and runs to the 25 m line, steps on or over the line, and returns back to the start line. To finish the event, soldiers then sprint to the 25 m line, touching it with their foot and hand and sprints back crossing the start line. Time is stopped when the soldier crosses the start line after the final sprint. The minimum passing score (or maximum allowable time) for this event is 180 s. The maximum achievable score (or minimum time) for record is 93 s^1^.

The LT event requires soldiers to complete as many leg tucks as possible. The soldier begins this event with a straight-arm hang with an alternating grip and feet off the ground. To begin, the soldier flexes at the elbows and raises her knees until both knees touch both elbows. The soldier returns to the straight-arm position completing one repetition. The minimum passing score for this event is 1 repetition. The maximum achievable score for record is 20 repetitions^1^.

The 2-mile run event requires soldiers to run two miles for time on a generally flat outdoor course. The minimum passing score for this event is 21:00 minutes. The maximum achievable score for record is 13:30 minutes^1^.

REFERENCES

1. Taguchi N. Developing interactional competence in a Japanese study abroad context. Bristol ; Buffalo: Multilingual Matters 2015.
