# Supplementary figures and images for: Body shape and performance on the US Army Combat Fitness Test: Insights from a 3D body image scanner
Source: PLoS One. 2023 May 3;18(5):e0283566. doi: 10.1371/journal.pone.0283566 (PMC10155989; doi:10.1371/journal.pone.0283566)

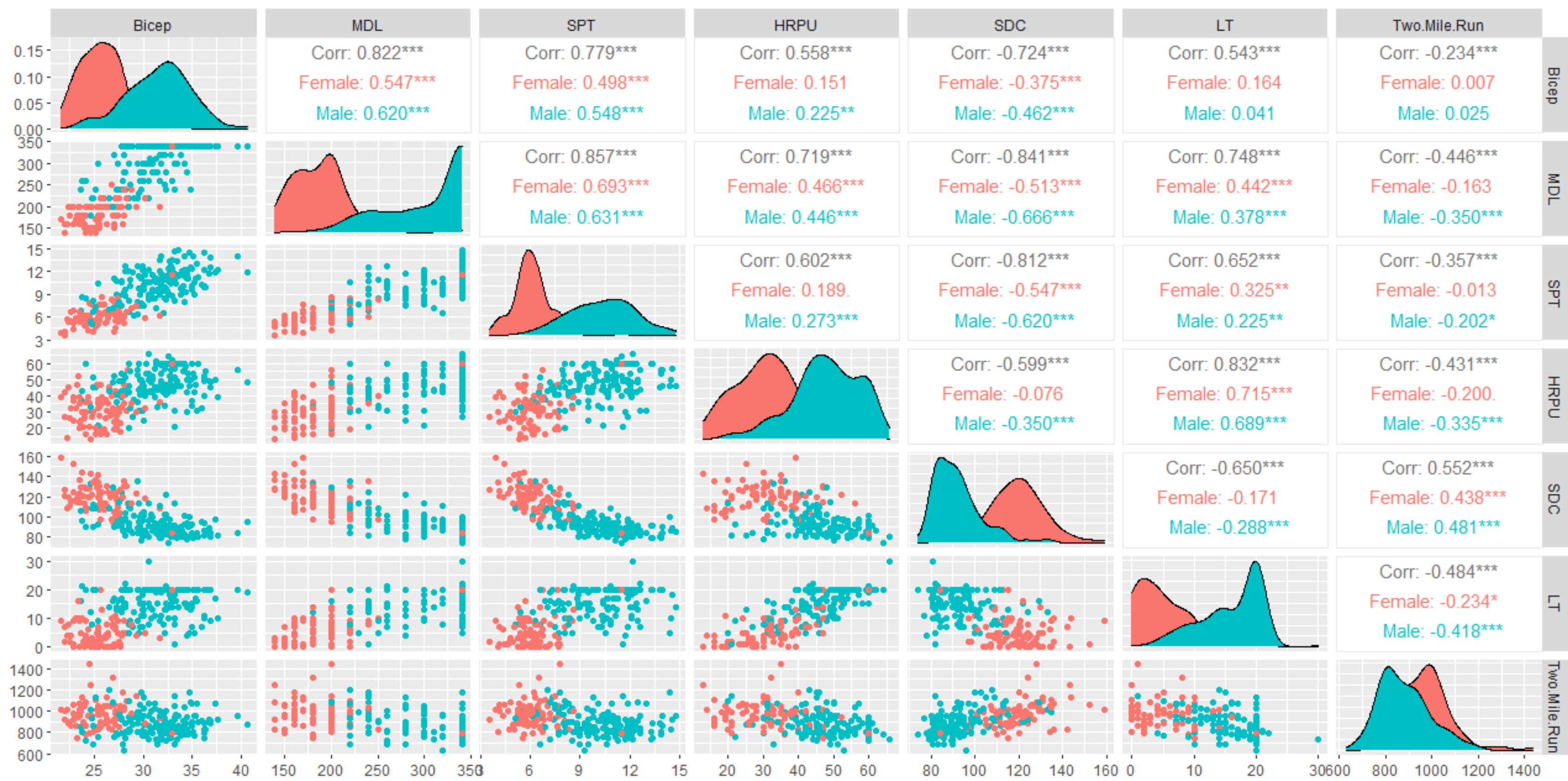

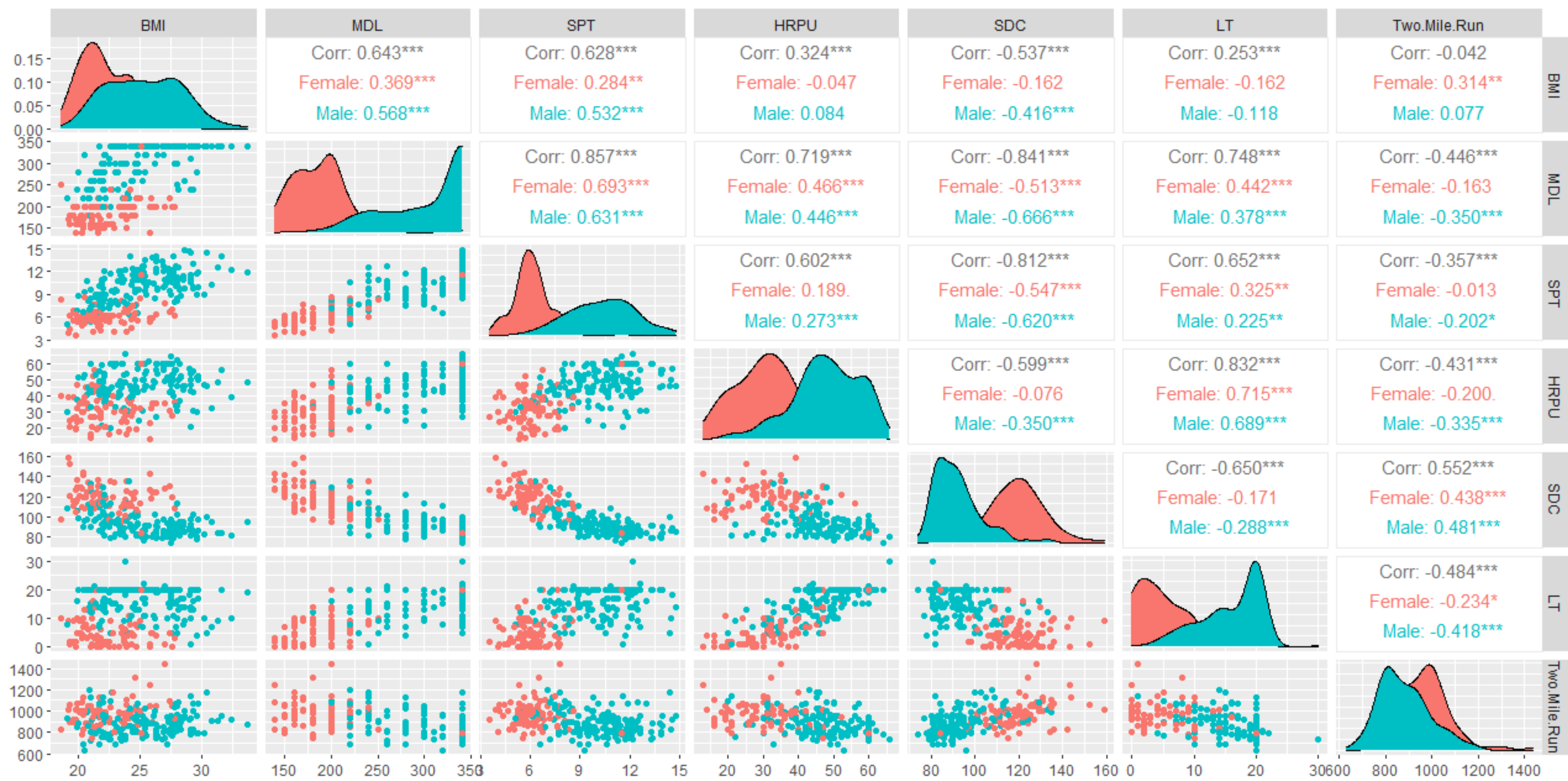

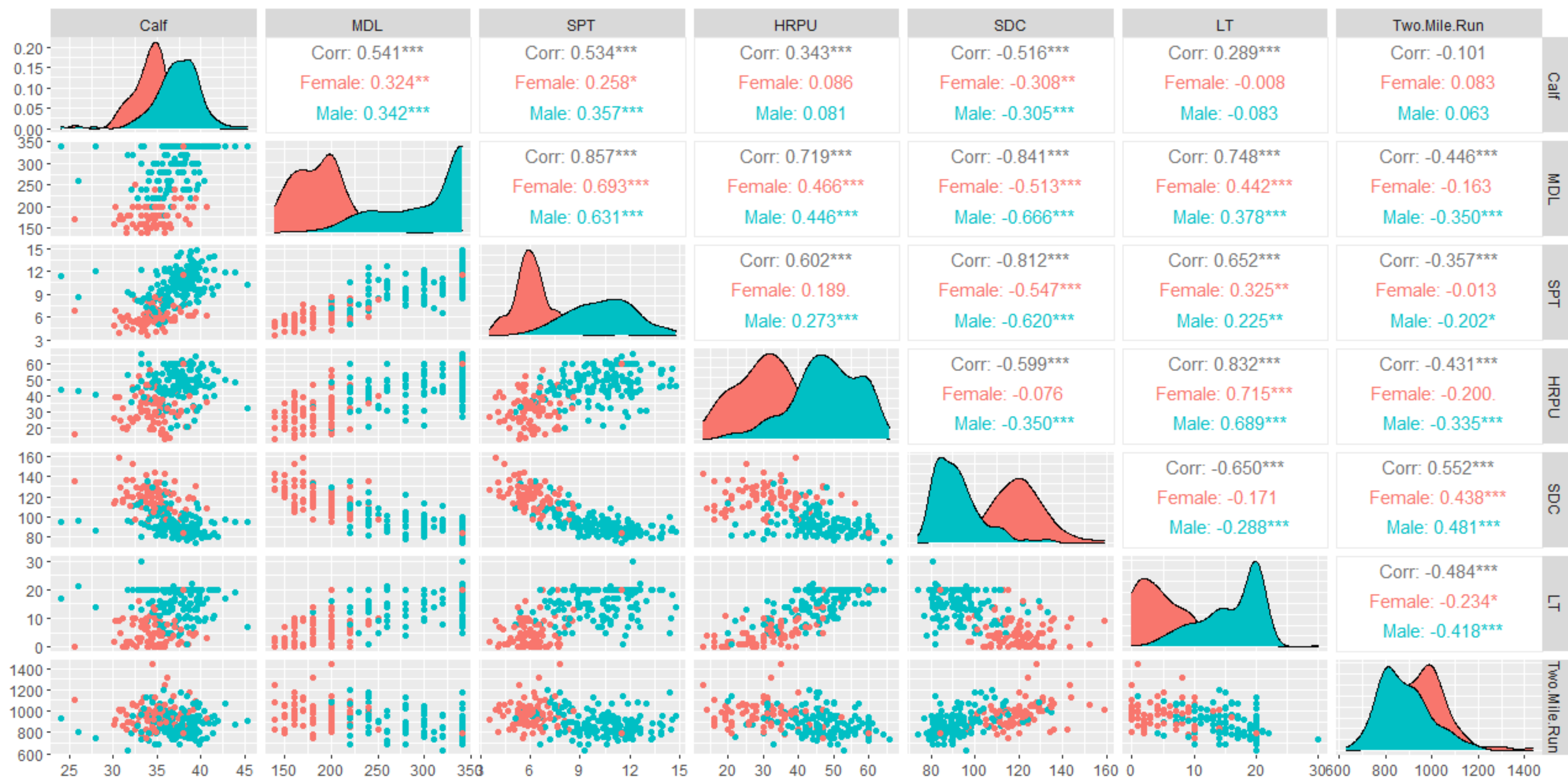

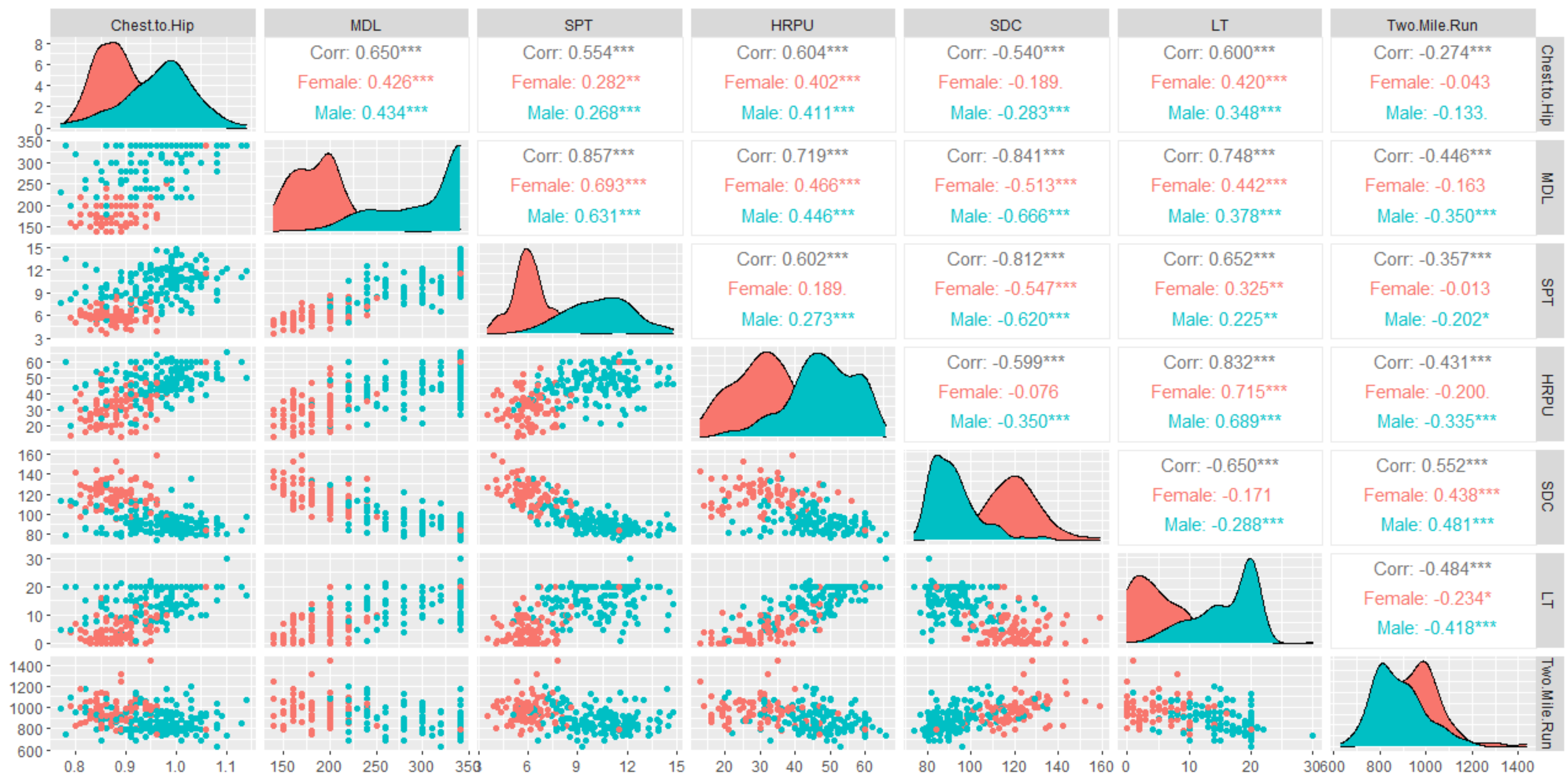

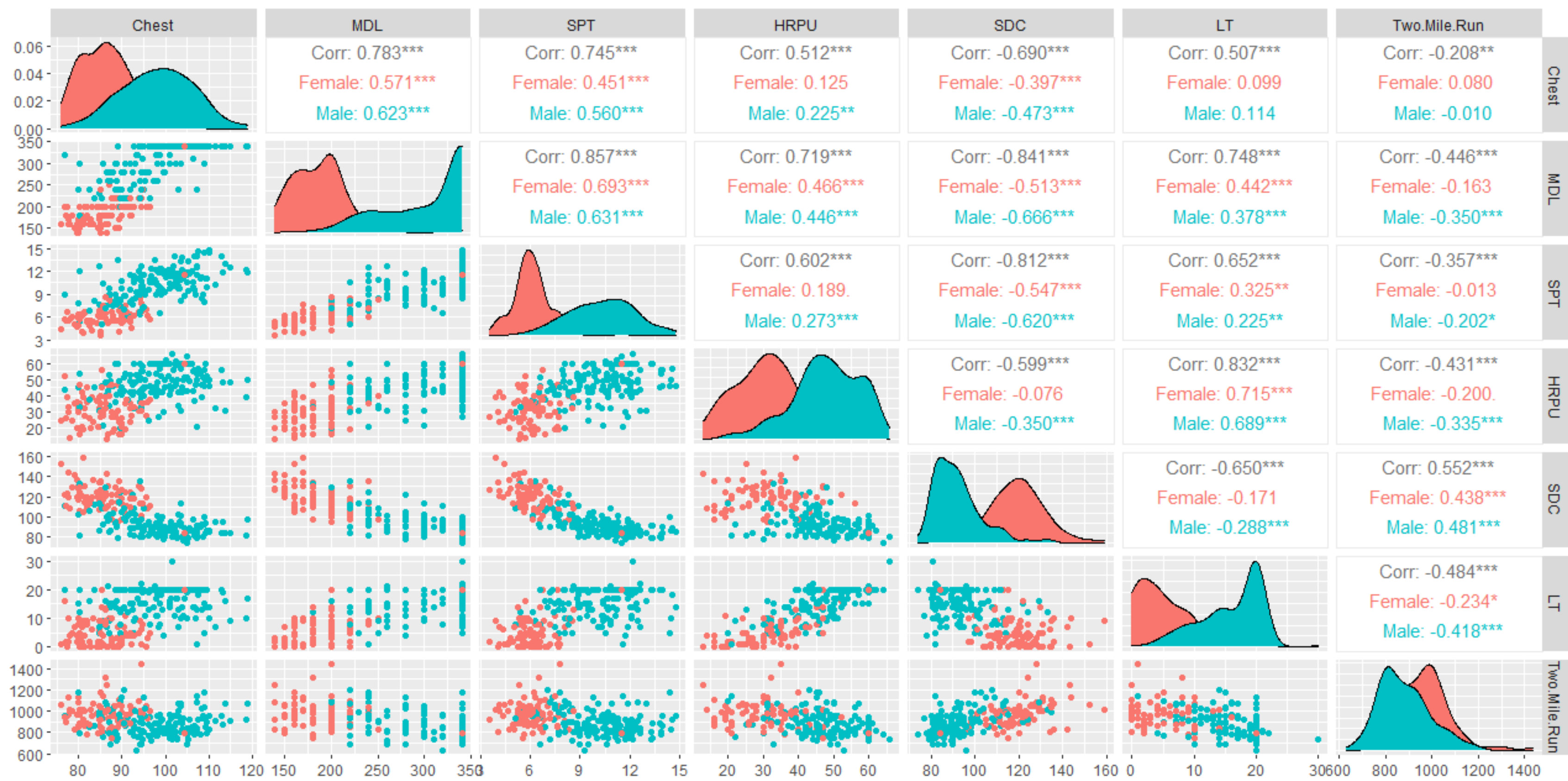

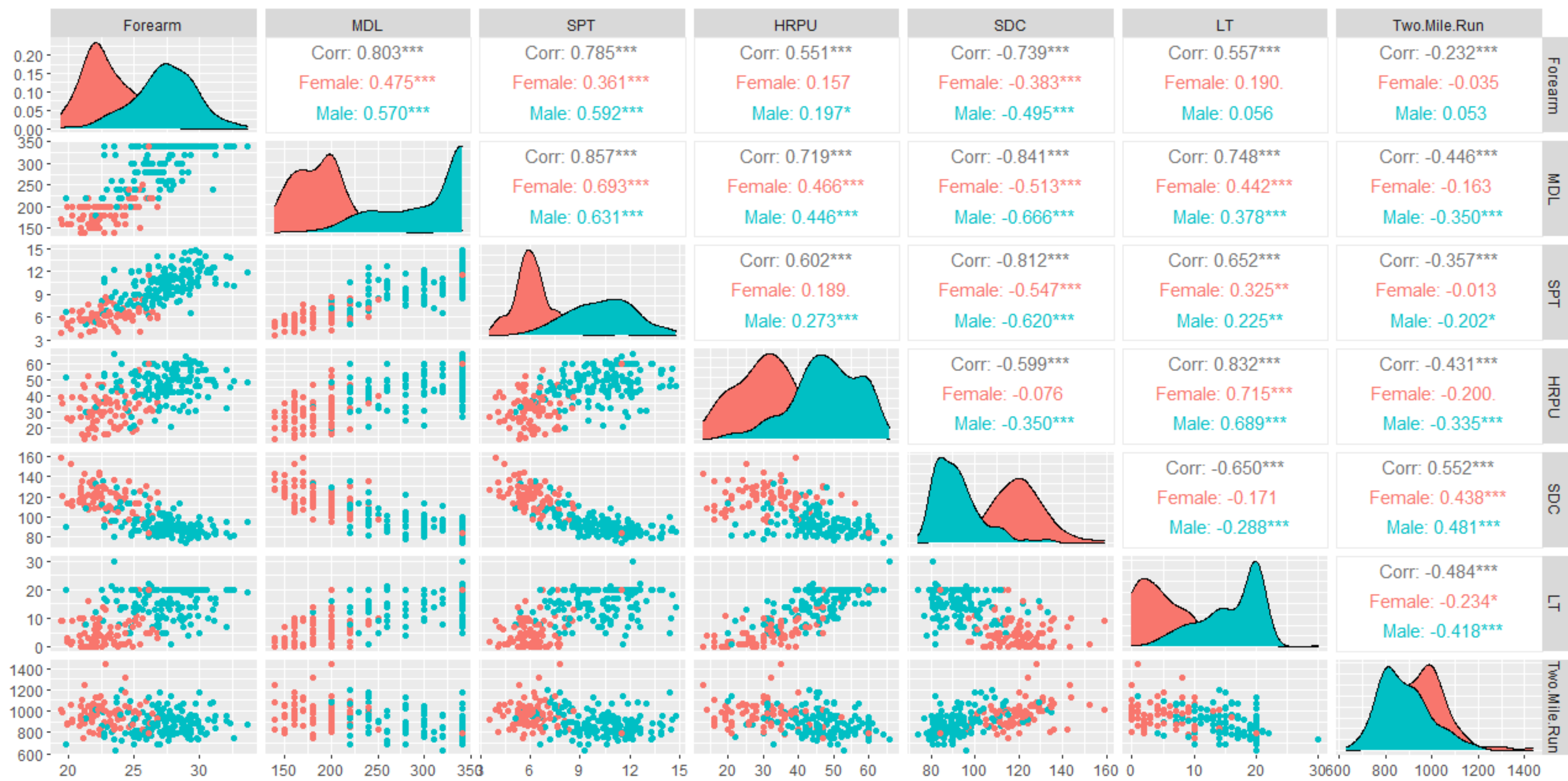

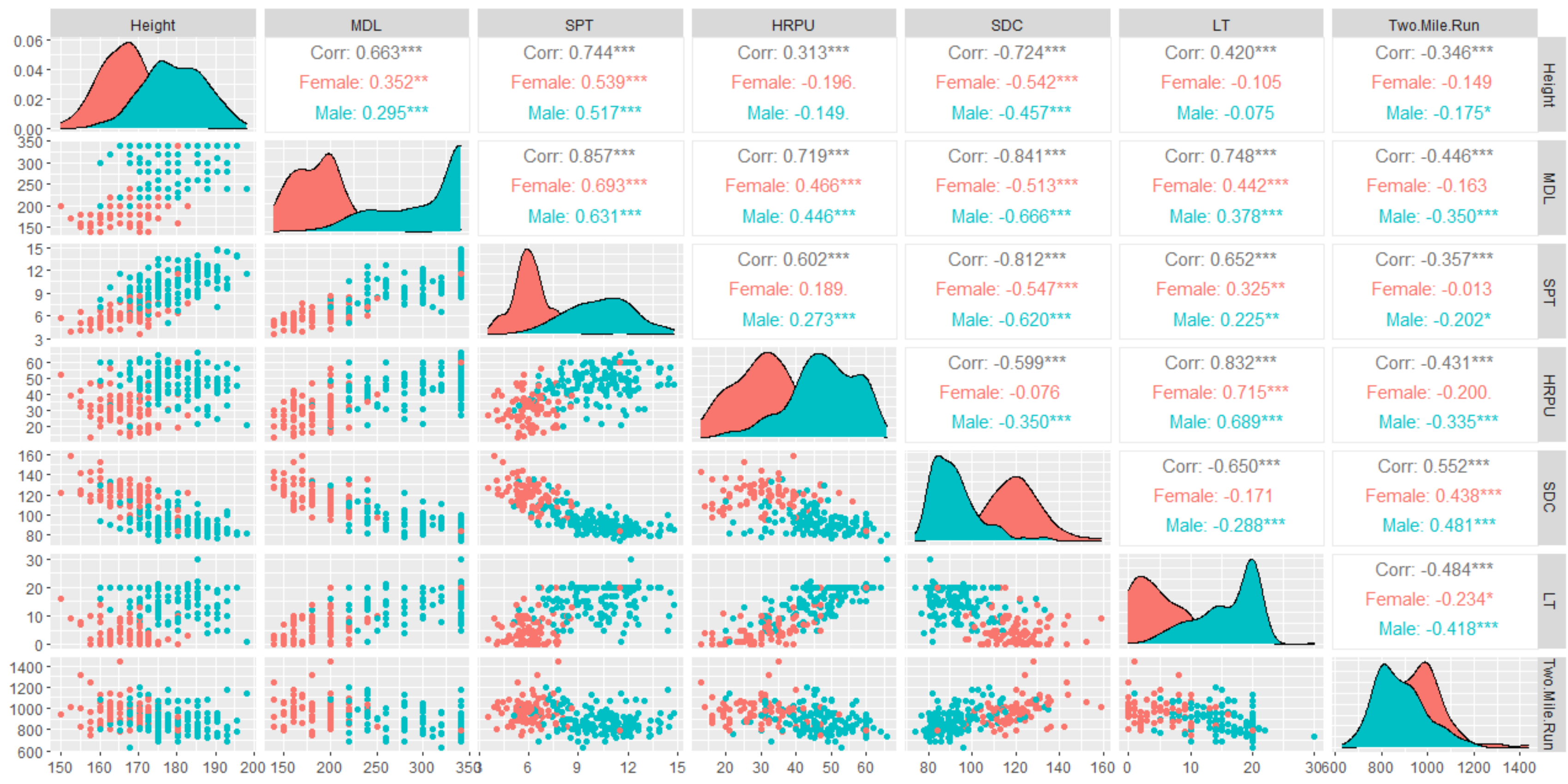

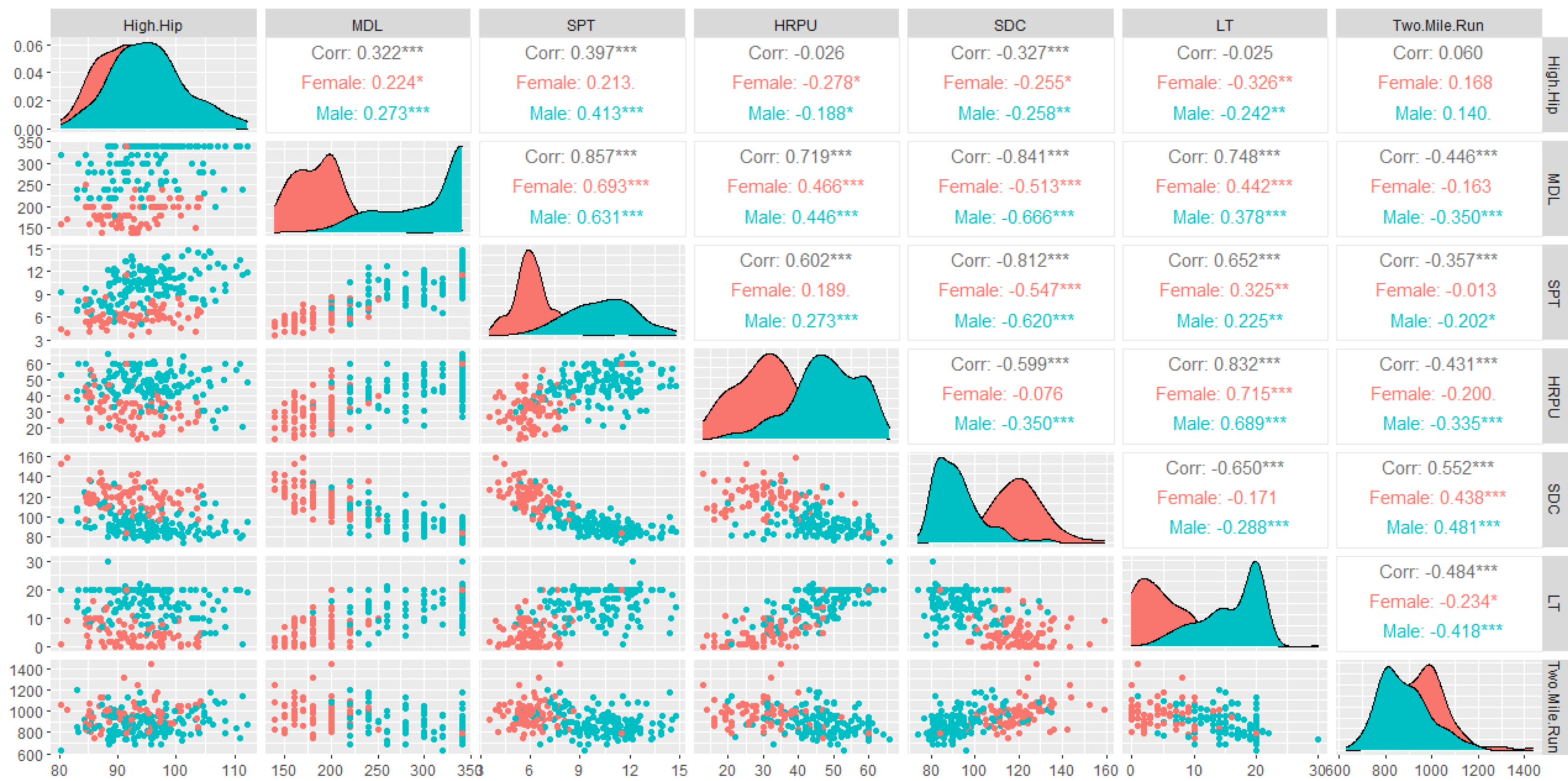

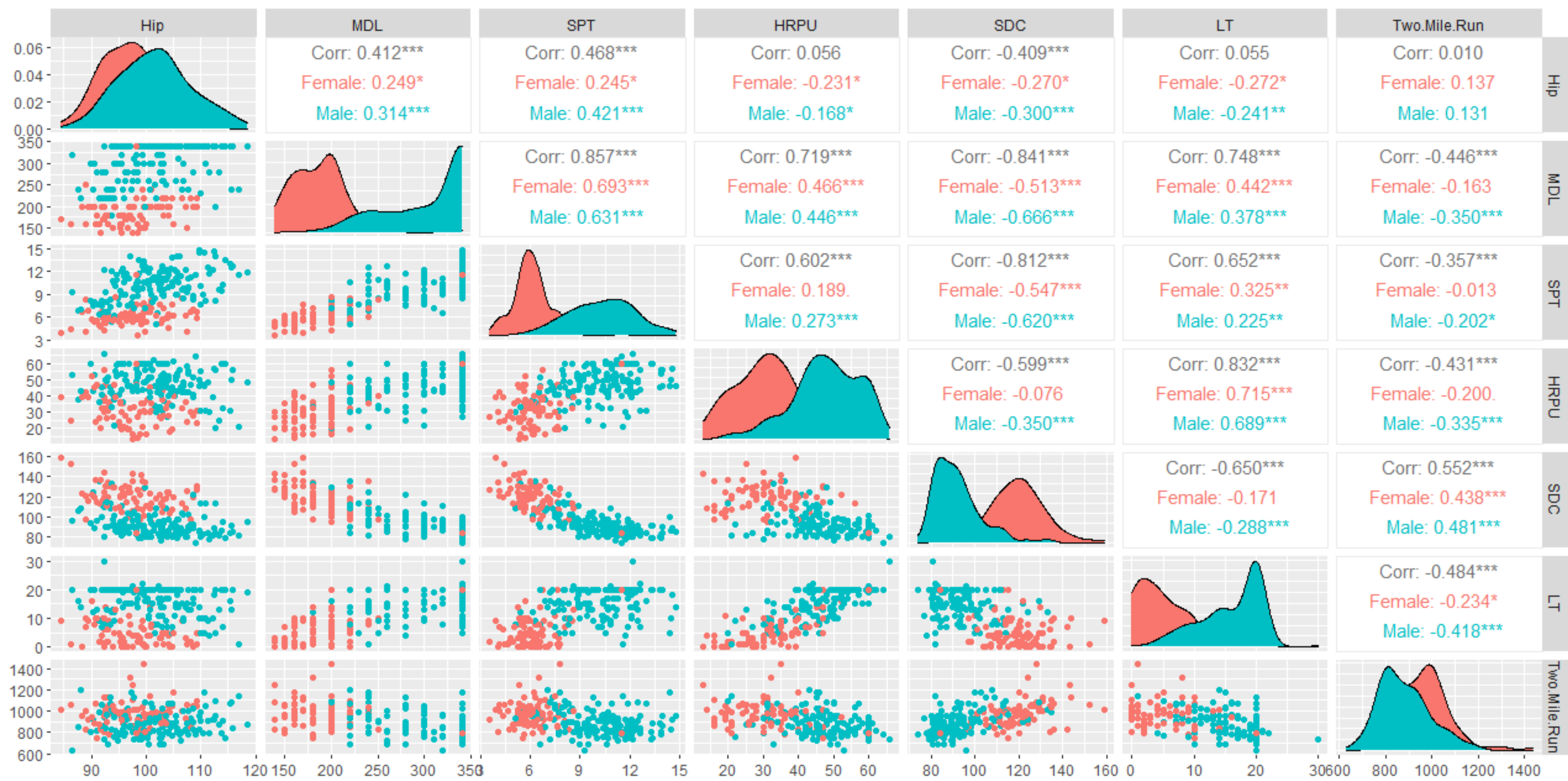

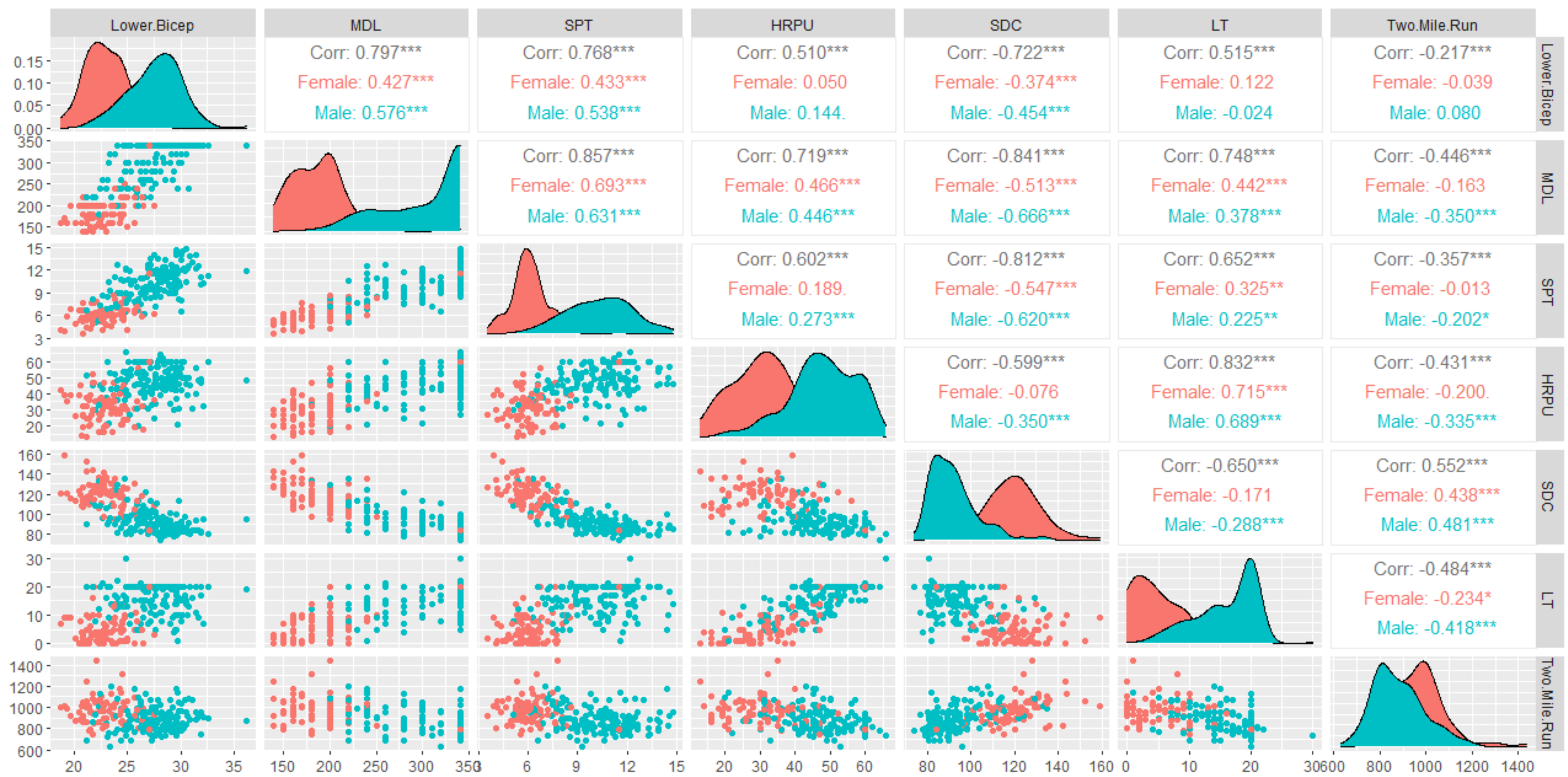

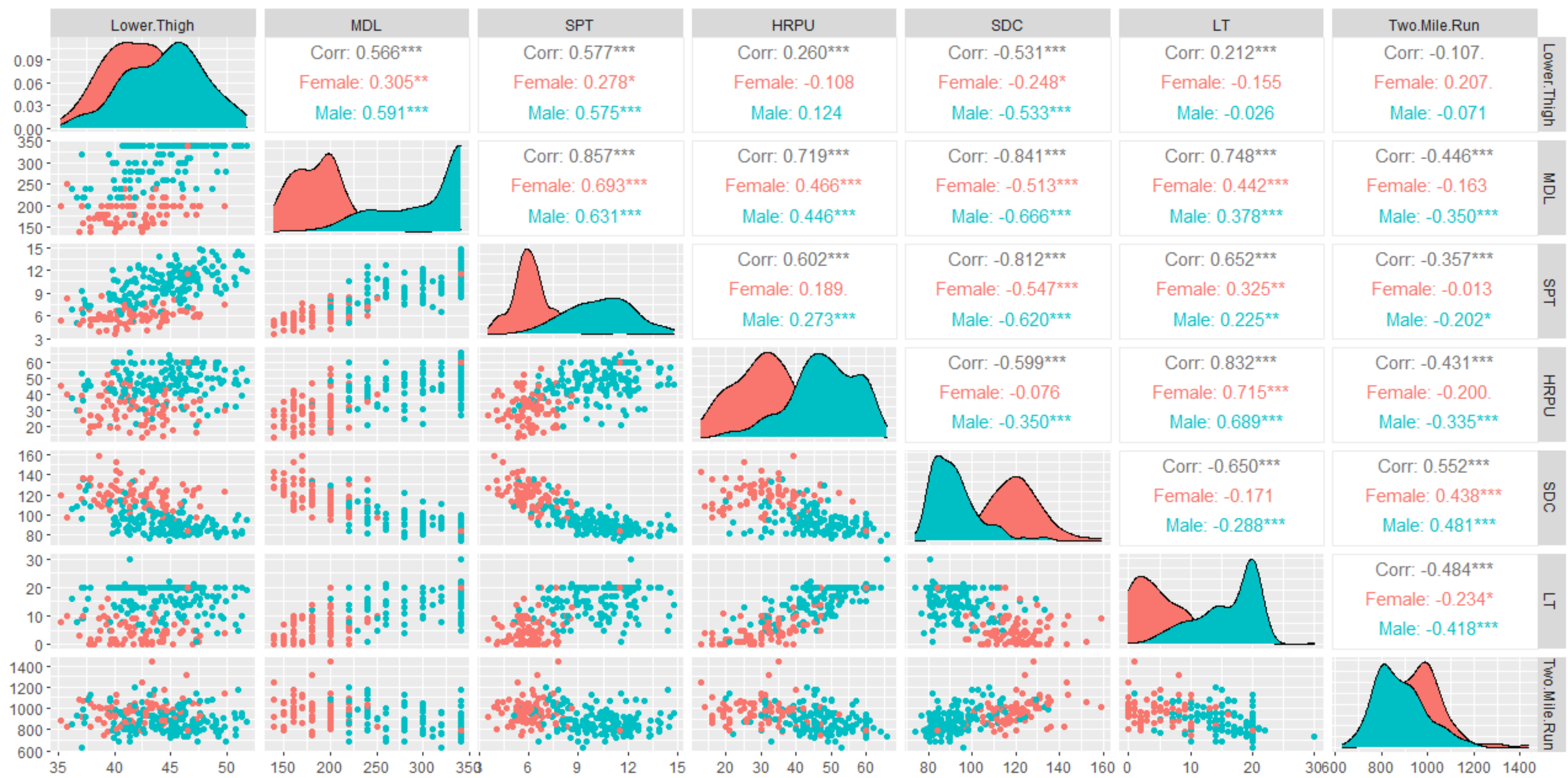

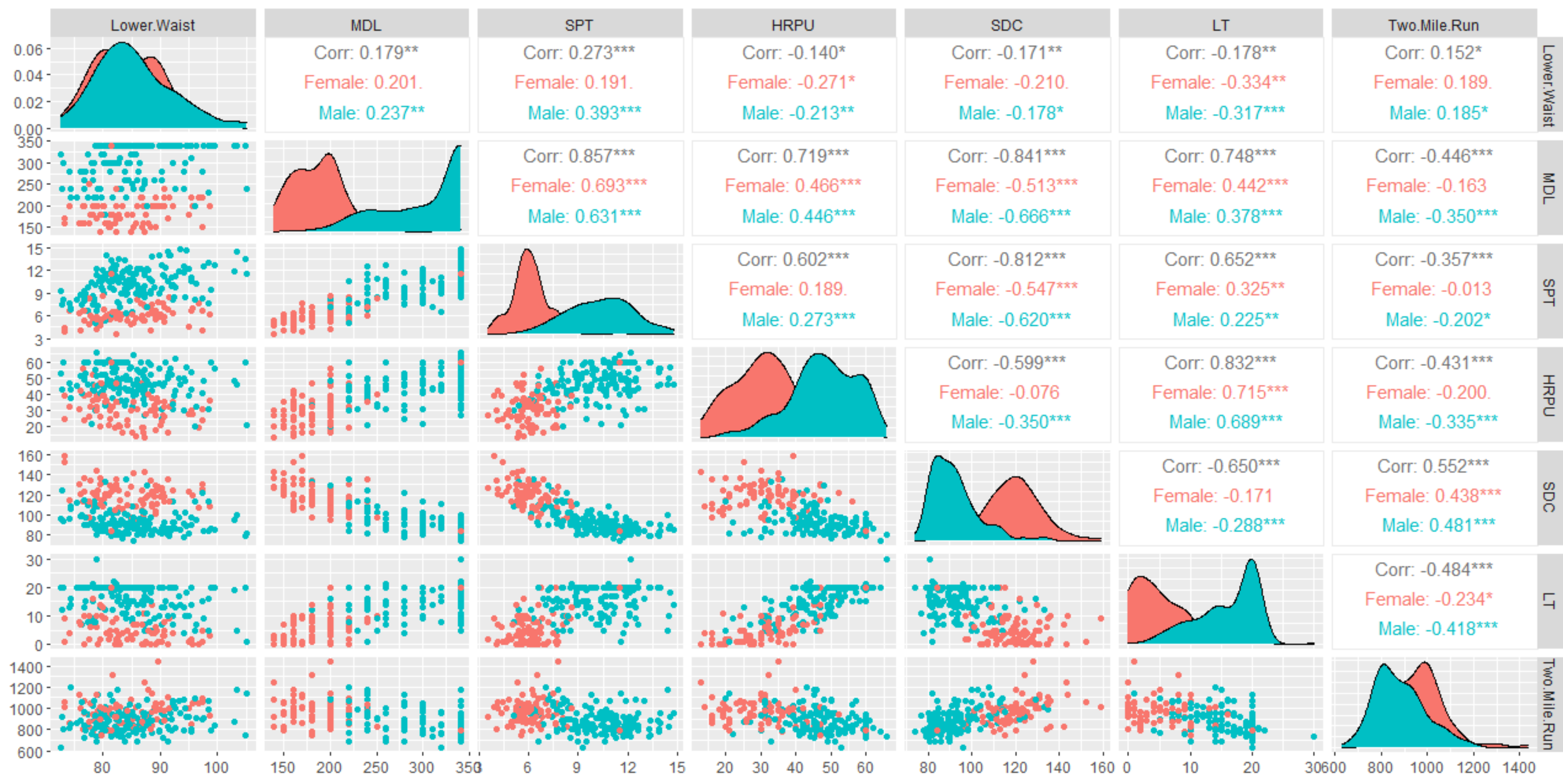

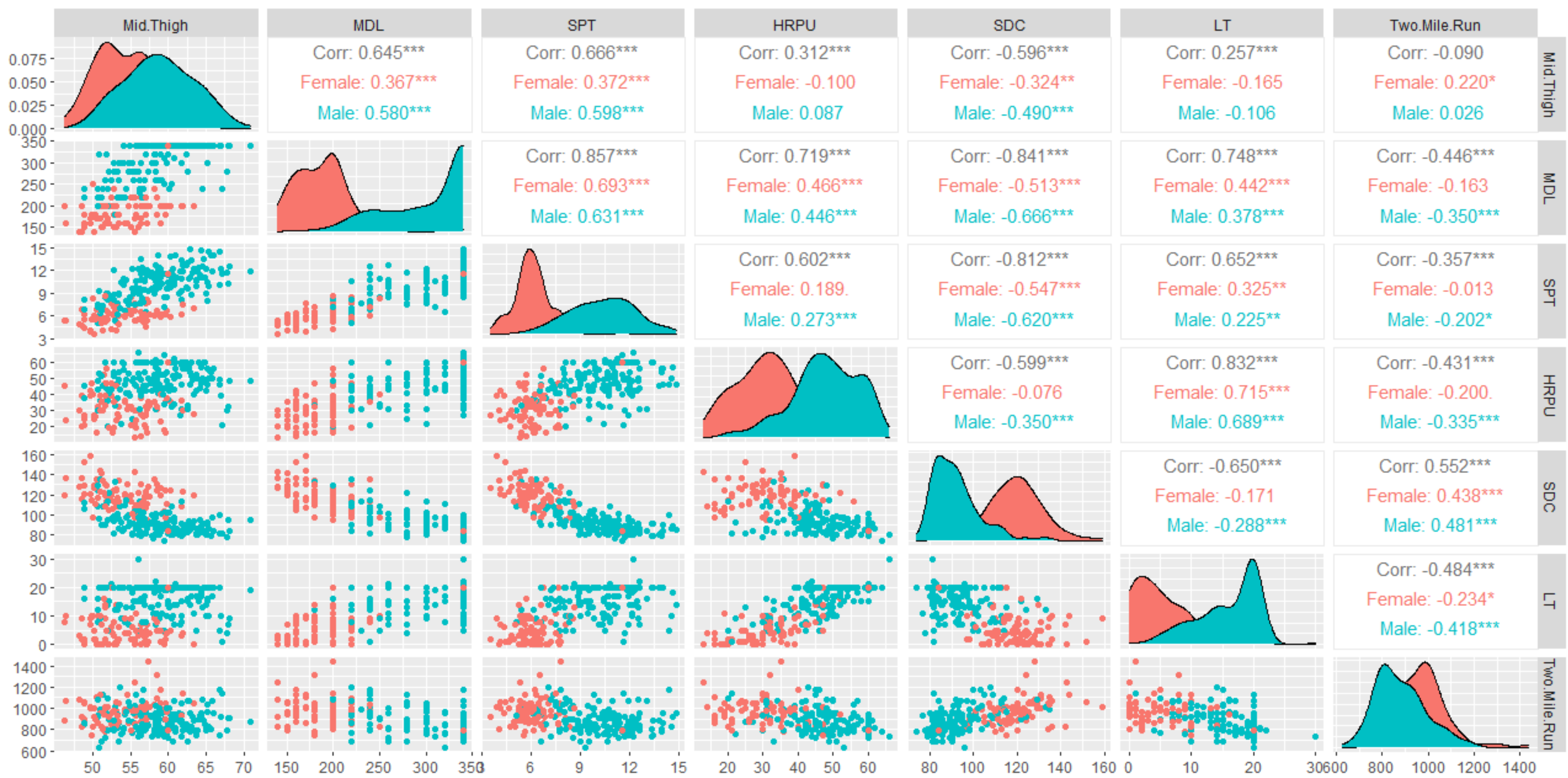

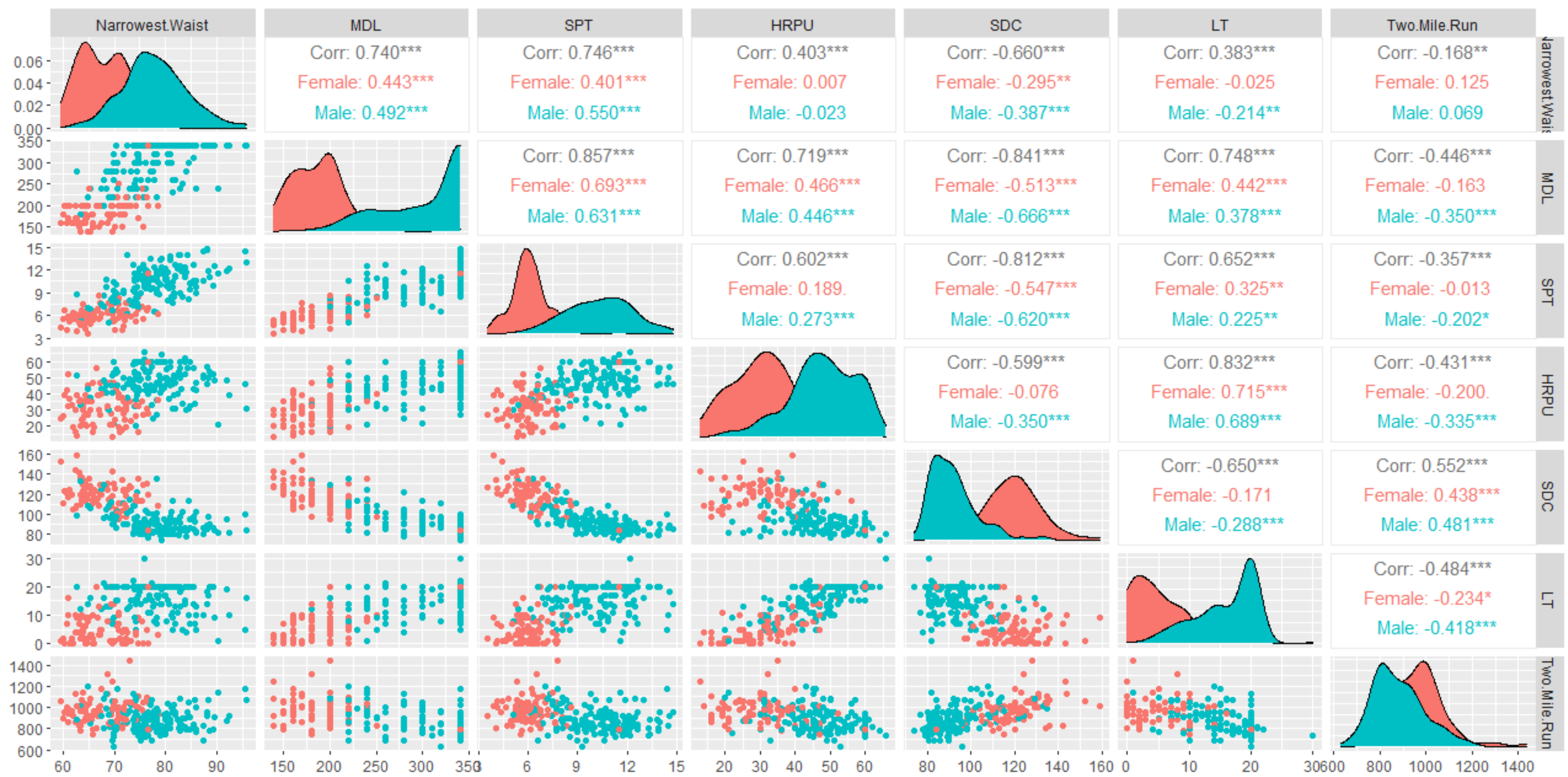

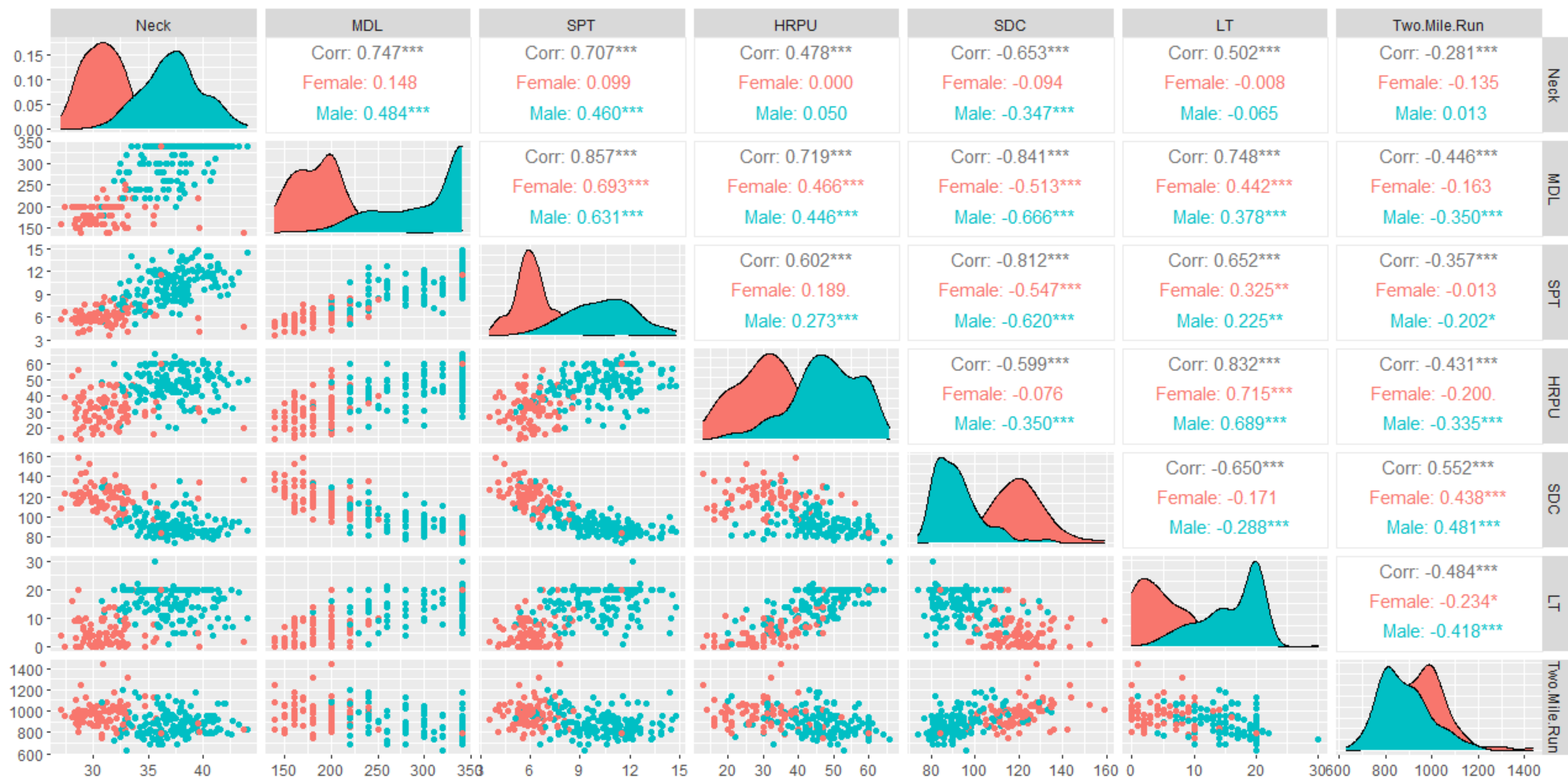

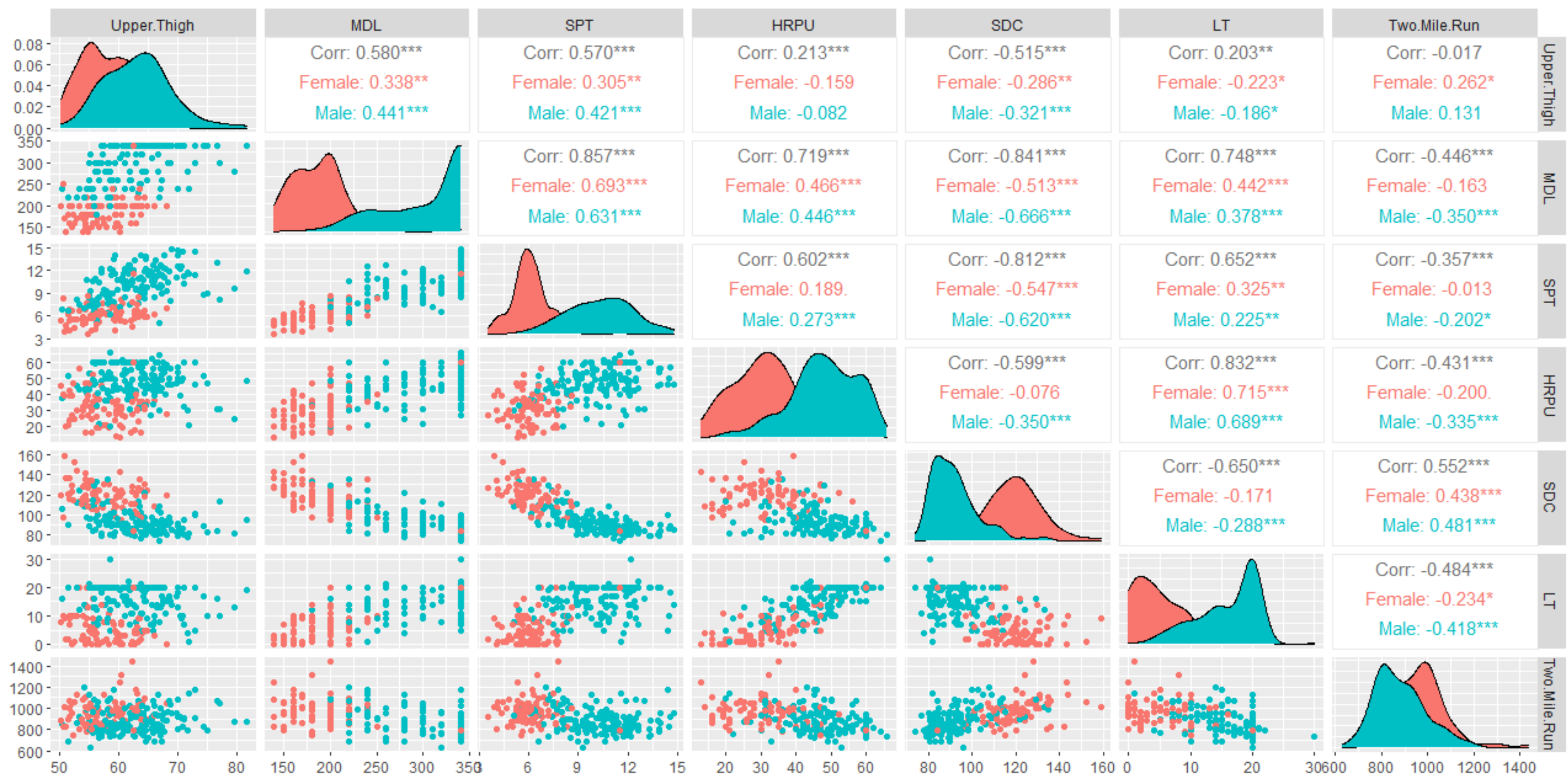

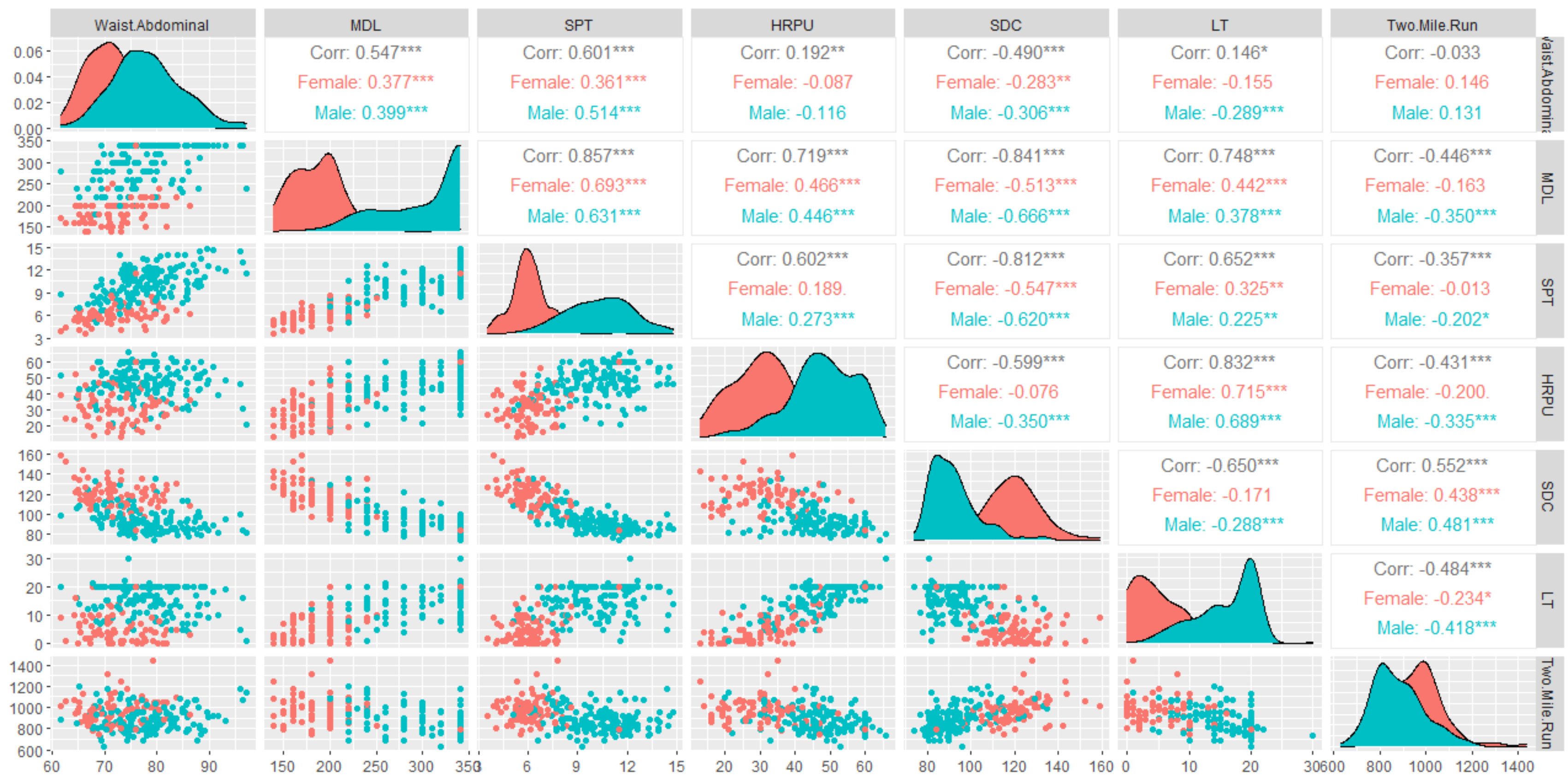

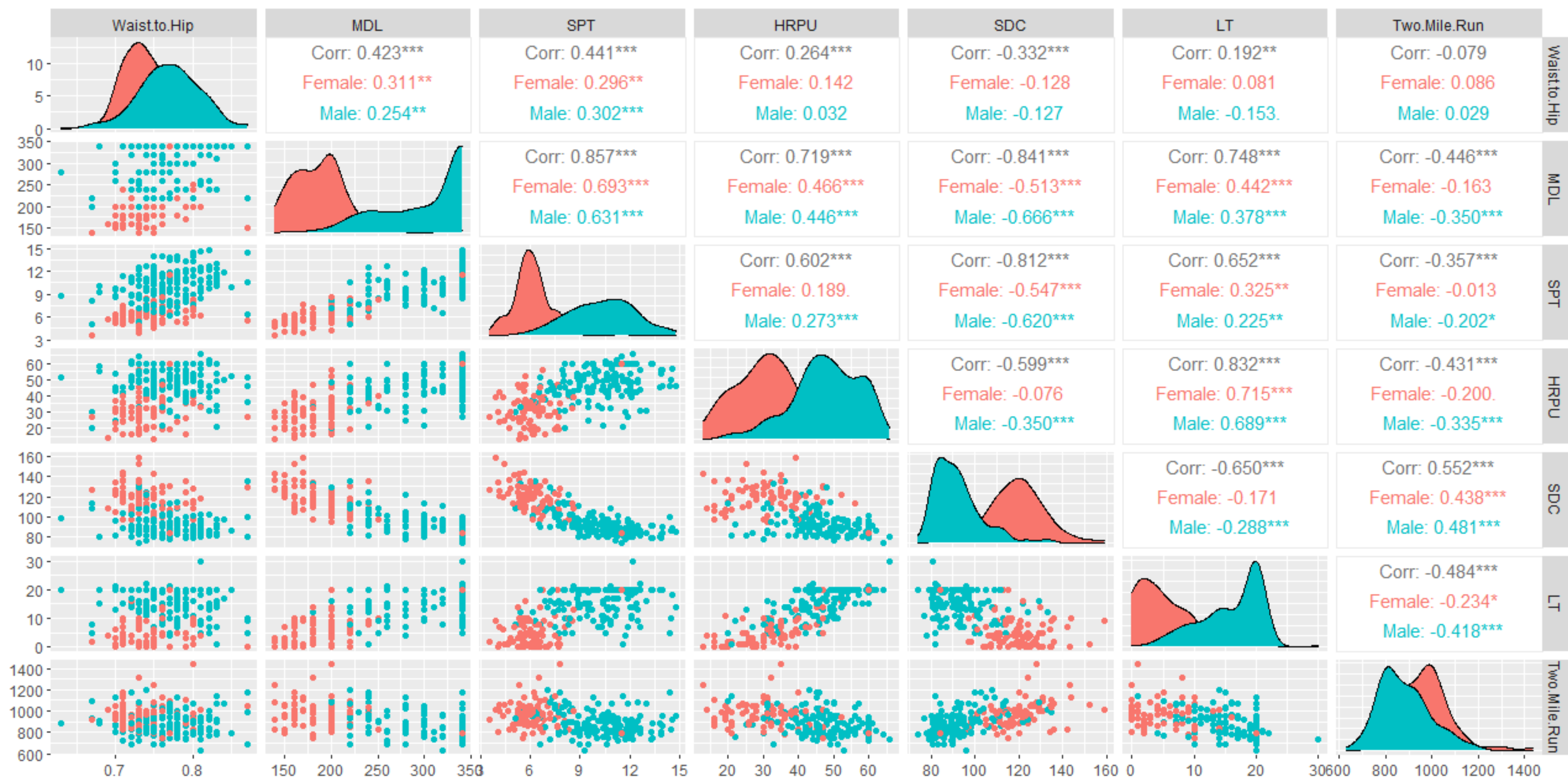

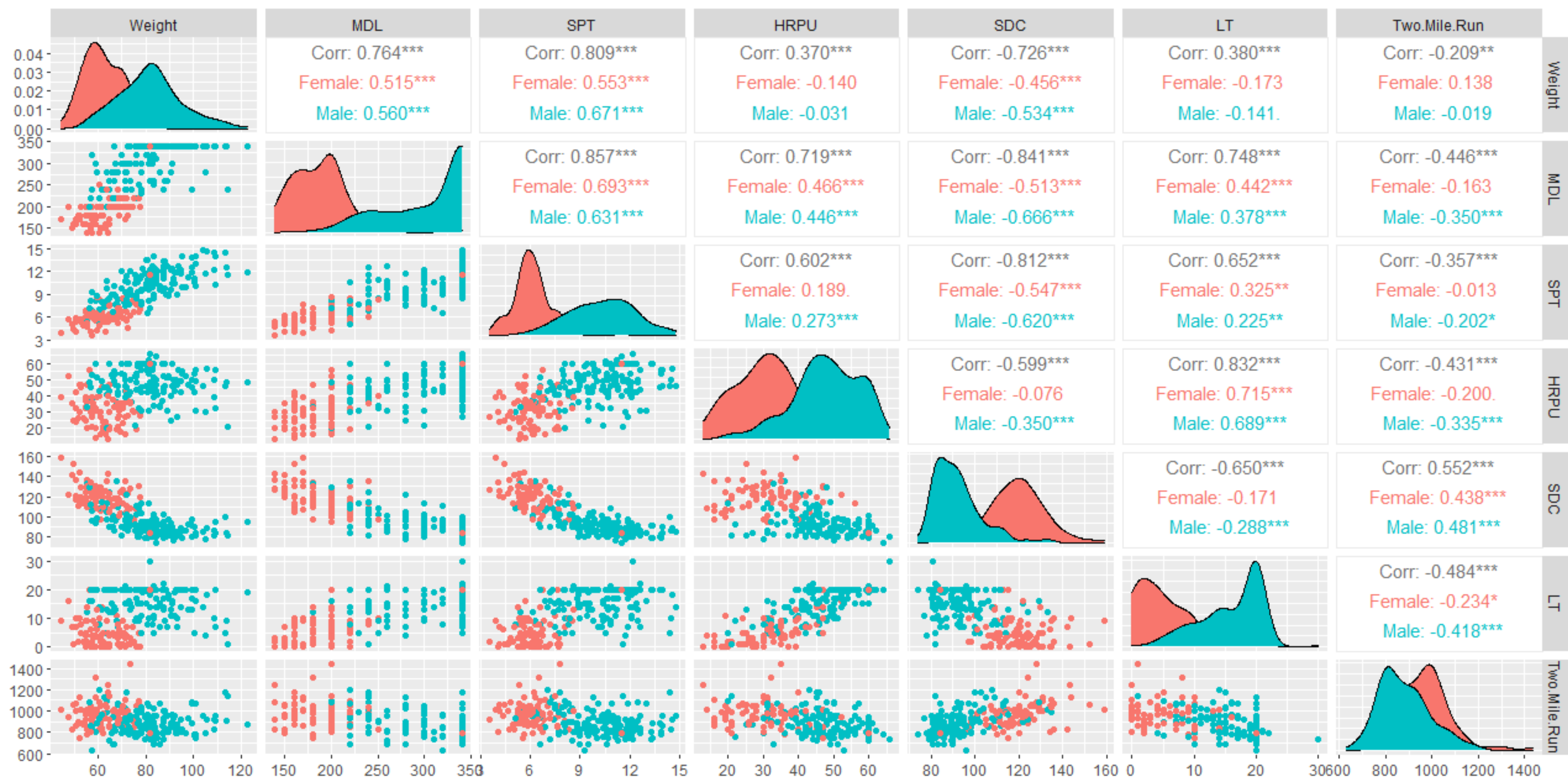

Supplement: S2 File — (PDF) [file pone.0283566.s002.pdf]

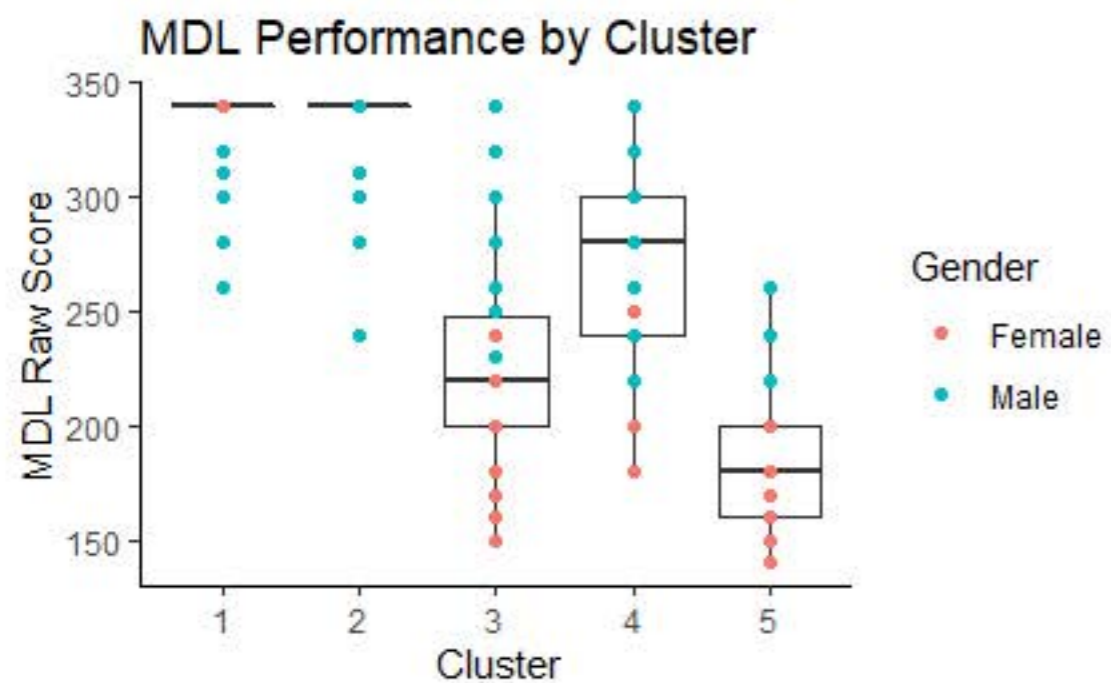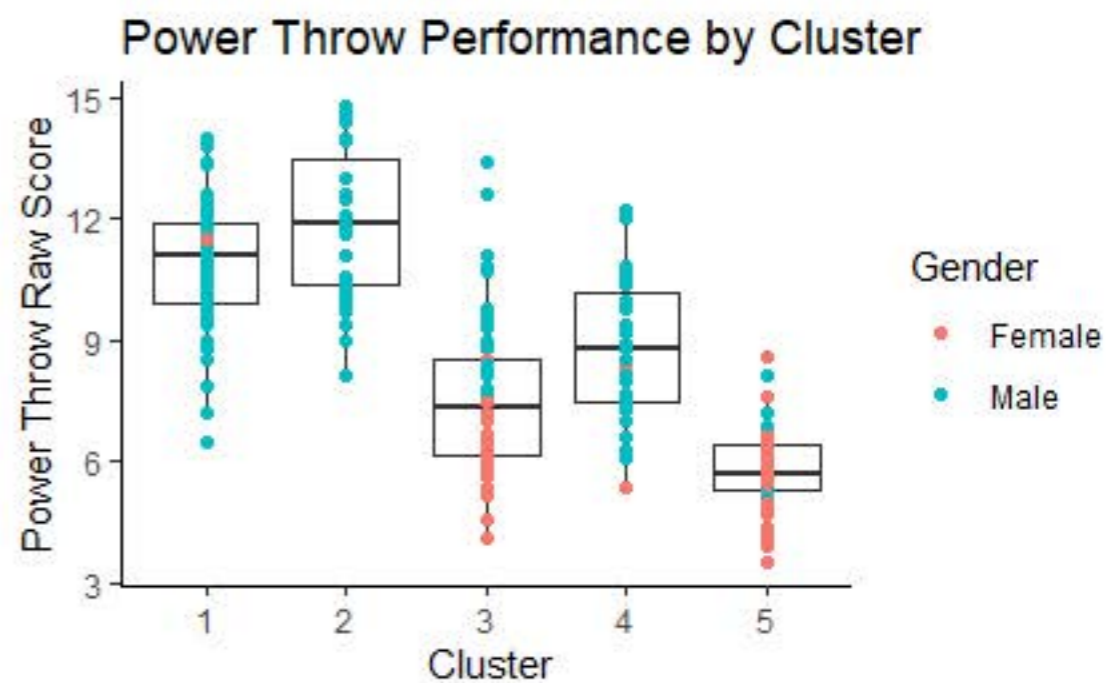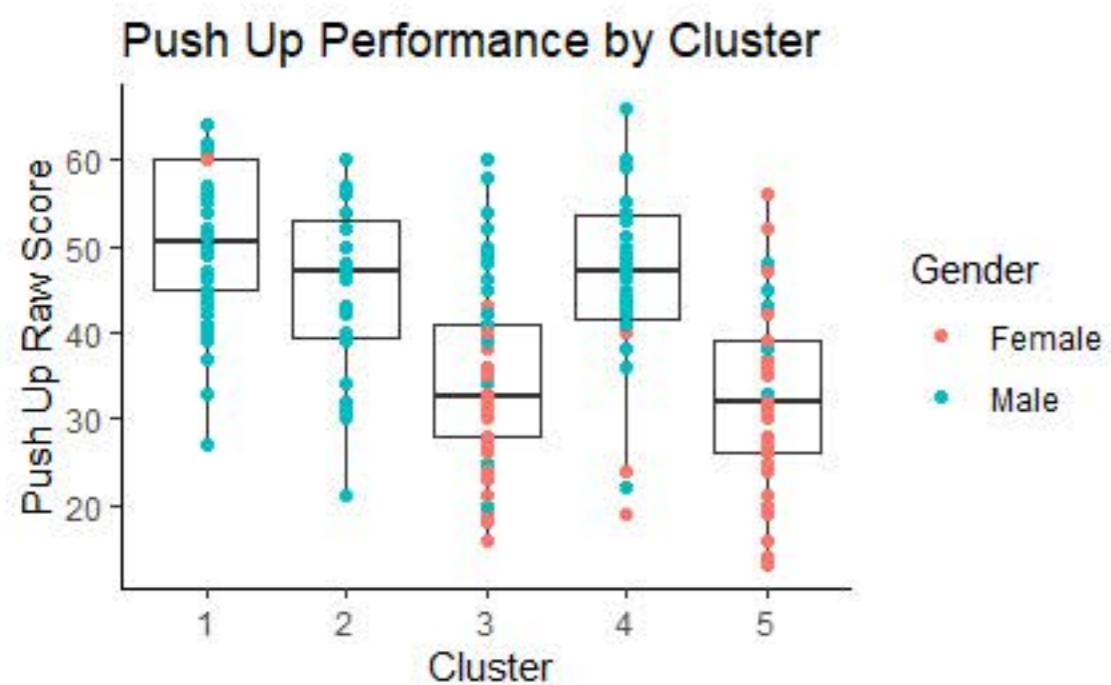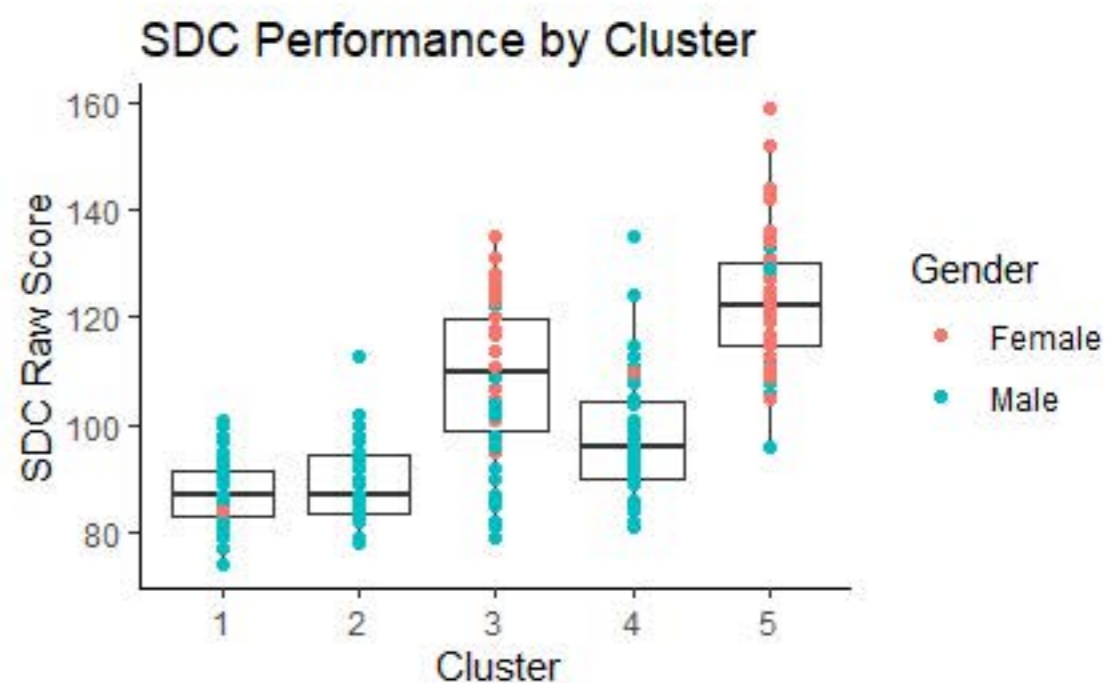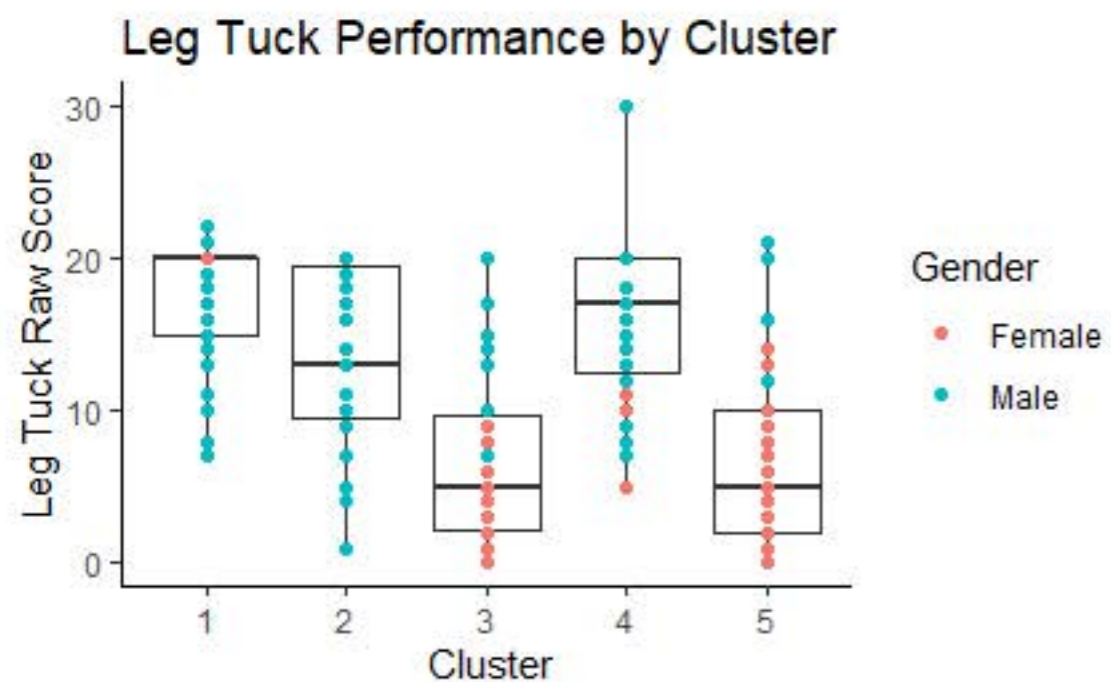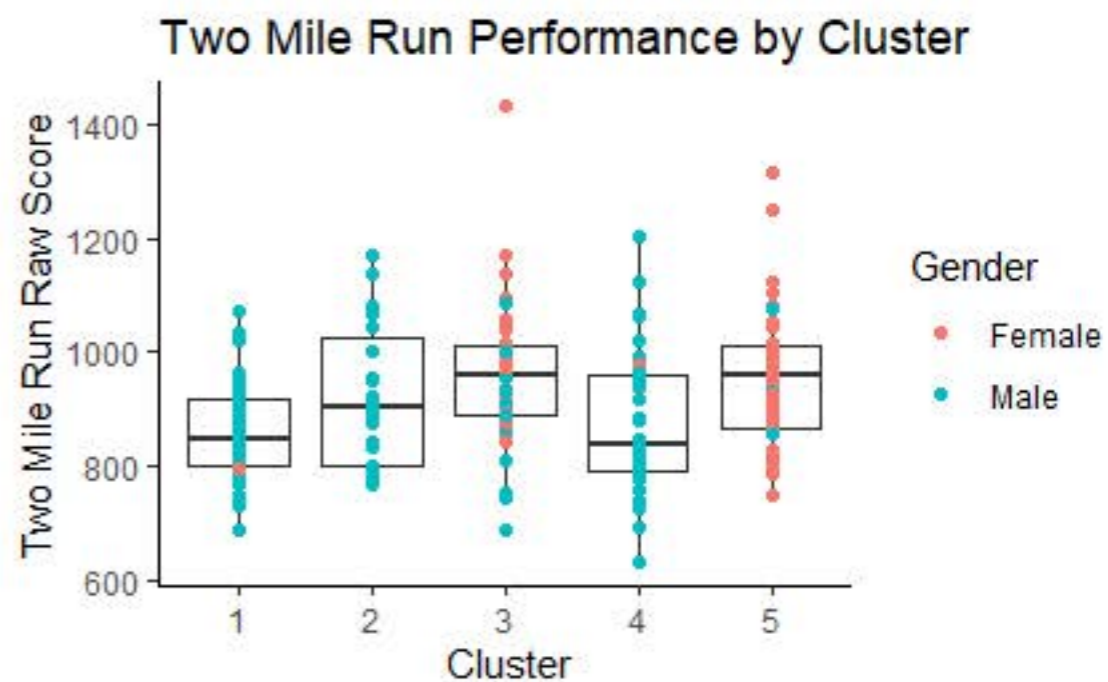

Supplement: S3 File — (PDF) [file pone.0283566.s003.pdf]
