# Supplementary material for: Body shape and performance on the US Army Combat Fitness Test: Insights from a 3D body image scanner
Source: PLoS One. 2023 May 3;18(5):e0283566. doi: 10.1371/journal.pone.0283566 (PMC10155989; doi:10.1371/journal.pone.0283566)
Supplement: S4 File — The elbow of the scree plot occurs at 5 clusters. (DOCX) [file pone.0283566.s004.docx]

**Scree plot developed from k-means cluster analysis. The elbow of the graph occurs at approximately 5 clusters.**

**
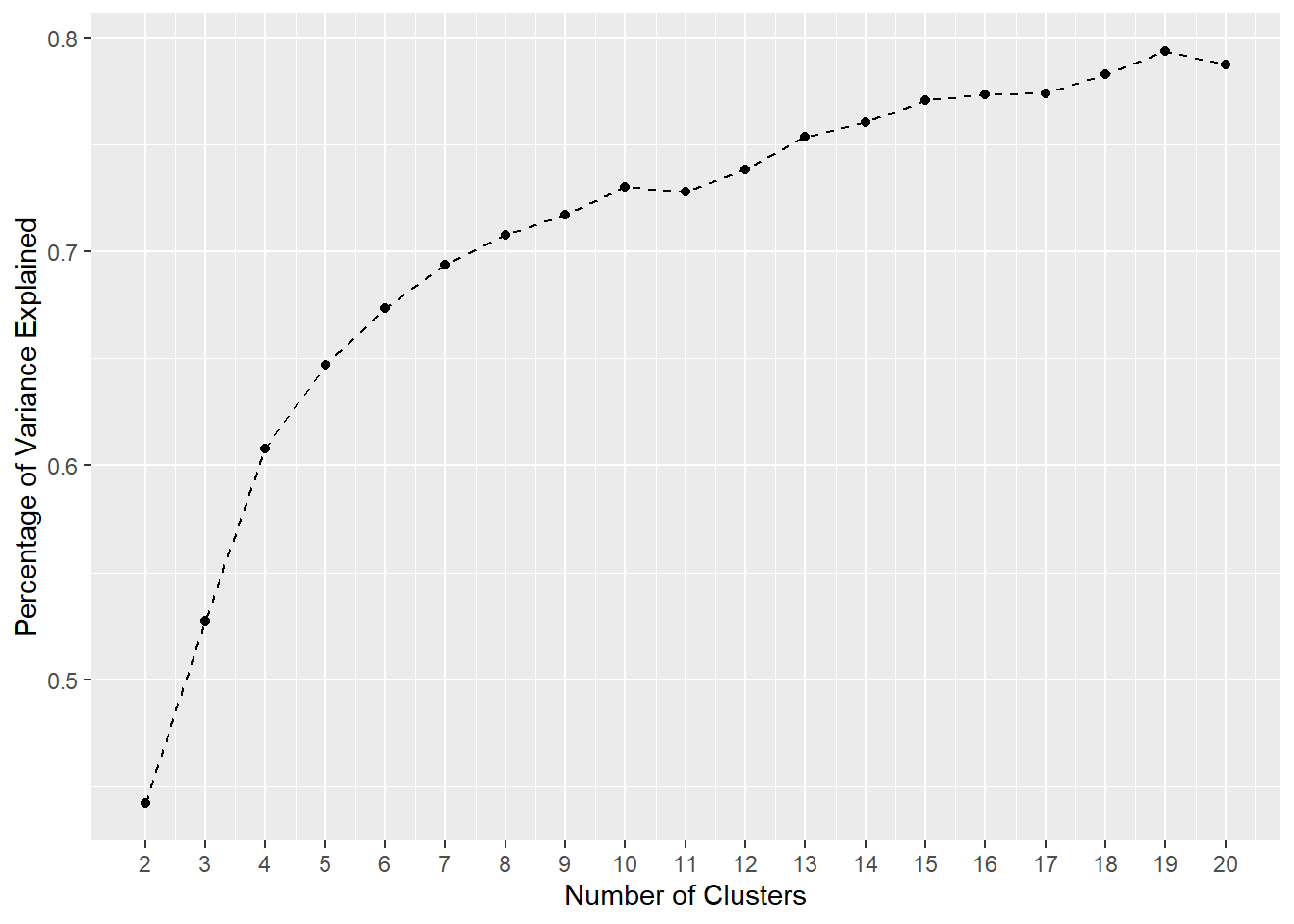
**
