# Supplementary material for: Body shape and performance on the US Army Combat Fitness Test: Insights from a 3D body image scanner
Source: PLoS One. 2023 May 3;18(5):e0283566. doi: 10.1371/journal.pone.0283566 (PMC10155989; doi:10.1371/journal.pone.0283566)
Supplement: S5 File — (PDF) [file pone.0283566.s005.pdf]

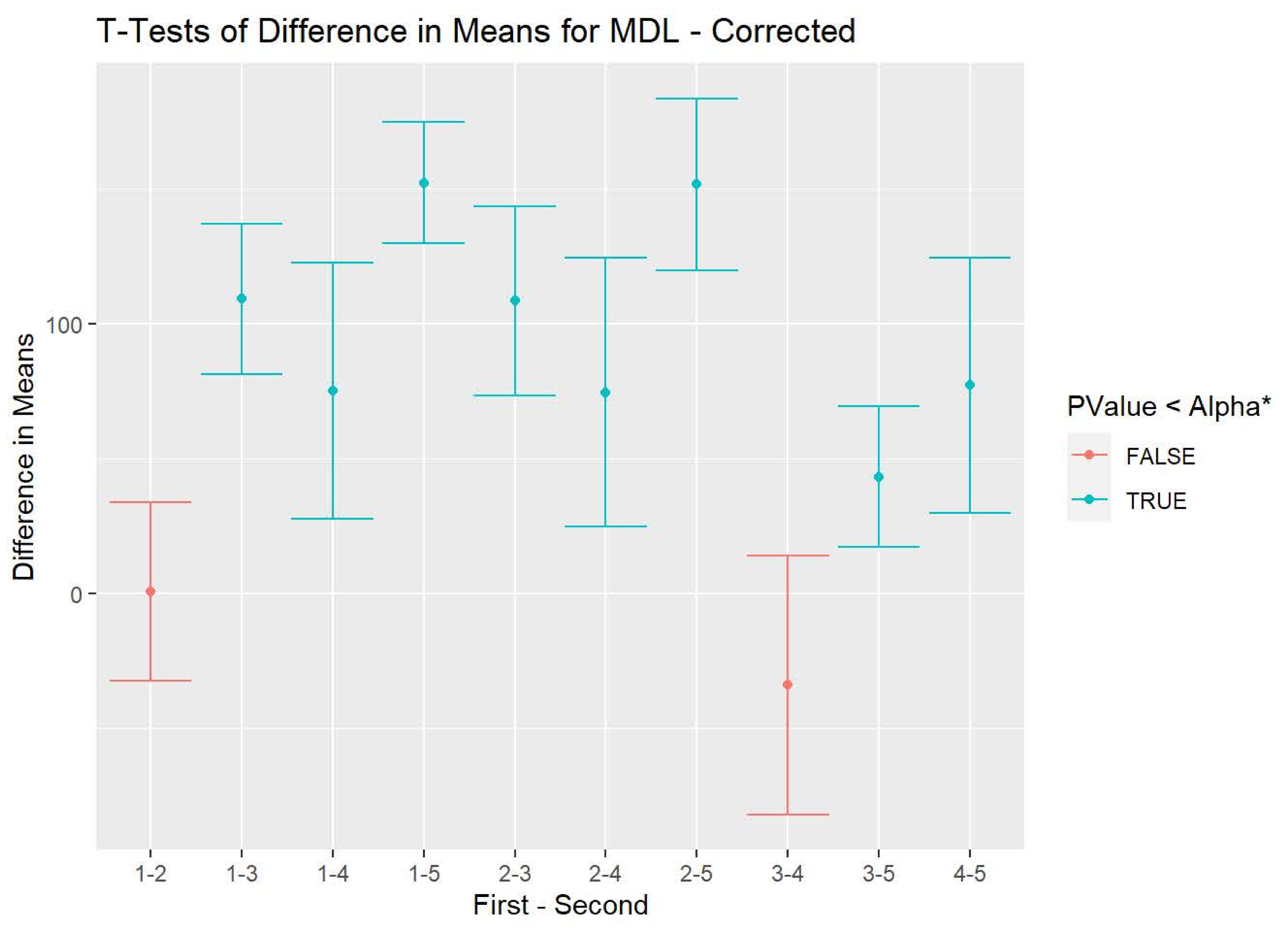
T-Tests of Difference in Means for MDL - Corrected
Difference in Means
100
0
1-2 1-3 1-4 1-5 2-3 2-4 2-5 3-4 3-5 4-5
First - Second
PValue < Alpha*
FALSE
TRUE

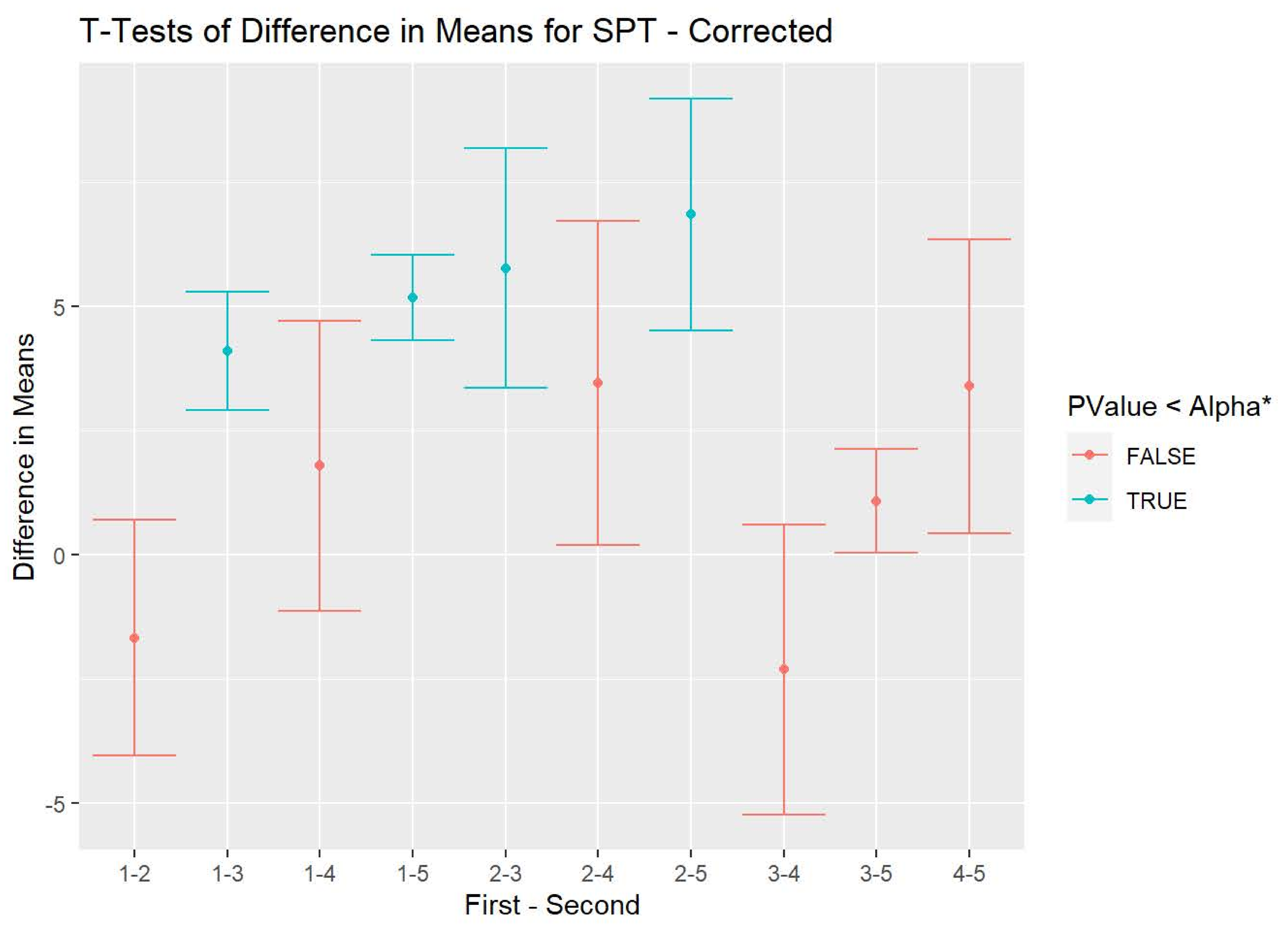
T-Tests of Difference in Means for SPT - Corrected
Difference in Means
5
0
-5
1-2 1-3 1-4 1-5 2-3 2-4 2-5 3-4 3-5 4-5
First - Second
PValue < Alpha*
FALSE
TRUE

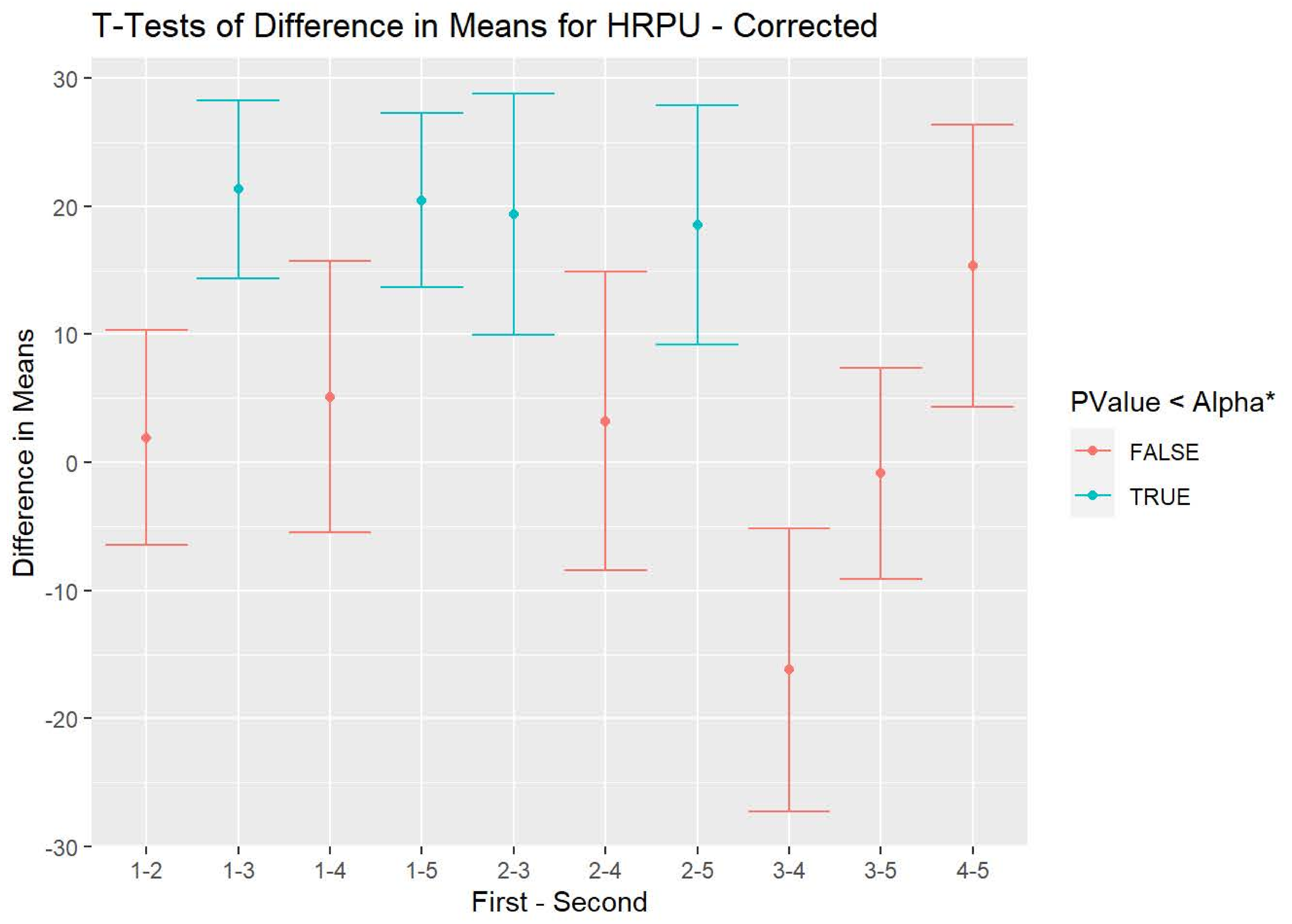
T-Tests of Difference in Means for HRPU - Corrected
Difference in Means
30
20
10
0
-10
-20
-30
1-2 1-3 1-4 1-5 2-3 2-4 2-5 3-4 3-5 4-5
First - Second
PValue < Alpha*
FALSE
TRUE

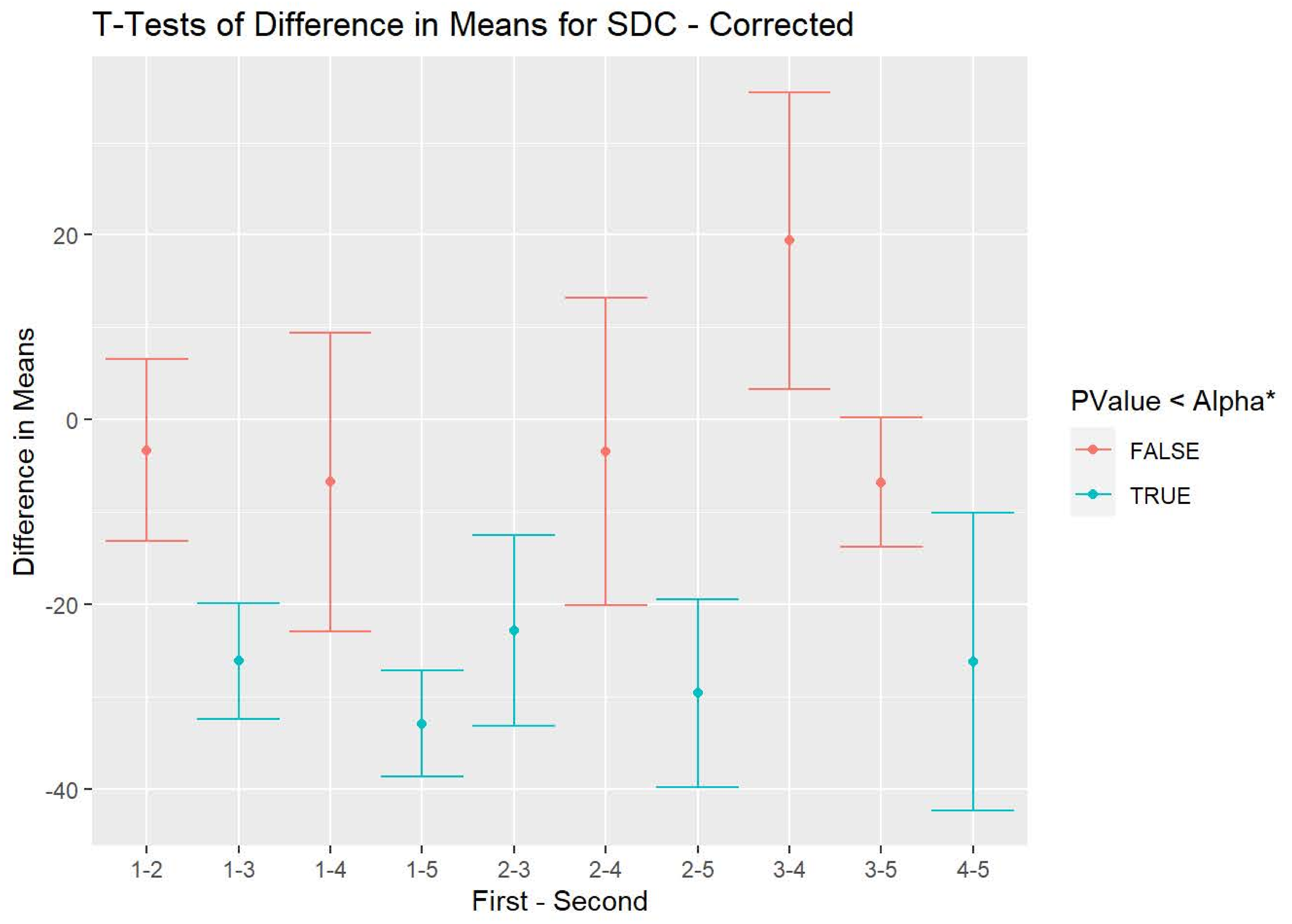
T-Tests of Difference in Means for SDC - Corrected
Difference in Means
20
0
-20
-40
1-2 1-3 1-4 1-5 2-3 2-4 2-5 3-4 3-5 4-5
First - Second
PValue < Alpha*
FALSE
TRUE

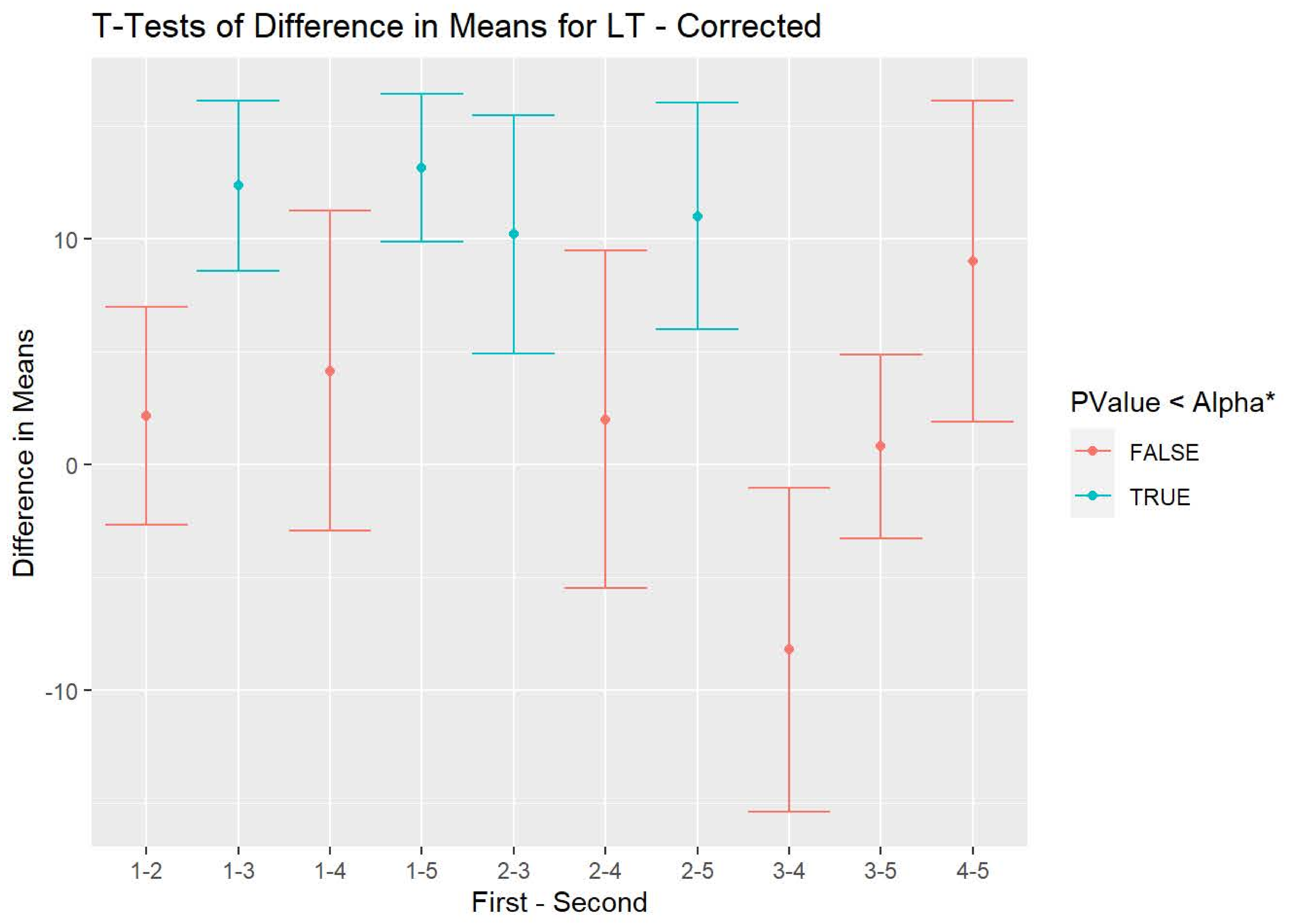
T-Tests of Difference in Means for LT - Corrected
Difference in Means
10
0
-10
1-2 1-3 1-4 1-5 2-3 2-4 2-5 3-4 3-5 4-5
First - Second
PValue < Alpha*
FALSE
TRUE

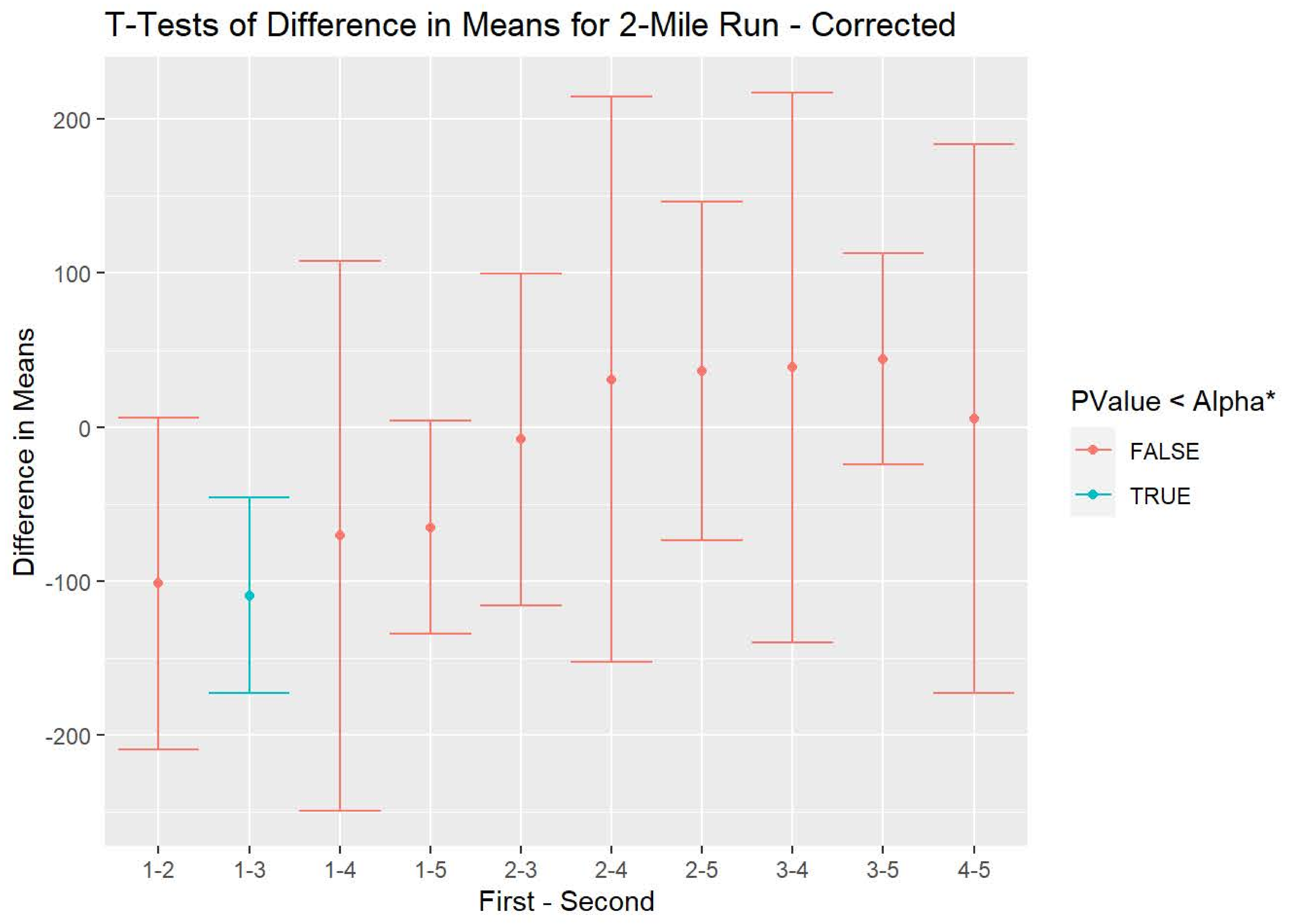
T-Tests of Difference in Means for 2-Mile Run - Corrected
Difference in Means
200
100
0
-100
-200
1-2 1-3 1-4 1-5 2-3 2-4 2-5 3-4 3-5 4-5
First - Second
PValue < Alpha*
FALSE
TRUE
