# Supplementary material for: Body shape and performance on the US Army Combat Fitness Test: Insights from a 3D body image scanner
Source: PLoS One. 2023 May 3;18(5):e0283566. doi: 10.1371/journal.pone.0283566 (PMC10155989; doi:10.1371/journal.pone.0283566)
Supplement: S6 File — (PDF) [file pone.0283566.s006.pdf]

Cluster Regression Analysis

MAJ Maria Smith

2023-02-03

Regression models with clusters and sex

Dead Lift

```
deadlift_model <- df %>%
  lm(dead_lift_raw_scr ~ cluster+sex, data = .)
summary(deadlift_model)
```

```
##
## Call:
## lm(formula = dead_lift_raw_scr ~ cluster + sex, data = .)
##
## Residuals:
##     Min      1Q  Median      3Q     Max
## -90.613 -12.227   7.773  11.172  87.944
##
## Coefficients:
##             Estimate Std. Error t value Pr(>|t|)
## (Intercept)  272.862      7.353   37.11   <2e-16 ***
## cluster      -20.807      1.585  -13.13   <2e-16 ***
## sexMale       80.171      5.076   15.79   <2e-16 ***
## ---
## Signif. codes:  0 '***' 0.001 '**' 0.01 '*' 0.05 '.' 0.1 ' ' 1
##
## Residual standard error: 30.13 on 236 degrees of freedom
## Multiple R-squared:  0.813,  Adjusted R-squared:  0.8114
## F-statistic:   513 on 2 and 236 DF,  p-value: < 2.2e-16
```

Sprint Drag Carry

```
SDC_model <- df %>%
  lm(sprint_drag_carry_raw_score ~ cluster+sex, data = .)
summary(SDC_model)
```

```
##
## Call:
## lm(formula = sprint_drag_carry_raw_score ~ cluster + sex, data = .)
##
## Residuals:
##     Min      1Q  Median      3Q     Max
## -21.778  -7.018  -0.399   6.363  35.603
```

```
##
## Coefficients:
##             Estimate Std. Error t value Pr(>|t|)
## (Intercept) 100.3013     2.5076  40.000  < 2e-16 ***
## cluster       4.6192     0.5404   8.548 1.58e-15 ***
## sexMale     -18.5213     1.7311 -10.699  < 2e-16 ***
## ---
## Signif. codes:  0 '***' 0.001 '**' 0.01 '*' 0.05 '.' 0.1 ' ' 1
##
## Residual standard error: 10.27 on 236 degrees of freedom
## Multiple R-squared:  0.6584, Adjusted R-squared:  0.6555
## F-statistic: 227.4 on 2 and 236 DF,  p-value: < 2.2e-16
```

Leg Tuck

```
legtuck_model <- df %>%
  lm(leg_tuck_raw_score ~ cluster+sex, data = .)
summary(legtuck_model)
```

```
##
## Call:
## lm(formula = leg_tuck_raw_score ~ cluster + sex, data = .)
##
## Residuals:
##      Min       1Q   Median       3Q      Max
## -15.0221  -3.3411   0.1404   3.6589  15.1404
##
## Coefficients:
##             Estimate Std. Error t value Pr(>|t|)
## (Intercept)   6.4548     1.2042   5.360 1.97e-07 ***
## cluster      -0.3190     0.2595  -1.229     0.22
## sexMale      10.2053     0.8313  12.276  < 2e-16 ***
## ---
## Signif. codes:  0 '***' 0.001 '**' 0.01 '*' 0.05 '.' 0.1 ' ' 1
##
## Residual standard error: 4.934 on 236 degrees of freedom
## Multiple R-squared:  0.5257, Adjusted R-squared:  0.5217
## F-statistic: 130.8 on 2 and 236 DF,  p-value: < 2.2e-16
```

Hand Release Push Up

```
HRPU_model <- df %>%
  lm(pu_raw_score ~ cluster+sex, data = .)
summary(HRPU_model)
```

```
##
## Call:
## lm(formula = pu_raw_score ~ cluster + sex, data = .)
##
## Residuals:
##      Min       1Q   Median       3Q      Max
## -27.1685  -5.5382  -0.1685   7.3074  25.8060
```

```
##
## Coefficients:
##             Estimate Std. Error t value Pr(>|t|)
## (Intercept)  36.3838     2.3217  15.671   <2e-16 ***
## cluster      -1.2379     0.5003  -2.474   0.0141 *
## sexMale      14.2606     1.6028   8.897   <2e-16 ***
## ---
## Signif. codes:  0 '***' 0.001 '**' 0.01 '*' 0.05 '.' 0.1 ' ' 1
##
## Residual standard error: 9.513 on 236 degrees of freedom
## Multiple R-squared:  0.4204, Adjusted R-squared:  0.4155
## F-statistic:  85.6 on 2 and 236 DF,  p-value: < 2.2e-16
```

Standing Power Throw

```
powerthrow_model <- df %>%
  lm(power_throw_raw_scr ~ cluster+sex, data = .)
summary(powerthrow_model)
```

```
##
## Call:
## lm(formula = power_throw_raw_scr ~ cluster + sex, data = .)
##
## Residuals:
##     Min      1Q  Median      3Q     Max
## -4.7133 -1.0336 -0.0133  0.8964  4.3066
##
## Coefficients:
##             Estimate Std. Error t value Pr(>|t|)
## (Intercept)  9.09316    0.37813  24.048  < 2e-16 ***
## cluster     -0.71986    0.08149  -8.834 2.35e-16 ***
## sexMale      2.83999    0.26104  10.879  < 2e-16 ***
## ---
## Signif. codes:  0 '***' 0.001 '**' 0.01 '*' 0.05 '.' 0.1 ' ' 1
##
## Residual standard error: 1.549 on 236 degrees of freedom
## Multiple R-squared:  0.6689, Adjusted R-squared:  0.6661
## F-statistic: 238.4 on 2 and 236 DF,  p-value: < 2.2e-16
```

Two Mile Run

```
cardio_model <- df %>%
  lm(cardio_raw_score ~ cluster+sex, data = .)
summary(cardio_model)
```

```
##
## Call:
## lm(formula = cardio_raw_score ~ cluster + sex, data = .)
##
## Residuals:
##     Min      1Q  Median      3Q     Max
## -263.07  -79.68   -9.07   68.44  473.79
```

```
##
## Coefficients:
##             Estimate Std. Error t value Pr(>|t|)
## (Intercept)  940.944     27.430  34.303  < 2e-16 ***
## cluster        6.755      5.911   1.143    0.254
## sexMale      -75.892     18.937  -4.008 8.22e-05 ***
## ---
## Signif. codes:  0 '***' 0.001 '**' 0.01 '*' 0.05 '.' 0.1 ' ' 1
##
## Residual standard error: 112.4 on 236 degrees of freedom
## Multiple R-squared:  0.1293, Adjusted R-squared:  0.1219
## F-statistic: 17.52 on 2 and 236 DF,  p-value: 8.047e-08
```

```
export_summs(deadlift_model, SDC_model, legtuck_model, HRPU_model, powerthrow_model, cardio_model, mode
```

	Max Deadlift	Sprint Drag Carry	Leg Tuck	Hand Release Push Up	Powe Throw	Two Mile Run
(Intercept)	212.36 ***	113.73 ***	5.53 ***	32.78 ***	7.00 ***	960.59 ***
	(3.84)	(1.31)	(0.63)	(1.21)	(0.20)	(14.34)
cluster	-31.80 ***	7.06 ***	-0.49	-1.89 *	-1.10 ***	10.32
	(2.42)	(0.83)	(0.40)	(0.76)	(0.12)	(9.03)
sexMale	80.17 ***	-18.52 ***	10.21 ***	14.26 ***	2.84 ***	-75.89 ***
	(5.08)	(1.73)	(0.83)	(1.60)	(0.26)	(18.94)
N	239	239	239	239	239	239
R2	0.81	0.66	0.53	0.42	0.67	0.13

All continuous predictors are mean-centered and scaled by 1 standard deviation. *** $p < 0.001$; ** $p < 0.01$; * $p < 0.05$.
