# Supplementary material for: Body shape and performance on the US Army Combat Fitness Test: Insights from a 3D body image scanner
Source: PLoS One. 2023 May 3;18(5):e0283566. doi: 10.1371/journal.pone.0283566 (PMC10155989; doi:10.1371/journal.pone.0283566)
Supplement: S7 File — (PDF) [file pone.0283566.s007.pdf]

No Cluster Models

Dusty Turner

8/17/2021

Without Cluster Models

Dead Lift

```
mod <-
as.formula(mdl~sex + height + bicep + lower_bicep + chest_to_hip_ratio + chest_to_waist_ratio + calf + 
  lm_helper(train = raw_data, test = raw_data, threshold = .05, model_obj = T)

summary(mod$model_object) %>%
  tidy() %>%
  arrange(p.value) %>%
  print(n = Inf)
```

```
## # A tibble: 21 x 5
##    term                 estimate std.error statistic     p.value
##    <chr>                   <dbl>     <dbl>     <dbl>       <dbl>
##  1 sexMale                45.3       8.58      5.28  0.000000319
##  2 bicep                   5.27      1.77      2.98  0.00323
##  3 lower_thigh             2.87      1.49      1.93  0.0550
##  4 chest_to_waist_ratio  563.      322.        1.75  0.0821
##  5 forearm                 3.43      2.02      1.69  0.0916
##  6 calf                   -1.40      0.989    -1.42  0.157
##  7 mid_thigh              -1.99      1.51     -1.32  0.188
##  8 lower_bicep            -2.94      2.53     -1.16  0.246
##  9 hip                    -6.21      5.51     -1.13  0.261
## 10 narrowest_waist         1.55      1.41      1.10  0.274
## 11 high_hip                1.87      1.81      1.03  0.303
## 12 lower_waist             4.12      4.16      0.991 0.323
## 13 weight                  0.586     0.607     0.965 0.335
## 14 chest_to_hip_ratio   -440.      503.       -0.876 0.382
## 15 height                  0.261     0.447     0.584 0.560
## 16 upper_thigh             0.330     0.849     0.389 0.698
## 17 neck                   -0.339     1.03     -0.327 0.744
## 18 waist_to_hip_ratio   -154.      516.       -0.299 0.765
## 19 (Intercept)          -123.      451.       -0.273 0.785
## 20 waist_abdominal         0.805     5.27      0.153 0.879
## 21 chest                  -0.431     3.96     -0.109 0.913
```

```
summary(mod$model_object) %>% broom::glance()
```

```
## # A tibble: 1 x 8
##   r.squared adj.r.squared sigma statistic  p.value    df df.residual  nobs
##       <dbl>         <dbl> <dbl>     <dbl>    <dbl> <dbl>       <int> <dbl>
```

```
## 1      0.845          0.831   28.5      59.5 1.00e-76     20          218    239
```

```
coefplot::coefplot(mod$model_object, intercept = F) +
  labs(title = "Coefficient Plot: Deadlift", x = "Standardized Coefficient", y = "") +
  scale_y_discrete(labels = c("weight" = "Weight","narrowest_waist" = "Narrowest Waist","lower_waist" =
                              "waist_to_hip_ratio" = "Waist to Hip","waist_abdominal" = "Waist Abdominal
                              "lower_thigh" = "Lower Thigh","neck" = "Neck","mid_thigh" = "Mid Thigh","
                              "forearm" = "Forearm","chest" = "Chest","calf" = "Calf","chest_to_waist_r
                              "chest_to_hip_ratio" = "Chest to Hip","lower_bicep" = "Lower Bicep","bice
```

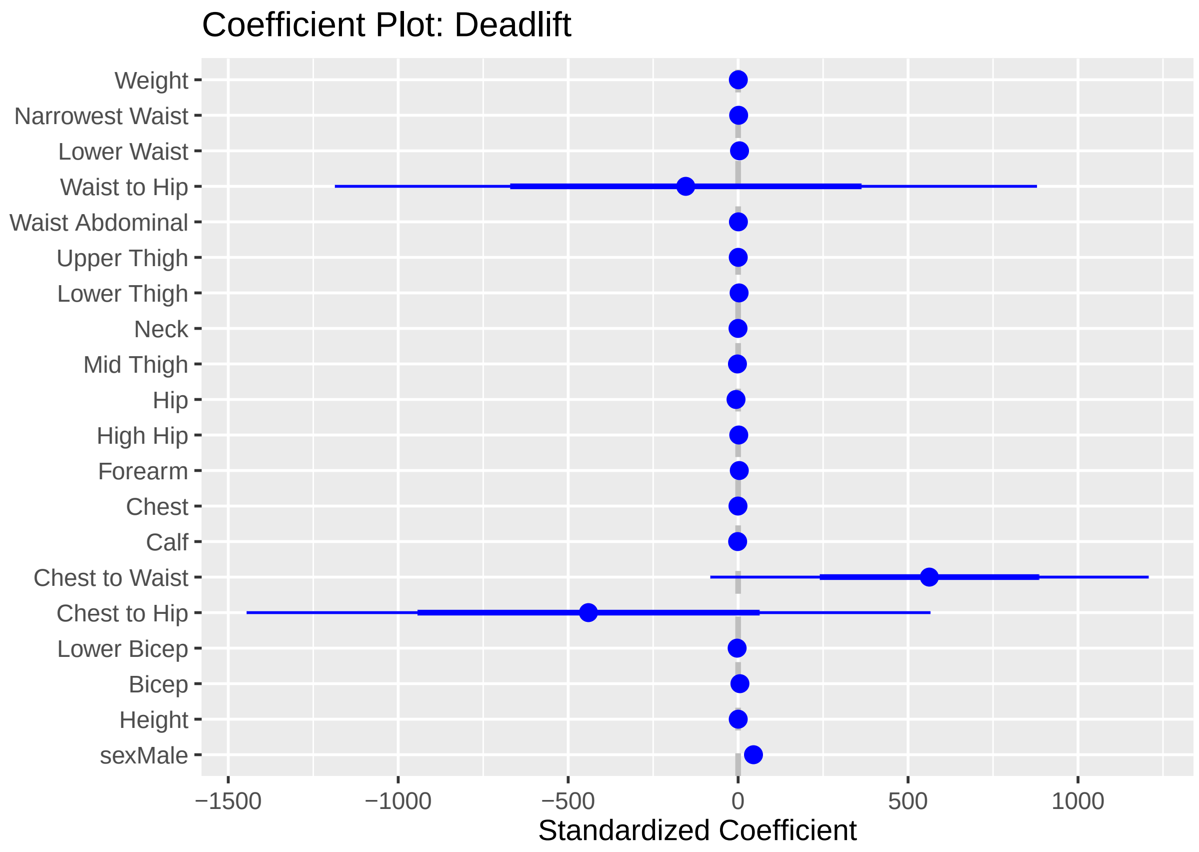

Sprint Drag Carry

```
mod <-
as.formula(sdc~sex + height + bicep + lower_bicep + chest_to_hip_ratio + chest_to_waist_ratio + calf +
  lm_helper(train = raw_data, test = raw_data, threshold = .05, model_obj = T)

summary(mod$model_object) %>%
  tidy() %>%
  arrange(p.value) %>%
  print(n = Inf)
```

```
## # A tibble: 21 x 5
##    term                     estimate std.error statistic   p.value
##    <chr>                       <dbl>     <dbl>     <dbl>     <dbl>
##  1 height                    -0.617      0.146    -4.23   0.0000337
##  2 (Intercept)              533.       147.        3.62   0.000362
##  3 sexMale                   -6.46       2.80     -2.31   0.0220
```

```
##  4 chest                    2.79         1.29    2.16    0.0316
##  5 lower_thigh             -0.955        0.485  -1.97    0.0504
##  6 forearm                 -1.19         0.660  -1.81    0.0716
##  7 chest_to_hip_ratio   -230.          164.     -1.40    0.162
##  8 hip                     -2.28         1.80   -1.27    0.206
##  9 narrowest_waist         -0.407        0.461  -0.882   0.379
## 10 upper_thigh              0.236        0.277   0.852   0.395
## 11 lower_bicep              0.554        0.824   0.673   0.502
## 12 chest_to_waist_ratio  -67.3         105.     -0.641   0.522
## 13 neck                    -0.130        0.337  -0.385   0.701
## 14 mid_thigh                0.129        0.491   0.264   0.792
## 15 bicep                   -0.149        0.577  -0.258   0.797
## 16 calf                     0.0716       0.322   0.222   0.825
## 17 waist_to_hip_ratio      35.2        168.      0.209   0.835
## 18 waist_abdominal         -0.157        1.72   -0.0913  0.927
## 19 lower_waist             -0.0891       1.36   -0.0657  0.948
## 20 high_hip                 0.0279       0.590   0.0474  0.962
## 21 weight                   0.000599     0.198   0.00303 0.998
```

```
summary(mod$model_object) %>% broom::glance()
```

```
## # A tibble: 1 x 8
##   r.squared adj.r.squared sigma statistic  p.value    df df.residual  nobs
##       <dbl>         <dbl> <dbl>     <dbl>    <dbl> <dbl>       <int> <dbl>
## 1     0.741         0.718  9.30      31.2 6.09e-53    20         218   239
```

```
coefplot::coefplot(mod$model_object, intercept = F) +
    labs(title = "Coefficient Plot: Sprint Drag Carry", x = "Standardized Coefficient", y = "") +
  scale_y_discrete(labels = c("weight" = "Weight","narrowest_waist" = "Narrowest Waist","lower_waist" =
                              "waist_to_hip_ratio" = "Waist to Hip","waist_abdominal" = "Waist Abdomina
                              "lower_thigh" = "Lower Thigh","neck" = "Neck","mid_thigh" = "Mid Thigh","
                              "forearm" = "Forearm","chest" = "Chest","calf" = "Calf","chest_to_waist_r
                              "chest_to_hip_ratio" = "Chest to Hip","lower_bicep" = "Lower Bicep","bice
```

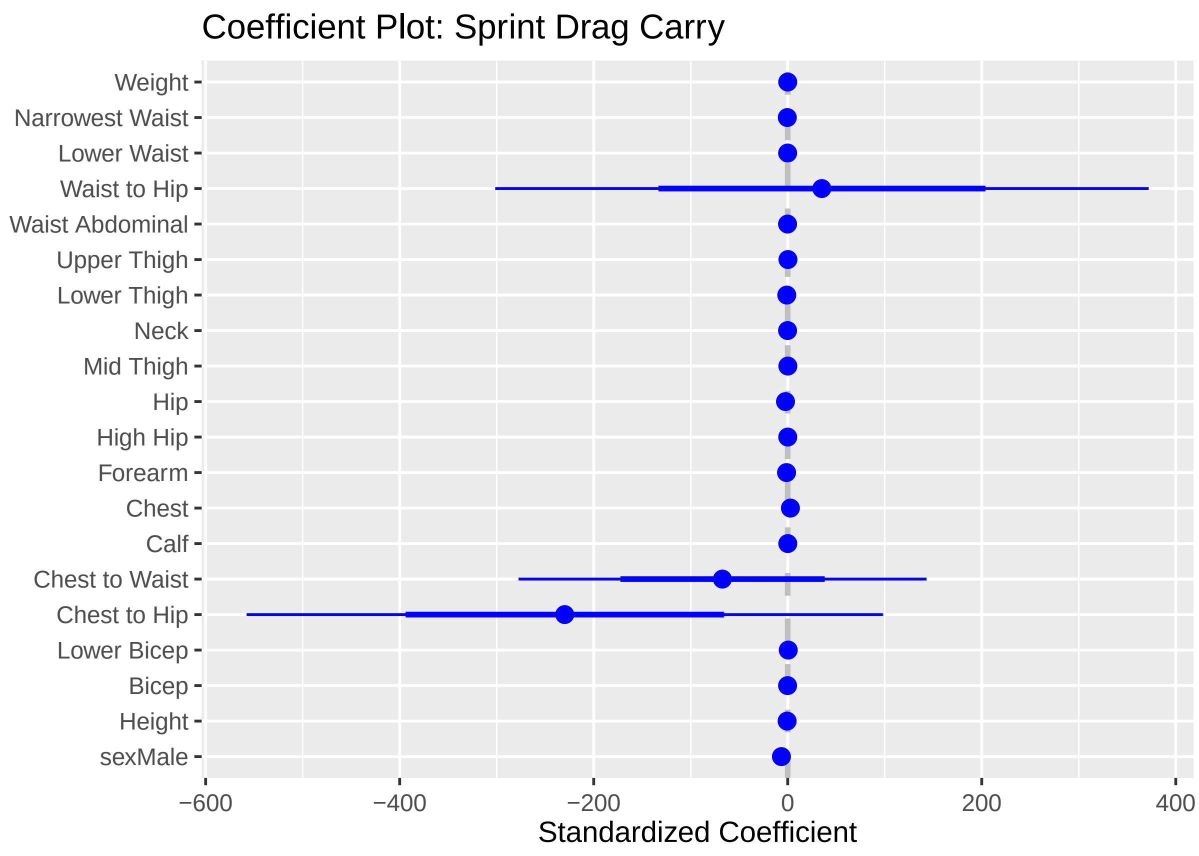

Leg Tuck

```
mod <-
as.formula(lt~sex + height + bicep + lower_bicep + chest_to_hip_ratio + chest_to_waist_ratio + calf + cl
  lm_helper(train = raw_data, test = raw_data, threshold = .05, model_obj = T)

summary(mod$model_object) %>%
  tidy() %>%
  arrange(p.value) %>%
  print(n = Inf)
```

```
## # A tibble: 21 x 5
##    term                  estimate std.error statistic     p.value
##    <chr>                    <dbl>     <dbl>     <dbl>       <dbl>
##  1 sexMale                 6.35      1.23       5.16   0.000000544
##  2 bicep                   1.02      0.254      4.00   0.0000859
##  3 forearm                 0.658     0.290      2.27   0.0243
##  4 lower_bicep            -0.818     0.362     -2.26   0.0249
##  5 mid_thigh              -0.452     0.216     -2.09   0.0375
##  6 calf                   -0.269     0.142     -1.90   0.0589
##  7 weight                 -0.141     0.0871    -1.62   0.106
##  8 lower_thigh             0.324     0.213      1.52   0.130
##  9 high_hip                0.366     0.259      1.41   0.160
## 10 upper_thigh            -0.128     0.122     -1.05   0.296
## 11 height                  0.0459    0.0641     0.717  0.474
## 12 narrowest_waist         0.122     0.203      0.603  0.547
```

```
## 13 lower_waist           -0.300     0.597     -0.503   0.615
## 14 chest_to_hip_ratio    34.7      72.1        0.481   0.631
## 15 neck                  -0.0668    0.148     -0.450   0.653
## 16 waist_to_hip_ratio   -27.6      74.0       -0.373   0.710
## 17 (Intercept)          -21.7      64.7       -0.335   0.738
## 18 chest_to_waist_ratio  11.3      46.2        0.245   0.807
## 19 chest                 -0.130     0.568     -0.229   0.819
## 20 hip                    0.0542    0.790      0.0685  0.945
## 21 waist_abdominal        0.0382    0.756      0.0505  0.960
```

```
summary(mod$model_object) %>% broom::glance()
```

```
## # A tibble: 1 x 8
##   r.squared adj.r.squared sigma statistic  p.value    df df.residual  nobs
##       <dbl>         <dbl> <dbl>     <dbl>    <dbl> <dbl>       <int> <dbl>
## 1     0.699         0.671  4.09      25.3 5.68e-46    20         218   239
```

```
coefplot::coefplot(mod$model_object, intercept = F) +
    labs(title = "Coefficient Plot: Leg Tuck", x = "Standardized Coefficient", y = "") +
  scale_y_discrete(labels = c("weight" = "Weight","narrowest_waist" = "Narrowest Waist","lower_waist" =
                              "waist_to_hip_ratio" = "Waist to Hip","waist_abdominal" = "Waist Abdomina
                              "lower_thigh" = "Lower Thigh","neck" = "Neck","mid_thigh" = "Mid Thigh","
                              "forearm" = "Forearm","chest" = "Chest","calf" = "Calf","chest_to_waist_r
                              "chest_to_hip_ratio" = "Chest to Hip","lower_bicep" = "Lower Bicep","bice
```

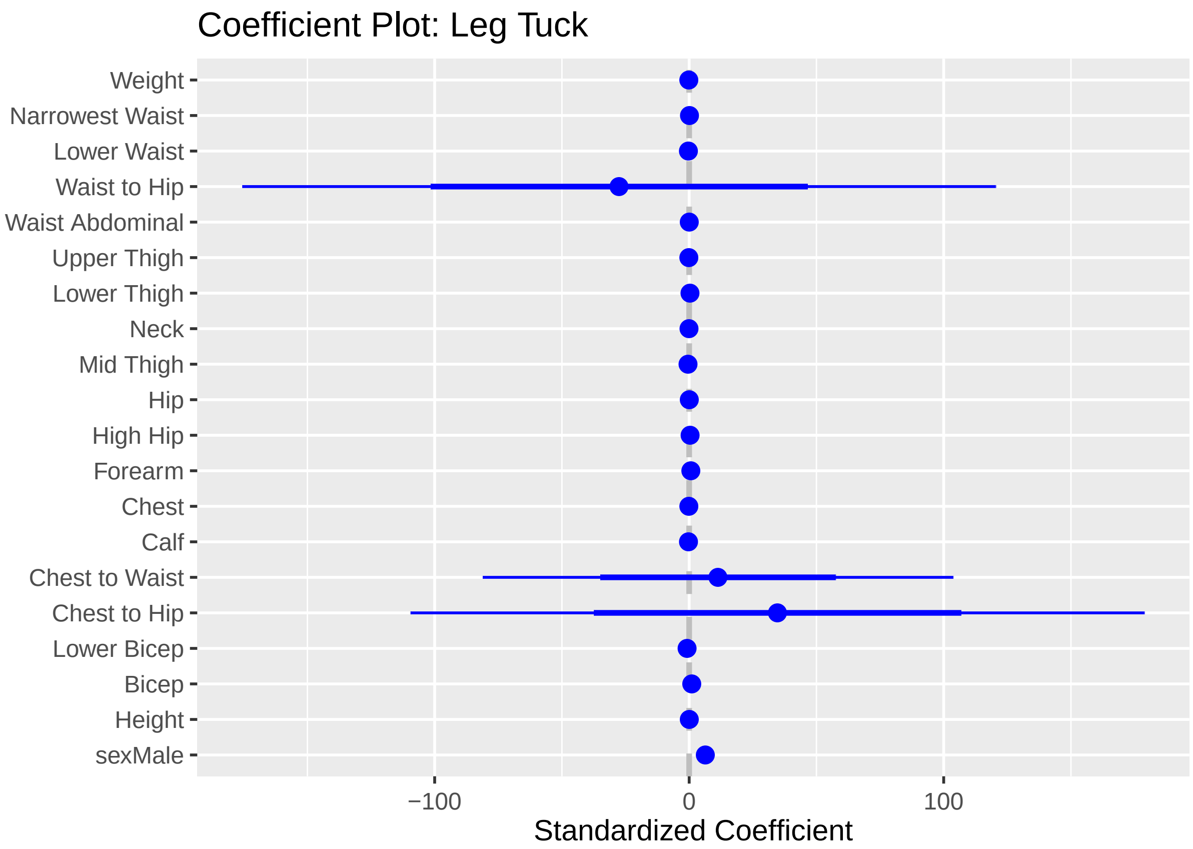

Hand Release Push Up

```
mod <-
as.formula(hrpu~sex + height + bicep + lower_bicep + chest_to_hip_ratio + chest_to_waist_ratio + calf +
  lm_helper(train = raw_data, test = raw_data, threshold = .05, model_obj = T)

summary(mod$model_object) %>%
  tidy() %>%
  arrange(p.value) %>%
  print(n = Inf)
```

```
## # A tibble: 21 x 5
##    term                 estimate std.error statistic   p.value
##    <chr>                   <dbl>     <dbl>     <dbl>     <dbl>
##  1 bicep                  2.09       0.497    4.21   0.0000377
##  2 sexMale                7.60       2.41     3.15   0.00186
##  3 upper_thigh           -0.508      0.239   -2.13   0.0342
##  4 lower_bicep           -1.40       0.710   -1.97   0.0506
##  5 forearm                1.07       0.569    1.87   0.0622
##  6 chest_to_waist_ratio  149.        90.5      1.65   0.101
##  7 narrowest_waist        0.618      0.397    1.55   0.121
##  8 neck                  -0.440      0.291   -1.51   0.132
##  9 height                -0.176      0.126   -1.40   0.163
## 10 weight                -0.229      0.171   -1.34   0.181
## 11 high_hip               0.455      0.508    0.896  0.371
## 12 lower_waist            1.03       1.17     0.878  0.381
## 13 chest_to_hip_ratio   -111.       141.      -0.787  0.432
## 14 hip                   -1.05       1.55    -0.676  0.500
## 15 mid_thigh             -0.196      0.423   -0.463  0.644
## 16 lower_thigh            0.193      0.418    0.462  0.645
## 17 calf                  -0.0660     0.278   -0.238  0.812
## 18 waist_abdominal       -0.296      1.48    -0.200  0.842
## 19 chest                 -0.150      1.11    -0.135  0.893
## 20 (Intercept)          -10.1      127.      -0.0793 0.937
## 21 waist_to_hip_ratio    -1.55     145.      -0.0107 0.992
```

```
summary(mod$model_object) %>% broom::glance()
```

```
## # A tibble: 1 x 8
##   r.squared adj.r.squared sigma statistic  p.value    df df.residual  nobs
##       <dbl>         <dbl> <dbl>     <dbl>    <dbl> <dbl>       <int> <dbl>
## 1     0.620         0.585  8.02      17.8 2.10e-35    20         218   239
```

```
coefplot::coefplot(mod$model_object, intercept = F) +
    labs(title = "Coefficient Plot: Hand Release Push Up", x = "Standardized Coefficient", y = "") +
  scale_y_discrete(labels = c("weight" = "Weight","narrowest_waist" = "Narrowest Waist","lower_waist" =
                              "waist_to_hip_ratio" = "Waist to Hip","waist_abdominal" = "Waist Abdomina
                              "lower_thigh" = "Lower Thigh","neck" = "Neck","mid_thigh" = "Mid Thigh","
                              "forearm" = "Forearm","chest" = "Chest","calf" = "Calf","chest_to_waist_r
                              "chest_to_hip_ratio" = "Chest to Hip","lower_bicep" = "Lower Bicep","bice
```

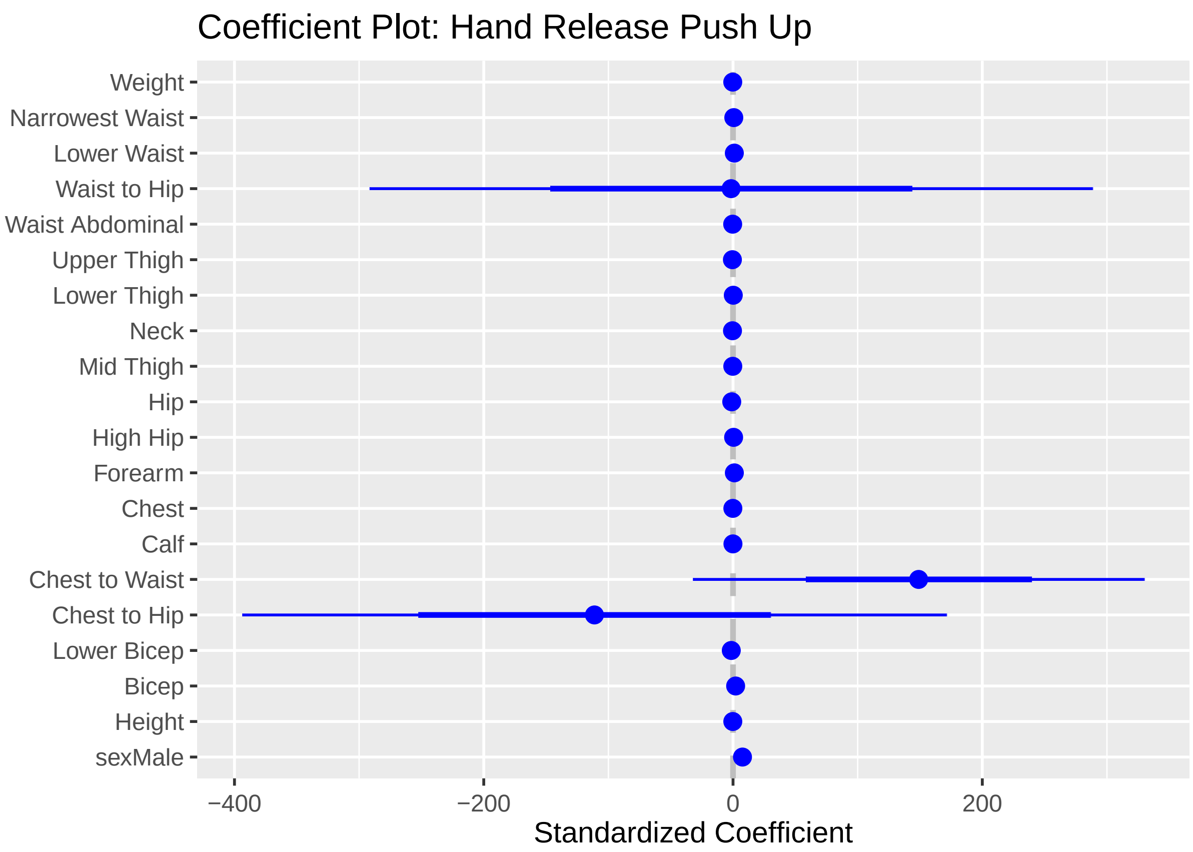

Standing Power Throw

```
mod <-
as.formula(spr~sex + height + bicep + lower_bicep + chest_to_hip_ratio + chest_to_waist_ratio + calf + c
  lm_helper(train = raw_data, test = raw_data, threshold = .05, model_obj = T)

summary(mod$model_object) %>%
  tidy() %>%
  arrange(p.value) %>%
  print(n = Inf)
```

```
## # A tibble: 21 x 5
##    term                 estimate std.error statistic p.value
##    <chr>                   <dbl>     <dbl>     <dbl>   <dbl>
##  1 sexMale               1.09       0.388      2.82   0.00530
##  2 height                0.0569     0.0202     2.82   0.00531
##  3 forearm               0.226      0.0915     2.47   0.0144
##  4 weight                0.0465     0.0275     1.69   0.0923
##  5 chest_to_waist_ratio 23.8       14.6        1.64   0.103
##  6 hip                  -0.329      0.249     -1.32   0.188
##  7 upper_thigh          -0.0463     0.0384    -1.21   0.229
##  8 lower_waist           0.209      0.188      1.11   0.269
##  9 lower_bicep          -0.120      0.114     -1.05   0.295
## 10 calf                 -0.0412     0.0447    -0.921  0.358
## 11 waist_abdominal       0.210      0.238      0.883  0.378
## 12 chest_to_hip_ratio  -18.5       22.7       -0.813  0.417
```

```
## 13 waist_to_hip_ratio   -16.5       23.3      -0.706  0.481
## 14 neck                   0.0329      0.0468     0.704  0.482
## 15 mid_thigh              0.0449      0.0681     0.658  0.511
## 16 high_hip               0.0533      0.0818     0.652  0.515
## 17 lower_thigh            0.0388      0.0673     0.577  0.565
## 18 bicep                  0.0419      0.0800     0.524  0.601
## 19 chest                 -0.0681      0.179     -0.380  0.704
## 20 (Intercept)           -6.64       20.4      -0.325  0.745
## 21 narrowest_waist        0.00468     0.0640     0.0732 0.942
```

```
summary(mod$model_object) %>% broom::glance()
```

```
## # A tibble: 1 x 8
##   r.squared adj.r.squared sigma statistic  p.value    df df.residual  nobs
##       <dbl>         <dbl> <dbl>     <dbl>    <dbl> <dbl>       <int> <dbl>
## 1     0.788         0.768  1.29      40.5 4.15e-62    20         218   239
```

```
coefplot::coefplot(mod$model_object, intercept = F) +
    labs(title = "Coefficient Plot: Standing Power Throw", x = "Standardized Coefficient", y = "") +
  scale_y_discrete(labels = c("weight" = "Weight","narrowest_waist" = "Narrowest Waist","lower_waist" =
                              "waist_to_hip_ratio" = "Waist to Hip","waist_abdominal" = "Waist Abdomina
                              "lower_thigh" = "Lower Thigh","neck" = "Neck","mid_thigh" = "Mid Thigh","
                              "forearm" = "Forearm","chest" = "Chest","calf" = "Calf","chest_to_waist_r
                              "chest_to_hip_ratio" = "Chest to Hip","lower_bicep" = "Lower Bicep","bice
```

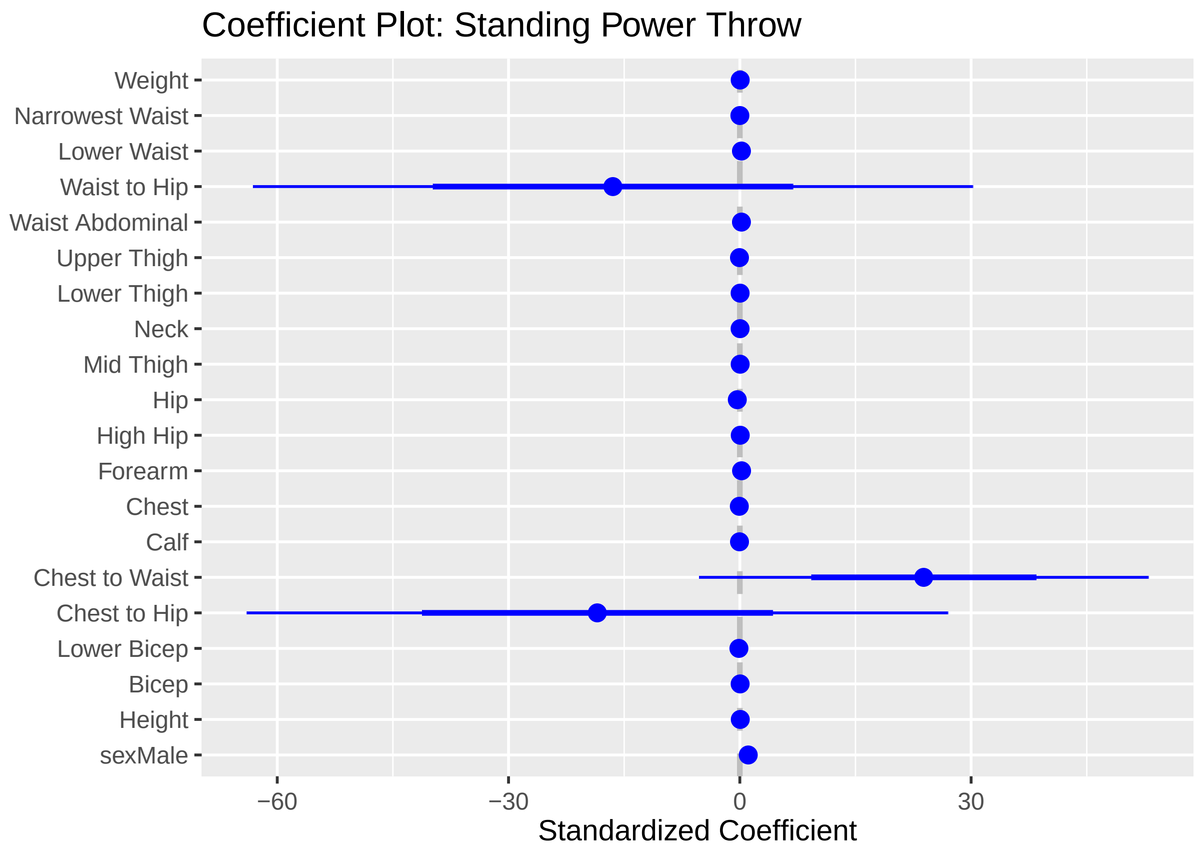

Two Mile Run

```
mod <-
as.formula(two_mile_run~sex + height + bicep + lower_bicep + chest_to_hip_ratio + chest_to_waist_ratio +
  lm_helper(train = raw_data, test = raw_data, threshold = .05, model_obj = T)

summary(mod$model_object) %>%
  tidy() %>%
  arrange(p.value) %>%
  print(n = Inf)
```

```
## # A tibble: 21 x 5
##    term                 estimate std.error statistic  p.value
##    <chr>                   <dbl>     <dbl>     <dbl>    <dbl>
##  1 height                  -5.52      1.63   -3.38   0.000845
##  2 neck                   -11.8       3.77   -3.12   0.00207
##  3 lower_thigh            -15.1       5.43   -2.78   0.00595
##  4 upper_thigh              6.36      3.10    2.05   0.0412
##  5 (Intercept)           3226.     1646.      1.96   0.0514
##  6 mid_thigh                9.50      5.50    1.73   0.0855
##  7 lower_bicep             12.5       9.22    1.35   0.178
##  8 bicep                   -7.50      6.46   -1.16   0.246
##  9 chest                   16.0      14.4     1.10   0.270
## 10 high_hip                 5.76      6.60    0.873  0.384
## 11 narrowest_waist         -4.05      5.16   -0.786  0.433
## 12 chest_to_waist_ratio  -898.     1175.     -0.764  0.445
## 13 calf                     2.58      3.61    0.715  0.476
## 14 hip                    -14.1      20.1    -0.703  0.483
## 15 sexMale                 19.4      31.3     0.620  0.536
## 16 weight                   1.07      2.22    0.484  0.629
## 17 chest_to_hip_ratio    -661.     1834.     -0.360  0.719
## 18 lower_waist             -5.39     15.2    -0.355  0.723
## 19 forearm                  1.51      7.38    0.205  0.838
## 20 waist_abdominal          2.01     19.2     0.104  0.917
## 21 waist_to_hip_ratio      77.3    1883.      0.0411 0.967
```

```
summary(mod$model_object) %>% broom::glance()
```

```
## # A tibble: 1 x 8
##   r.squared adj.r.squared sigma statistic  p.value    df df.residual  nobs
##       <dbl>         <dbl> <dbl>     <dbl>    <dbl> <dbl>       <int> <dbl>
## 1     0.310         0.247  104.      4.90 7.71e-10    20         218   239
```

```
coefplot::coefplot(mod$model_object, intercept = F) +
    labs(title = "Coefficient Plot: Two Mile Run", x = "Standardized Coefficient", y = "") +
  scale_y_discrete(labels = c("weight" = "Weight","narrowest_waist" = "Narrowest Waist","lower_waist" =
                            "waist_to_hip_ratio" = "Waist to Hip","waist_abdominal" = "Waist Abdominal
                            "lower_thigh" = "Lower Thigh","neck" = "Neck","mid_thigh" = "Mid Thigh","
                            "forearm" = "Forearm","chest" = "Chest","calf" = "Calf","chest_to_waist_ra
                            "chest_to_hip_ratio" = "Chest to Hip","lower_bicep" = "Lower Bicep","bicep
```

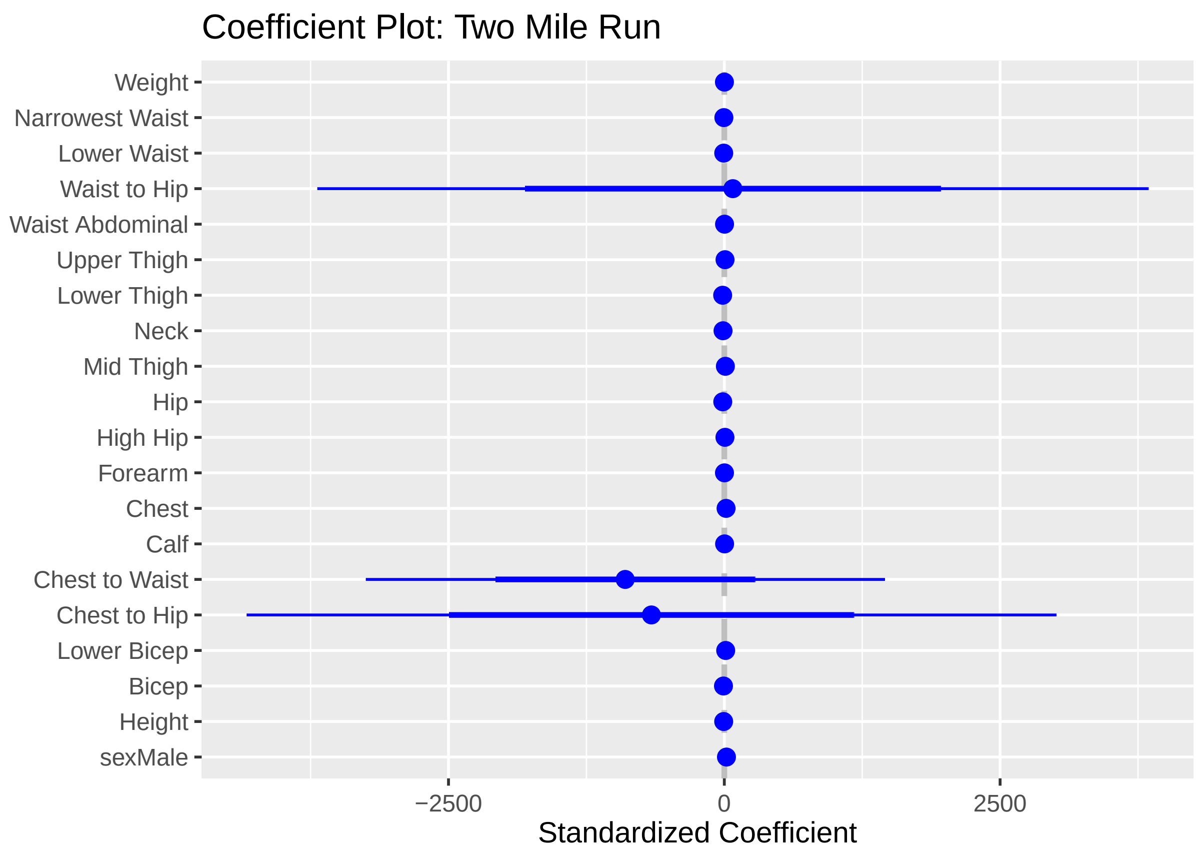

Without Cluster Standardized Models

Dead Lift

```
mod <-
as.formula(mdl~sex + height + bicep + lower_bicep + chest_to_hip_ratio + chest_to_waist_ratio + calf +
  lm_helper(train = raw_scaled, test = raw_scaled, threshold = .05, model_obj = T)

summary(mod$model_object) %>%
  tidy() %>%
  arrange(p.value) %>%
  print(n = Inf)
```

```
## # A tibble: 21 x 5
##    term                   estimate std.error statistic     p.value
##    <chr>                     <dbl>     <dbl>     <dbl>       <dbl>
##  1 sexMale                  0.652     0.124       5.28  0.000000319
##  2 (Intercept)             -0.426     0.0850     -5.01  0.00000112
##  3 bicep                    0.307     0.103       2.98  0.00323
##  4 lower_thigh              0.146     0.0757      1.93  0.0550
##  5 chest_to_waist_ratio     0.835     0.478       1.75  0.0821
##  6 forearm                  0.152     0.0896      1.69  0.0916
##  7 calf                    -0.0594    0.0418     -1.42  0.157
##  8 mid_thigh               -0.140     0.106      -1.32  0.188
##  9 lower_bicep             -0.135     0.116      -1.16  0.246
## 10 hip                     -0.589     0.523      -1.13  0.261
```

```
## 11 narrowest_waist        0.167     0.153      1.10  0.274
## 12 high_hip               0.169     0.164      1.03  0.303
## 13 lower_waist            0.385     0.388      0.991 0.323
## 14 weight                 0.127     0.131      0.965 0.335
## 15 chest_to_hip_ratio    -0.476     0.543     -0.876 0.382
## 16 height                 0.0366    0.0627     0.584 0.560
## 17 upper_thigh            0.0273    0.0702     0.389 0.698
## 18 neck                  -0.0188    0.0574    -0.327 0.744
## 19 waist_to_hip_ratio    -0.0893    0.299     -0.299 0.765
## 20 waist_abdominal        0.0789    0.516      0.153 0.879
## 21 chest                 -0.0588    0.539     -0.109 0.913
```

```
summary(mod$model_object) %>% broom::glance()
```

```
## # A tibble: 1 x 8
##   r.squared adj.r.squared sigma statistic  p.value    df df.residual  nobs
##       <dbl>         <dbl> <dbl>     <dbl>    <dbl> <dbl>       <int> <dbl>
## 1     0.845         0.831 0.411      59.5 1.00e-76    20         218   239
```

```
coefplot::coefplot(mod$model_object, intercept = F) +
    labs(title = "Coefficient Plot: Deadlift", x = "Standardized Coefficient", y = "") +
  scale_y_discrete(labels = c("weight" = "Weight","narrowest_waist" = "Narrowest Waist","lower_waist" =
                              "waist_to_hip_ratio" = "Waist to Hip","waist_abdominal" = "Waist Abdominal
                              "lower_thigh" = "Lower Thigh","neck" = "Neck","mid_thigh" = "Mid Thigh","
                              "forearm" = "Forearm","chest" = "Chest","calf" = "Calf","chest_to_waist_ra
                              "chest_to_hip_ratio" = "Chest to Hip","lower_bicep" = "Lower Bicep","bicep
```

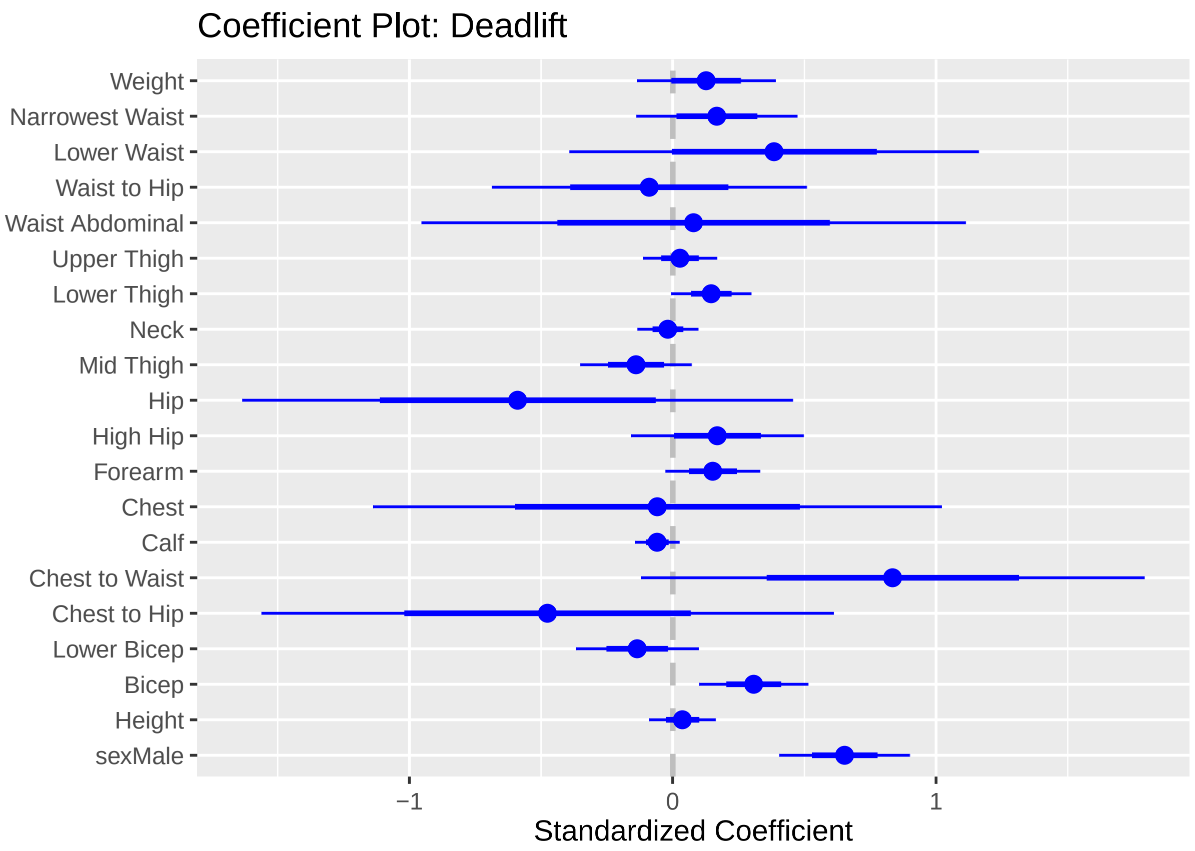

Sprint Drag Carry

```
mod <-
as.formula(sdc~sex + height + bicep + lower_bicep + chest_to_hip_ratio + chest_to_waist_ratio + calf + 
  lm_helper(train = raw_scaled, test = raw_scaled, threshold = .05, model_obj = T)

summary(mod$model_object) %>%
  tidy() %>%
  arrange(p.value) %>%
  print(n = Inf)
```

```
## # A tibble: 21 x 5
##    term                 estimate std.error statistic   p.value
##    <chr>                   <dbl>     <dbl>     <dbl>     <dbl>
##  1 height              -0.343       0.0811   -4.23    0.0000337
##  2 sexMale             -0.369       0.160    -2.31    0.0220
##  3 (Intercept)          0.241       0.110     2.19    0.0295
##  4 chest                1.51        0.697     2.16    0.0316
##  5 lower_thigh         -0.192       0.0978   -1.97    0.0504
##  6 forearm             -0.210       0.116    -1.81    0.0716
##  7 chest_to_hip_ratio  -0.984       0.702    -1.40    0.162
##  8 hip                 -0.858       0.675    -1.27    0.206
##  9 narrowest_waist     -0.174       0.197    -0.882   0.379
## 10 upper_thigh          0.0773      0.0907    0.852   0.395
## 11 lower_bicep          0.101       0.150     0.673   0.502
## 12 chest_to_waist_ratio -0.396      0.618    -0.641   0.522
## 13 neck                -0.0286      0.0742   -0.385   0.701
## 14 mid_thigh            0.0360      0.136     0.264   0.792
## 15 bicep               -0.0344      0.133    -0.258   0.797
## 16 calf                 0.0120      0.0541    0.222   0.825
## 17 waist_to_hip_ratio   0.0807      0.386     0.209   0.835
## 18 waist_abdominal     -0.0609      0.667    -0.0913  0.927
## 19 lower_waist         -0.0330      0.502    -0.0657  0.948
## 20 high_hip             0.0100      0.212     0.0474  0.962
## 21 weight               0.000514    0.170     0.00303 0.998
```

```
summary(mod$model_object) %>% broom::glance()
```

```
## # A tibble: 1 x 8
##   r.squared adj.r.squared sigma statistic  p.value    df df.residual  nobs
##       <dbl>         <dbl> <dbl>     <dbl>    <dbl> <dbl>       <int> <dbl>
## 1     0.741         0.718 0.531      31.2 6.09e-53    20         218   239
```

```
coefplot::coefplot(mod$model_object, intercept = F) +
  labs(title = "Coefficient Plot: Sprint Drag Carry", x = "Standardized Coefficient", y = "") +
  scale_y_discrete(labels = c("weight" = "Weight","narrowest_waist" = "Narrowest Waist","lower_waist" =
                              "waist_to_hip_ratio" = "Waist to Hip","waist_abdominal" = "Waist Abdomina
                              "lower_thigh" = "Lower Thigh","neck" = "Neck","mid_thigh" = "Mid Thigh","
                              "forearm" = "Forearm","chest" = "Chest","calf" = "Calf","chest_to_waist_r
                              "chest_to_hip_ratio" = "Chest to Hip","lower_bicep" = "Lower Bicep","bice
```

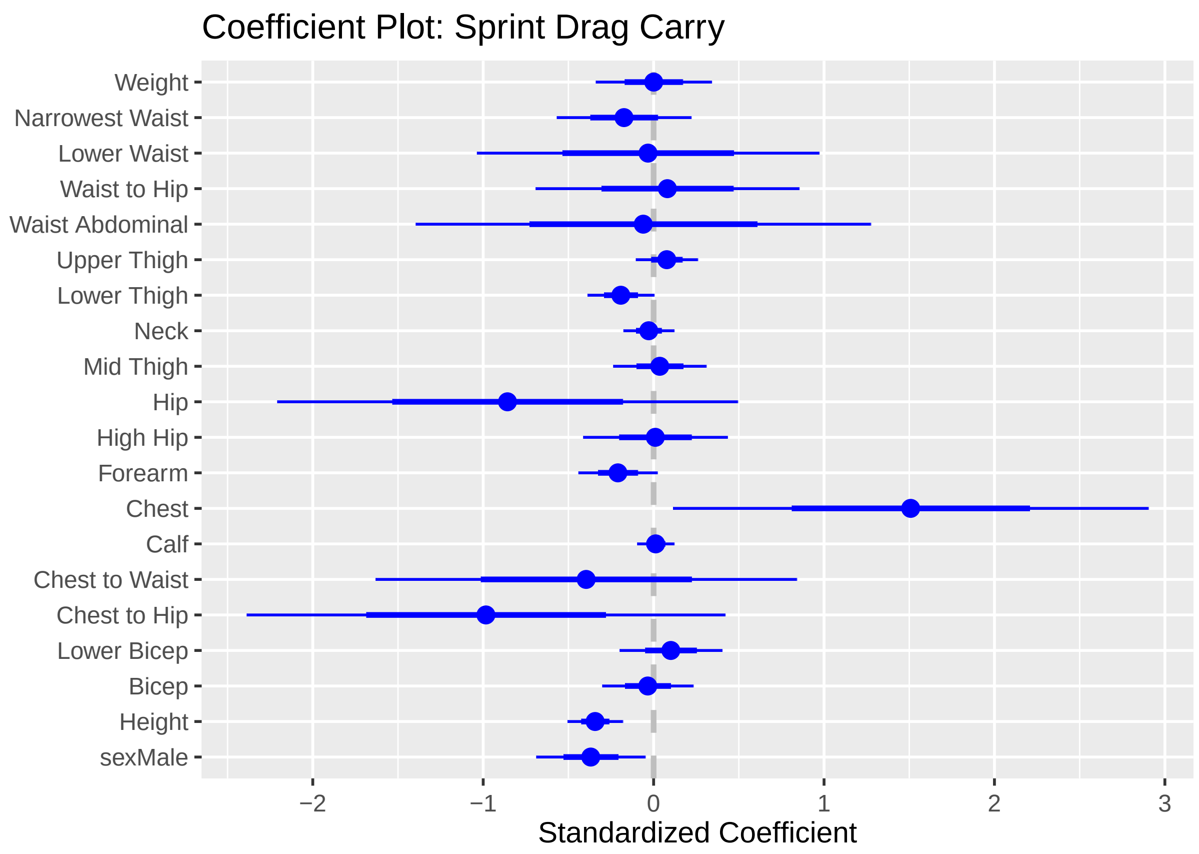

Leg Tuck

```
mod <-
as.formula(lt~sex + height + bicep + lower_bicep + chest_to_hip_ratio + chest_to_waist_ratio + calf + c
  lm_helper(train = raw_scaled, test = raw_scaled, threshold = .05, model_obj = T)

summary(mod$model_object) %>%
  tidy() %>%
  arrange(p.value) %>%
  print(n = Inf)
```

```
## # A tibble: 21 x 5
##    term                 estimate std.error statistic     p.value
##    <chr>                   <dbl>     <dbl>     <dbl>       <dbl>
##  1 sexMale               0.891      0.172      5.16   0.000000544
##  2 (Intercept)          -0.581      0.119     -4.90   0.00000183
##  3 bicep                 0.576      0.144      4.00   0.0000859
##  4 forearm               0.283      0.125      2.27   0.0243
##  5 lower_bicep          -0.366      0.162     -2.26   0.0249
##  6 mid_thigh            -0.308      0.147     -2.09   0.0375
##  7 calf                 -0.111      0.0583    -1.90   0.0589
##  8 weight               -0.297      0.183     -1.62   0.106
##  9 lower_thigh           0.160      0.106      1.52   0.130
## 10 high_hip              0.322      0.228      1.41   0.160
## 11 upper_thigh          -0.103      0.0979    -1.05   0.296
## 12 height                0.0627     0.0875     0.717  0.474
```

```
## 13 narrowest_waist         0.128     0.213     0.603  0.547
## 14 lower_waist            -0.272     0.542    -0.503  0.615
## 15 chest_to_hip_ratio      0.364     0.757     0.481  0.631
## 16 neck                   -0.0361    0.0801   -0.450  0.653
## 17 waist_to_hip_ratio     -0.155     0.417    -0.373  0.710
## 18 chest_to_waist_ratio    0.163     0.666     0.245  0.807
## 19 chest                  -0.172     0.752    -0.229  0.819
## 20 hip                     0.0499    0.729     0.0685 0.945
## 21 waist_abdominal         0.0364    0.720     0.0505 0.960
```

```
summary(mod$model_object) %>% broom::glance()
```

```
## # A tibble: 1 x 8
##   r.squared adj.r.squared sigma statistic  p.value    df df.residual  nobs
##       <dbl>         <dbl> <dbl>     <dbl>    <dbl> <dbl>       <int> <dbl>
## 1     0.699         0.671 0.573      25.3 5.68e-46    20         218   239
```

```
coefplot::coefplot(mod$model_object, intercept = F) +
  labs(title = "Coefficient Plot: Leg Tuck", x = "Standardized Coefficient", y = "") +
  scale_y_discrete(labels = c("weight" = "Weight","narrowest_waist" = "Narrowest Waist","lower_waist" =
                              "waist_to_hip_ratio" = "Waist to Hip","waist_abdominal" = "Waist Abdomina
                              "lower_thigh" = "Lower Thigh","neck" = "Neck","mid_thigh" = "Mid Thigh","
                              "forearm" = "Forearm","chest" = "Chest","calf" = "Calf","chest_to_waist_r
                              "chest_to_hip_ratio" = "Chest to Hip","lower_bicep" = "Lower Bicep","bice
```

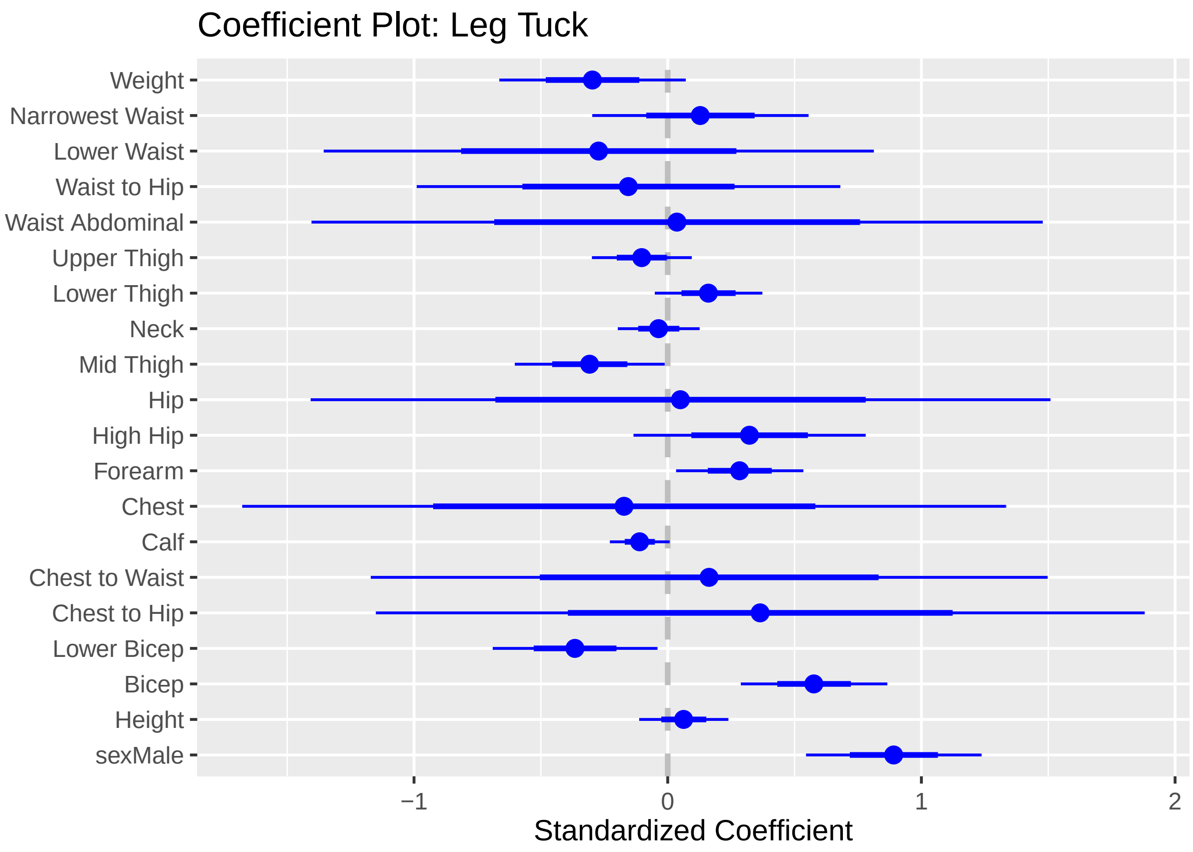

Hand Release Push Up

```
mod <-
as.formula(hrpu~sex + height + bicep + lower_bicep + chest_to_hip_ratio + chest_to_waist_ratio + calf +
  lm_helper(train = raw_scaled, test = raw_scaled, threshold = .05, model_obj = T)

summary(mod$model_object) %>%
  tidy() %>%
  arrange(p.value) %>%
  print(n = Inf)
```

```
## # A tibble: 21 x 5
##    term                 estimate std.error statistic   p.value
##    <chr>                   <dbl>     <dbl>     <dbl>     <dbl>
##  1 bicep                 0.680      0.162     4.21   0.0000377
##  2 sexMale               0.610      0.194     3.15   0.00186
##  3 (Intercept)          -0.398      0.133    -2.99   0.00310
##  4 upper_thigh          -0.234      0.110    -2.13   0.0342
##  5 lower_bicep          -0.358      0.182    -1.97   0.0506
##  6 forearm               0.263      0.140     1.87   0.0622
##  7 chest_to_waist_ratio  1.23       0.749     1.65   0.101
##  8 narrowest_waist       0.372      0.239     1.55   0.121
##  9 neck                 -0.136      0.0900   -1.51   0.132
## 10 height               -0.138      0.0983   -1.40   0.163
## 11 weight               -0.276      0.206    -1.34   0.181
## 12 high_hip              0.230      0.257     0.896  0.371
## 13 lower_waist           0.534      0.608     0.878  0.381
## 14 chest_to_hip_ratio   -0.670      0.851    -0.787  0.432
## 15 hip                  -0.553      0.819    -0.676  0.500
## 16 mid_thigh            -0.0765     0.165    -0.463  0.644
## 17 lower_thigh           0.0548     0.119     0.462  0.645
## 18 calf                 -0.0156     0.0656   -0.238  0.812
## 19 waist_abdominal      -0.162      0.809    -0.200  0.842
## 20 chest                -0.114      0.845    -0.135  0.893
## 21 waist_to_hip_ratio   -0.00500    0.469    -0.0107 0.992
```

```
summary(mod$model_object) %>% broom::glance()
```

```
## # A tibble: 1 x 8
##   r.squared adj.r.squared sigma statistic  p.value    df df.residual  nobs
##       <dbl>         <dbl> <dbl>     <dbl>    <dbl> <dbl>       <int> <dbl>
## 1     0.620         0.585 0.644      17.8 2.10e-35    20         218   239
```

```
coefplot::coefplot(mod$model_object, intercept = F) +
  labs(title = "Coefficient Plot: Hand Release Push Up", x = "Standardized Coefficient", y = "") +
  scale_y_discrete(labels = c("weight" = "Weight","narrowest_waist" = "Narrowest Waist","lower_waist" =
                              "waist_to_hip_ratio" = "Waist to Hip","waist_abdominal" = "Waist Abdominal
                              "lower_thigh" = "Lower Thigh","neck" = "Neck","mid_thigh" = "Mid Thigh","
                              "forearm" = "Forearm","chest" = "Chest","calf" = "Calf","chest_to_waist_ra
                              "chest_to_hip_ratio" = "Chest to Hip","lower_bicep" = "Lower Bicep","bicep
```

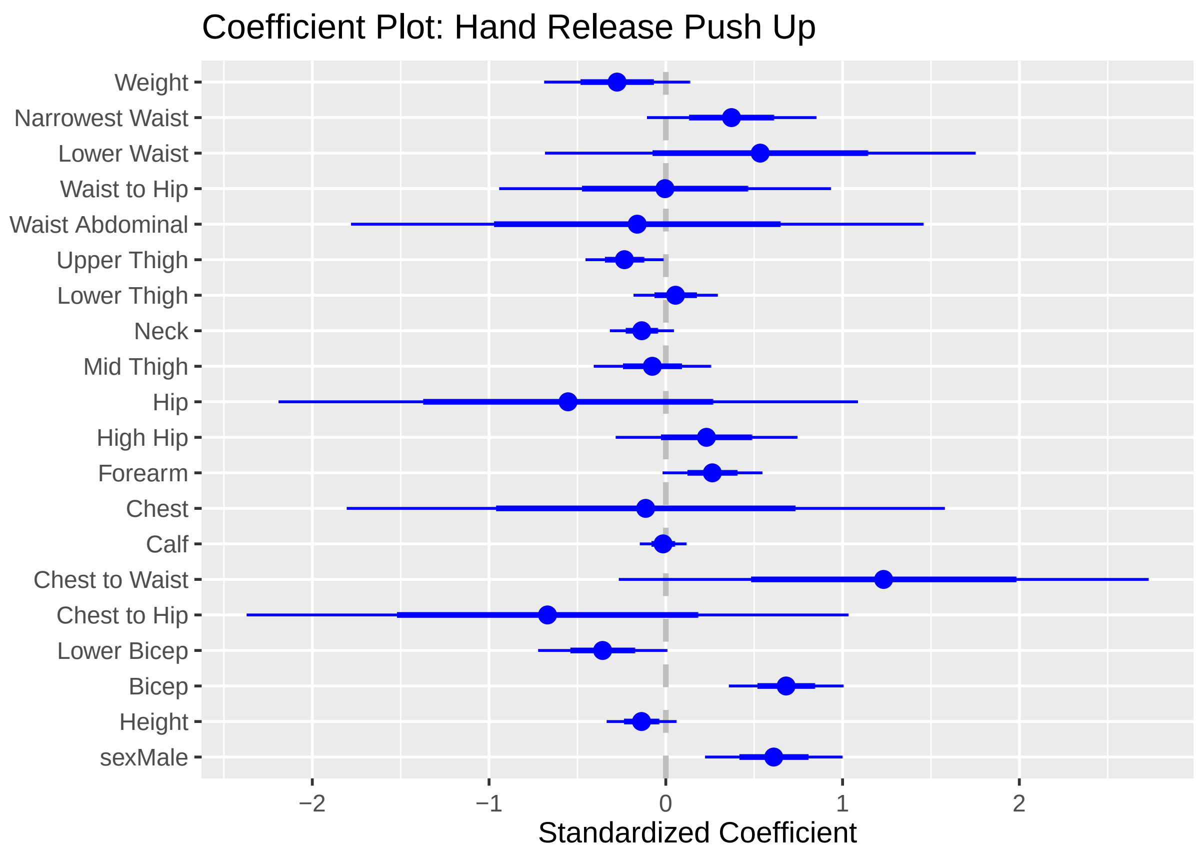

Standing Power Throw

```
mod <-
as.formula(spr~sex + height + bicep + lower_bicep + chest_to_hip_ratio + chest_to_waist_ratio + calf + c
  lm_helper(train = raw_scaled, test = raw_scaled, threshold = .05, model_obj = T)

summary(mod$model_object) %>%
  tidy() %>%
  arrange(p.value) %>%
  print(n = Inf)
```

```
## # A tibble: 21 x 5
##    term                 estimate std.error statistic p.value
##    <chr>                   <dbl>     <dbl>     <dbl>   <dbl>
##  1 sexMale                0.408     0.145      2.82  0.00530
##  2 height                 0.207     0.0734     2.82  0.00531
##  3 (Intercept)           -0.266     0.0995    -2.67  0.00804
##  4 forearm                0.259     0.105      2.47  0.0144
##  5 weight                 0.260     0.154      1.69  0.0923
##  6 chest_to_waist_ratio   0.916     0.559      1.64  0.103
##  7 hip                   -0.807     0.612     -1.32  0.188
##  8 upper_thigh           -0.0991    0.0821    -1.21  0.229
##  9 lower_waist            0.503     0.454      1.11  0.269
## 10 lower_bicep           -0.143     0.136     -1.05  0.295
## 11 calf                  -0.0451    0.0490    -0.921 0.358
## 12 waist_abdominal        0.533     0.604      0.883 0.378
```

```
## 13 chest_to_hip_ratio    -0.517     0.635    -0.813  0.417
## 14 waist_to_hip_ratio    -0.247     0.350    -0.706  0.481
## 15 neck                   0.0473    0.0672    0.704  0.482
## 16 mid_thigh              0.0813    0.123     0.658  0.511
## 17 high_hip               0.125     0.192     0.652  0.515
## 18 lower_thigh            0.0511    0.0886    0.577  0.565
## 19 bicep                  0.0632    0.121     0.524  0.601
## 20 chest                 -0.240     0.631    -0.380  0.704
## 21 narrowest_waist        0.0131    0.178     0.0732 0.942
```

```
summary(mod$model_object) %>% broom::glance()
```

```
## # A tibble: 1 x 8
##   r.squared adj.r.squared sigma statistic  p.value    df df.residual  nobs
##       <dbl>         <dbl> <dbl>     <dbl>    <dbl> <dbl>       <int> <dbl>
## 1     0.788         0.768 0.481      40.5 4.15e-62    20         218   239
```

```
coefplot::coefplot(mod$model_object, intercept = F) +
  labs(title = "Coefficient Plot: Standing Power Throw", x = "Standardized Coefficient", y = "") +
  scale_y_discrete(labels = c("weight" = "Weight","narrowest_waist" = "Narrowest Waist","lower_waist" =
                              "waist_to_hip_ratio" = "Waist to Hip","waist_abdominal" = "Waist Abdomina
                              "lower_thigh" = "Lower Thigh","neck" = "Neck","mid_thigh" = "Mid Thigh","
                              "forearm" = "Forearm","chest" = "Chest","calf" = "Calf","chest_to_waist_r
                              "chest_to_hip_ratio" = "Chest to Hip","lower_bicep" = "Lower Bicep","bice
```

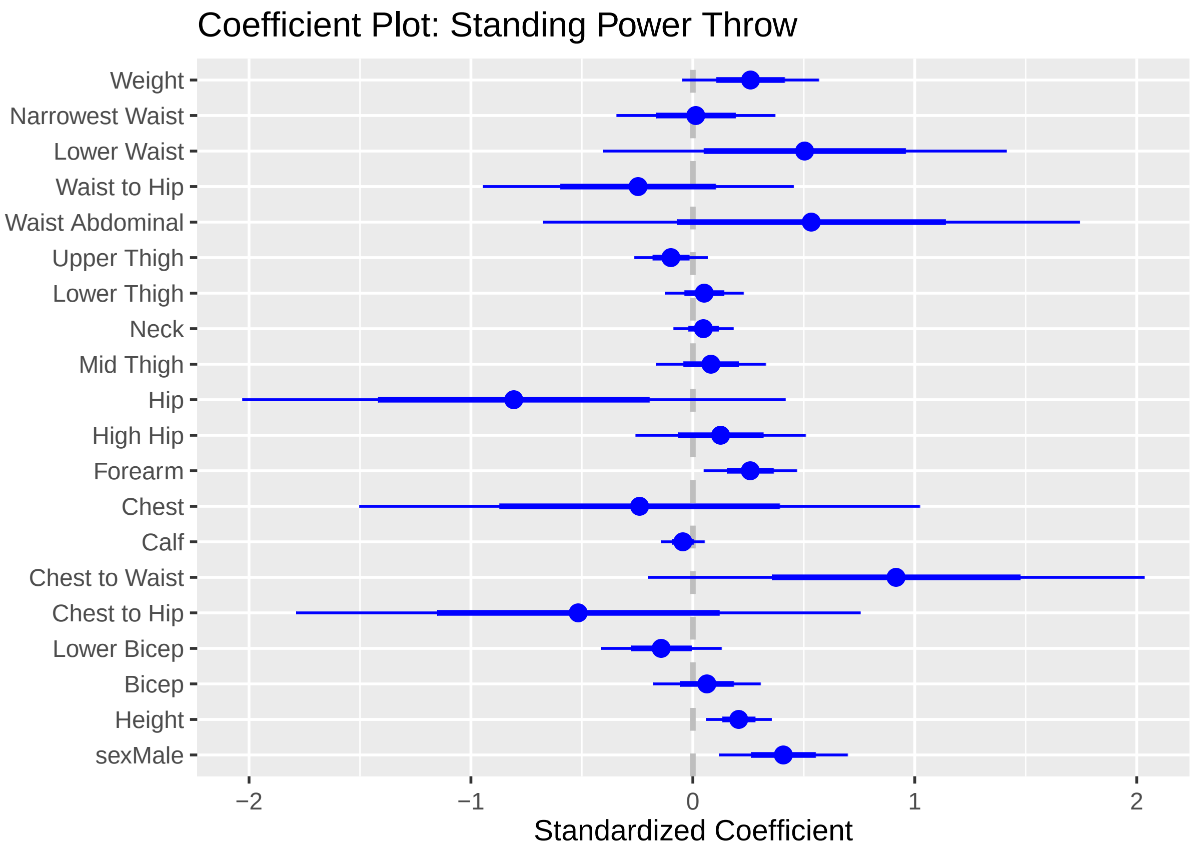

Two Mile Run

```
mod <-
as.formula(mdl~two_mile_run + height + bicep + lower_bicep + chest_to_hip_ratio + chest_to_waist_ratio +
  lm_helper(train = raw_scaled, test = raw_scaled, threshold = .05, model_obj = T)

summary(mod$model_object) %>%
  tidy() %>%
  arrange(p.value) %>%
  print(n = Inf)
```

```
## # A tibble: 21 x 5
##    term                   estimate std.error statistic    p.value
##    <chr>                     <dbl>     <dbl>     <dbl>      <dbl>
##  1 two_mile_run          -1.61e- 1    0.0322 -4.99e+ 0 0.00000121
##  2 narrowest_waist        4.74e- 1    0.140   3.39e+ 0 0.000821
##  3 chest_to_waist_ratio   1.37e+ 0    0.465   2.95e+ 0 0.00349
##  4 bicep                  3.05e- 1    0.104   2.93e+ 0 0.00371
##  5 forearm                2.38e- 1    0.0889  2.67e+ 0 0.00814
##  6 lower_waist            7.45e- 1    0.383   1.94e+ 0 0.0532
##  7 high_hip               2.59e- 1    0.165   1.57e+ 0 0.117
##  8 upper_thigh            1.08e- 1    0.0711  1.52e+ 0 0.130
##  9 hip                   -7.64e- 1    0.526  -1.45e+ 0 0.148
## 10 calf                  -5.46e- 2    0.0421 -1.30e+ 0 0.196
## 11 chest_to_hip_ratio    -6.47e- 1    0.546  -1.18e+ 0 0.238
## 12 lower_bicep           -1.27e- 1    0.117  -1.08e+ 0 0.279
## 13 mid_thigh             -9.31e- 2    0.107  -8.71e- 1 0.385
## 14 chest                 -4.63e- 1    0.532  -8.71e- 1 0.385
## 15 height                 5.40e- 2    0.0624  8.64e- 1 0.388
## 16 weight                 1.02e- 1    0.132   7.75e- 1 0.439
## 17 lower_thigh            3.13e- 2    0.0771  4.06e- 1 0.685
## 18 waist_abdominal       -1.29e- 1    0.518  -2.48e- 1 0.804
## 19 waist_to_hip_ratio    -6.93e- 2    0.301  -2.30e- 1 0.818
## 20 neck                  -6.86e- 3    0.0574 -1.19e- 1 0.905
## 21 (Intercept)           -1.45e-15    0.0268 -5.41e-14 1.00
```

```
summary(mod$model_object) %>% broom::glance()
```

```
## # A tibble: 1 x 8
##   r.squared adj.r.squared sigma statistic  p.value    df df.residual  nobs
##       <dbl>         <dbl> <dbl>     <dbl>    <dbl> <dbl>       <int> <dbl>
## 1     0.843         0.829 0.414      58.7 3.55e-76    20         218   239
```

```
coefplot::coefplot(mod$model_object, intercept = F) +
  labs(title = "Coefficient Plot: Two Mile Run", x = "Standardized Coefficient", y = "") +
  scale_y_discrete(labels = c("weight" = "Weight","narrowest_waist" = "Narrowest Waist","lower_waist" =
                              "waist_to_hip_ratio" = "Waist to Hip","waist_abdominal" = "Waist Abdominal
                              "lower_thigh" = "Lower Thigh","neck" = "Neck","mid_thigh" = "Mid Thigh","
                              "forearm" = "Forearm","chest" = "Chest","calf" = "Calf","chest_to_waist_ra
                              "chest_to_hip_ratio" = "Chest to Hip","lower_bicep" = "Lower Bicep","bicep
```

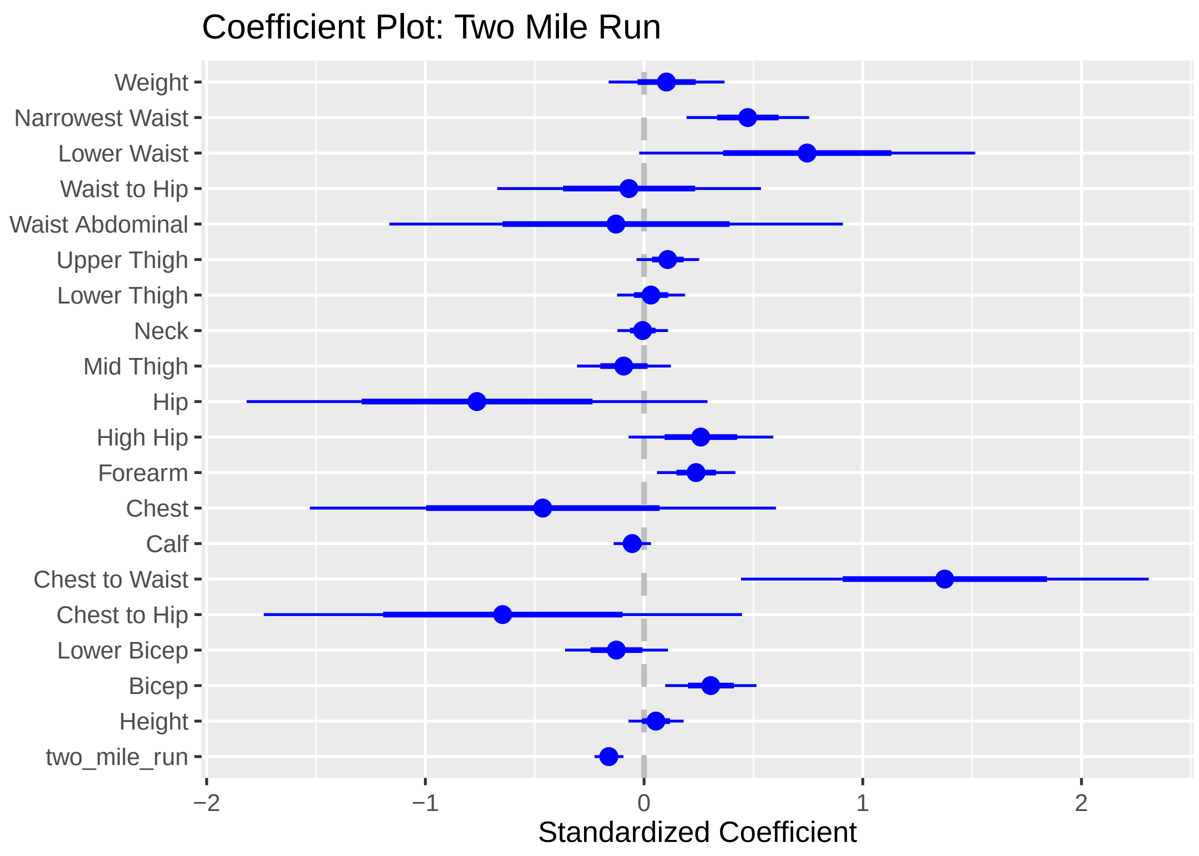
Coefficient Plot: Two Mile Run
Weight
Narrowest Waist
Lower Waist
Waist to Hip
Waist Abdominal
Upper Thigh
Lower Thigh
Neck
Mid Thigh
Hip
High Hip
Forearm
Chest
Calf
Chest to Waist
Chest to Hip
Lower Bicep
Bicep
Height
two_mile_run
−2
−1
0
1
2
Standardized Coefficient
