# Supplementary material for: Body shape and performance on the US Army Combat Fitness Test: Insights from a 3D body image scanner
Source: PLoS One. 2023 May 3;18(5):e0283566. doi: 10.1371/journal.pone.0283566 (PMC10155989; doi:10.1371/journal.pone.0283566)
Supplement: Attachment — Submitted filename: PONE-D-22-21891_RevComments.pdf [file pone.0283566.s008.pdf]

PLOS ONE

Body shape, body composition, and performance on the US Army Combat Fitness Test: Insights from a 3D body image scanner

--Manuscript Draft--

**Manuscript Number:**	PONE-D-22-21891
**Article Type:**	Research Article
**Full Title:**	Body shape, body composition, and performance on the US Army Combat Fitness Test: Insights from a 3D body image scanner
**Short Title:**	Body shape and performance on the US Army Combat Fitness Test
**Corresponding Author:**	Diana M. Thomas<br>United States Military Academy at West Point<br>West Point, NY UNITED STATES
**Keywords:**	Body Composition; Body Shape, 3D Scanner, US Army Combat Fitness Test
**Abstract:**	Objective: To identify relationships between body shape, body composition, sex and performance on the new US Army Combat Fitness Test (ACFT).<br>Methods: Two hundred and thirty-nine United States Military Academy cadets took the ACFT between February and April of 2021. The cadets were imaged with a Styku 3D scanner that measured circumferences at 20 locations on the body, and a subsample of 47 cadets also received dual-energy X-ray absorptiometry body composition measurements. A k-means cluster analysis was performed over the circumference data and differences in body composition and ACFT performance were evaluated between clusters using t-tests with a Holm-Bonferroni correction.<br>Results: The cluster analysis resulted in 5 groups: 1. “V” shaped males, 2. larger males, 3. inverted “V” shaped males and females, 4. “V” shaped smaller males and females, and 5. smallest males and females. ACFT performance was the highest in Clusters 1 and 2 on all events except the 2-mile run. Clusters 3 and 4 had no statistically significant differences in performance but both clusters performed better than Cluster 5.<br>Conclusions: The association between ACFT performance and body shape is more detailed and informative than considering performance solely by sex (males and females) or percent body fat. Some specific anatomical circumferences have higher correlations have higher correlations to performance than body composition. These associations may provide novel ways to design training programs from baseline shape measurements.
**Order of Authors:**	Maria Smith, MS
	Nicholas Gist, Ph.D.
	Sarah Ferreira
	Kevin Quigley, MS
	Charlotte Spencer
	Tyson Walsh
	Nicholas Clark
	William Boldt
	Justin Espe
	Diana M. Thomas
**Additional Information:**	
**Question**	**Response**
**Financial Disclosure**<br><br>Enter a financial disclosure statement that	The senior author Diana M. Thomas was supported by NIH U54TR004279

Powered by Editorial Manager® and ProduXion Manager® from Aries Systems Corporation

describes the sources of funding for the work included in this submission. Review the submission guidelines for detailed requirements. View published research articles from *PLOS ONE* for specific examples.<br><br>This statement is required for submission and **will appear in the published article** if the submission is accepted. Please make sure it is accurate.<br><br>**Unfunded studies**<br>Enter: *The author(s) received no specific funding for this work.*<br><br>**Funded studies**<br>Enter a statement with the following details:<br>• Initials of the authors who received each award<br>• Grant numbers awarded to each author<br>• The full name of each funder<br>• URL of each funder website<br>• Did the sponsors or funders play any role in the study design, data collection and analysis, decision to publish, or preparation of the manuscript?<br>• **NO** - Include this sentence at the end of your statement: *The funders had no role in study design, data collection and analysis, decision to publish, or preparation of the manuscript.*<br>• **YES** - Specify the role(s) played.<br><br>* typeset	
**Competing Interests**<br><br>Use the instructions below to enter a competing interest statement for this submission. On behalf of all authors, disclose any competing interests that could be perceived to bias this work—acknowledging all financial support and any other relevant financial or non-financial competing interests.<br><br>This statement is required for submission and **will appear in the published article** if the submission is accepted. Please make sure it is accurate and that any funding	No

sources listed in your Funding Information later in the submission form are also declared in your Financial Disclosure statement.<br><br>View published research articles from *PLOS ONE* for specific examples.<br><br>**NO authors have competing interests**<br><br>Enter: *The authors have declared that no competing interests exist.*<br><br>**Authors with competing interests**<br><br>Enter competing interest details beginning with this statement:<br><br>*I have read the journal's policy and the authors of this manuscript have the following competing interests: [insert competing interests here]*<br><br>* typeset	
**Ethics Statement**<br><br>Enter an ethics statement for this submission. This statement is required if the study involved:<br><br>• Human participants<br>• Human specimens or tissue<br>• Vertebrate animals or cephalopods<br>• Vertebrate embryos or tissues<br>• Field research<br><br>Write "N/A" if the submission does not require an ethics statement.<br><br>General guidance is provided below. Consult the submission guidelines for detailed instructions. **Make sure that all information entered here is included in the Methods section of the manuscript.**	The study (Number CA20-009) was approved by the United States Military Academy Institutional Review Board and written informed consent was obtained from all study participants.

**Format for specific study types**<br><br>**Human Subject Research (involving human participants and/or tissue)**<br>• Give the name of the institutional review board or ethics committee that approved the study<br>• Include the approval number and/or a statement indicating approval of this research<br>• Indicate the form of consent obtained (written/oral) or the reason that consent was not obtained (e.g. the data were analyzed anonymously)<br><br>**Animal Research (involving vertebrate animals, embryos or tissues)**<br>• Provide the name of the Institutional Animal Care and Use Committee (IACUC) or other relevant ethics board that reviewed the study protocol, and indicate whether they approved this research or granted a formal waiver of ethical approval<br>• Include an approval number if one was obtained<br>• If the study involved *non-human primates*, add *additional details* about animal welfare and steps taken to ameliorate suffering<br>• If anesthesia, euthanasia, or any kind of animal sacrifice is part of the study, include briefly which substances and/or methods were applied<br><br>**Field Research**<br><br>Include the following details if this study involves the collection of plant, animal, or other materials from a natural setting:<br>• Field permit number<br>• Name of the institution or relevant body that granted permission	
**Data Availability**<br><br>Authors are required to make all data underlying the findings described fully available, without restriction, and from the time of publication. PLOS allows rare exceptions to address legal and ethical concerns. See the PLOS Data Policy and FAQ for detailed information.	No - some restrictions will apply

Powered by Editorial Manager® and ProduXion Manager® from Aries Systems Corporation

A Data Availability Statement describing where the data can be found is required at submission. Your answers to this question constitute the Data Availability Statement and **will be published in the article**, if accepted.<br><br>**Important:** Stating 'data available on request from the author' is not sufficient. If your data are only available upon request, select 'No' for the first question and explain your exceptional situation in the text box.<br><br>Do the authors confirm that all data underlying the findings described in their manuscript are fully available without restriction?	
**Describe where the data may be found in full sentences. If you are copying our sample text, replace any instances of XXX with the appropriate details.**<br><br>• If the data are **held or will be held in a public repository**, include URLs, accession numbers or DOIs. If this information will only be available after acceptance, indicate this by ticking the box below. For example: *All XXX files are available from the XXX database (accession number(s) XXX, XXX.).*<br>• If the data are all contained **within the manuscript and/or Supporting Information files**, enter the following: *All relevant data are within the manuscript and its Supporting Information files.*<br>• If neither of these applies but you are able to provide **details of access elsewhere**, with or without limitations, please do so. For example:<br><br>*Data cannot be shared publicly because of [XXX]. Data are available from the XXX Institutional Data Access / Ethics Committee (contact via XXX) for researchers who meet the criteria for access to confidential data.*<br><br>*The data underlying the results presented in the study are available from (include the name of the third party*	The data will be made available upon request to the United States Military Academy.

and contact information or URL).<br>• This text is appropriate if the data are owned by a third party and authors do not have permission to share the data.<br><br>* typeset	
Additional data availability information:	

Powered by Editorial Manager® and ProduXion Manager® from Aries Systems Corporation

Manuscript

Click here to access/download;Manuscript;Paper Draft Revisions 4-15-2022.docx

Body shape, body composition, and performance on the US Army Combat Fitness Test: Insights from a 3D body image scanner

Maria Smith[1], Dusty Turner[2], Charlotte Spencer[1], Nicholas Gist[3], Sarah Ferreira[3], Kevin Quigley[1], Tyson Walsh[1], Nicholas Clark[1], William Boldt[1], Justin Espe[1], Diana M. Thomas[1]

1Department of Mathematical Sciences, United States Military Academy, West Point, NY

2Center for Army Analysis, Fort Belvoir, VA

3 Department of Physical Education, United States Military Academy, West Point, NY

Address Correspondence to:

Diana M. Thomas, Ph.D.
ORCID ID: 0000-0003-2641-9304
Department of Mathematical Sciences
United States Military Academy
West Point, NY 10996

Tel: 973-224-2562
E-mail: diana.thomas@westpoint.edu

Running title: Performance on the Army Combat Fitness Team

Abbreviations: ACFT, Army Combat Fitness Test; BMI, body mass index; DXA, dual-energy X-ray absorptiometry; FFMI, fat-free mass index; FMI, fat mass index; MDL 3-repitition maximum deadlift; SPT Standing Power Throw; HRPU hand release pushups; SDC sprint drag and carry; LT leg tuck

Word Count: 2,999

Key words: Body Composition; Skeletal Muscle Mass; Adiposity; Resting Energy Expenditure.

Abstract:

Objective: To identify relationships between body shape, body composition, sex and performance on the new US Army Combat Fitness Test (ACFT).

Methods: Two hundred and thirty-nine United States Military Academy cadets took the ACFT between February and April of 2021. The cadets were imaged with a Styku 3D scanner that measured circumferences at 20 locations on the body, and a subsample of 47 cadets also received dual-energy X-ray absorptiometry body composition measurements. A k-means cluster analysis was performed over the circumference data and differences in body composition and ACFT performance were evaluated between clusters using t-tests with a Holm-Bonferroni correction.

Results: The cluster analysis resulted in 5 groups: 1. “V” shaped males, 2. larger males, 3. inverted “V” shaped males and females, 4. “V” shaped smaller males and females, and 5. smallest males and females. ACFT performance was the highest in Clusters 1 and 2 on all events except the 2-mile run. Clusters 3 and 4 had no statistically significant differences in performance but both clusters performed better than Cluster 5.

Conclusions: The association between ACFT performance and body shape is more detailed and informative than considering performance solely by sex (males and females) or percent body fat. Some specific anatomical circumferences have higher correlations have higher correlations to performance than body composition. These associations may provide novel ways to design training programs from baseline shape measurements.

Summary:

What are the new findings?

- There are five different homogeneous groups of body shapes identified from Styku 3D body image measured circumferences.
- Performance on the new ACFT differs between the five groups.
- The association between body circumferences and performance is stronger than between body circumference and percent body fat.
- Three of the five groups are comprised of a combination of males and females.

How might these findings impact clinical practice in the future?

- Training programs for military fitness tests could consider body shape tailored programs.

Competing Interest and Funding: This study was supported by a grant from the Army Research Laboratory (ARL). The authors have no competing interest to declare.

INTRODUCTION

Based on a recent RAND report[1], the United States Army has changed the new Army Combat Fitness Test (ACFT) from an age and sex independent physical assessment to an assessment that is scored normalized by age-and-sex-performance. In addition, one of the ACFT events, the leg tuck, is now replaced with a plank event. Prior to these changes, there were notable differences in performance between males and females on the new United States Army Combat Fitness Test (ACFT)[2-4].

The original version of the ACFT was a six-event physical fitness test[2 5] designed to be sex and age neutral that assesses whether soldiers are able to perform physical tasks required for combat, like casualty evacuation, movement under fire, construct a fighting position[2]. The concept behind age-sex neutrality was because all soldiers must accomplish these tasks, predicting performance from easily measurable individual factors and understanding what specific role sex plays is important for designing effective training programs.

Body size is one explanation for differences in performance between the sexes[6]. The body mass index (BMI) is routinely collected in Army soldiers[6 7]. Higher BMI is associated with better performance on events requiring muscular strength and power such as medicine ball throws, sled drags, and dead lifts[8 9]. On the other hand, higher BMI is inversely related to aerobic events such as the 2-mile run[8 9]. BMI does not equate to body fat, however, it is known that high fat mass index (FMI) is associated with high fat free mass index (FFMI)[10], which in turn is associated with better performance on muscular strength activities. BMI also does not provide body shape insights which have been known to correlate to events such as pull-ups[11 12].

We extended this body of existing work using a discovery science approach by improving understanding of body shape and performance beyond BMI by recruiting 239 United States Military Academy (USMA) cadets who took the ACFT ~~between February and May 2021~~ and pairing their ACFT performance to body shape and body composition measurements.

METHODS

Study design

This was a cross-sectional observational study of 239 USMA cadets who took the ACFT between February and May of 2021. Relationships between 3D imaged body circumferences, DXA measured body composition, and ACFT performance was examined.

While BMI serves as one proxy for body shape, it is desirable to collect more markers of shape through anthropometric measurements[13]. However, manually collecting body circumferences can represent a burden to both researcher and participant. Therefore, we used a new technology that obtains measurements quickly and efficiently for analysis. The cadets were scanned using 3D imaging technology which calculated anatomical circumferences at 20 locations on the body. Body composition was also collected with dual energy X-ray

absorptiometry (DXA) in a subsample of cadets. These measurements were used to identify different body shape and body composition attributes related to performance on the specific ACFT events.

Study cohort

Participants were recruited through posters, announcements in the dining facility, and emails disseminated by cadet strength and conditioning staff. Participants were eligible if they were currently USMA cadets who had taken or were going to take the ACFT between February and May of 2021. USMA cadets are between the ages of 18 to 29 years old. The study (Number CA20-009) was approved by the USMA Institutional Review Board and written informed consent was obtained from all study participants.

The Army Combat Fitness Test (ACFT)

The six ACFT events[14] (Online **Supplemental File 1**), conducted in order with a maximum of 2 minutes between events, include a 3-Repetition Maximum Deadlift (MDL), Standing Power Throw (SPT), Hand-Release Push-Ups (HRPU), Sprint-Drag-Carry (SDC), Leg Tuck (LT), and the 2-mile run[3].

The ACFT was taken by participants between the end of February 2021 and the beginning of May 2021, and scores were maintained by the USMA Office of Institutional Research office. After participants were scanned, their raw scores on the ACFT were obtained from institutional research.

3D body image scan measurements

Between February and March of 2021, participants arrived at a designated location to be scanned using the Styku 3D body imaging machine (Styku S100, Advanced Model of Styku Phoenix software; Los Angeles, CA). Participants stood on a rotating turntable with their arms

held in an "A" pose. As the turntable revolved for 35 seconds, a tower 114 cm away captured images using an infrared camera equipped with "Time of Flight" technology that quantifies the depth in an image[15]. Male participants wore their USMA issued swimsuit or compression shorts and female participants wore their USMA issued swimsuit or compression shorts and a sports bra. The Styku internal software outputs body circumference measurements at 20 different locations, ratios of chest-to-waist, chest-to-hip and waist-to-hip, and body weight. All statistical analysis used averaged left and right limb circumferences (**Figure 1**), which reduced to 13 averaged circumferences.

Body Composition

Body composition was measured by DXA (General Electric Healthcare, Lunar Prodigy Advance, Madison, WI) in 47 participants who were already scanned with the Styku. Participants recruited for DXA scans reported to the human performance laboratory and were scanned by the same investigator ~~during the months~~ between April and May 2021.

Statistical Methods

Correlation between body site measurements, percent body fat, and ACFT event performance

To evaluate how individual body circumference measured by the Styku are associated with ACFT event-specific performance, we generated correlation plots, Pearson correlation coefficients, and p-values for each event score against each of the 13 circumferences, chest-to-waist ratio, chest-to-hip ratio, waist-to hip ratio, BMI, weight, height, and percent body fat versus ACFT event-specific scores.

Regression models predicting ACFT event specific performance

Six different linear regression models were developed using all circumferences and ratios as independent variables and event performance as the dependent variable. Model coefficients in

the final models were standardized and plotted with 95% confidence intervals to compare the contribution of each measured body site in the final model on performance.

k-means Cluster Analysis

To group the participant population by similar body shapes, a k-means cluster analysis was performed over the normalized body shape measurements (ratio of the difference of measurement and mean and standard deviation) of the 13 circumference sites, body weight, and body height. As noted earlier, clustering is an unsupervised method which allows the data to define groupings and eliminates human preconceived groupings of populations (e.g. by sex). The number of clusters to retain was determined by the cluster number at the elbow of the scree plot. A heat map was generated by cluster identifying how far the body site locations were from the sample mean value.

Comparison of percent body fat and ACFT performance between clusters

To identify the role of the outcomes of percent body fat and ACFT performance across clusters, pairwise t-tests were performed with a Holm-Bonferroni correction[16] to account for multiple comparisons.

All statistical analyses were performed in the software package R (**R** Core Team (2013)).

RESULTS

Participant Characteristics

A total of 265 participants were enrolled in the study and scanned using the Styku. Of these, 239 completed the ACFT. In the reference dataset of 239 participants with both Styku measurements and ACFT scores, 47 were measured with DXA. In the reference dataset, there were 83 females and 156 males. **Table 1** contains a summary of the data by sex, cluster number, and aggregate totals. Included are BMI (kg/m^2), bicep (Label A **Figure 1**) , waist (Label I **Figure**

1), thigh (Label F **Figure 1**), chest (Label H **Figure 1**), and forearm (Label C **Figure 1**) circumference. Females had lower BMI (22.36 ±2.22 m/kg^2) than males (25.33±2.99 m/kg^2) and smaller average circumferences than males.

Cluster Analysis

Based the number of clusters at the elbow of the scree plot (Online **Supplemental File 2**), five clusters were retained for analysis. A cluster heat map appears in **Figure 2** with darker shades of green representing measurements further below the mean and darker shades of red representing measurements higher than the mean. Cluster 1 consisted of primarily "V-shaped" males (only 1 female) with chest-to-waist ratio over 1. Cluster 2 contained only males that had the highest BMI and waist circumference over all clusters. Cluster 2 had a chest-to-waist ratio of 1.14 ± 0.6 while Cluster 1 had a chest-to-waist ration of 1.19 ± 0.06. The third cluster contains a combination of both males and females. The chest-to-hip ratio is 0.86 ± 0.4 yielding an average body shape in this cluster that is an inverted "V". Cluster 4 consists of a majority male population that were "V" shaped and had smaller than average body circumferences (**Figure 2**). Finally, Cluster 5 consists of primarily females with body circumferences below average and inverted "V" shapes. The Cluster 5 population had lower BMI (21.00 ±1.30) compared to Cluster 3 (24.13 ±1.93).

Body shape and ACFT Performance

ACFT performance by cluster

Overall average performance on every ACFT event was highest in Cluster 1 on every event except the SPT (**Table 1, Box Plots Online Supplemental File 3**). Participants classified to Cluster 2 had the highest performance on the SPT event and second highest performance on the MDL and SDC events. Individuals in this cluster had similar performance to Cluster 4 on the

HRPU event and performed below Cluster 4 on the LT and the 2-mile run. Participants in Cluster 3 performed above Cluster 5 on all events except the 2-mile run.

Comparison of means between clusters revealed that Clusters 1 and 2 had statistically significant better performance than Clusters 3, 4 and 5 for the MDL (Online **Supplemental File 4**). Additionally, Clusters 3 and 4 had significantly better performance on the MDL compared to Cluster 5. For SPT, HRPU and SDC and LT, Clusters 1 and 2 had statistically significant better performance than Clusters 3 and 5. Finally, Cluster 4 had statistically significant better performance on the SDC and LT compared to Cluster 5. There were no statistically significant differences in performance over any ACFT event between Cluster 3 and Cluster 4.

Correlation of Body Circumference and ACFT performance

Table 2 contains the Pearson correlation coefficients between the measurements provided by the Styku against each ACFT event. The highest correlations over all events were held by bicep circumference (Label A **Figure 1**), chest-to-waist ratio, and forearm circumference (Label E **Figure 1**). The lowest correlations were observed in hip circumference, waist circumference, thigh circumference, and BMI.

BMI and circumferences sites at the waist and hip did not have statistically significant correlations to the 2-mile run (Online **Supplemental File 5)**.

Regression models predicting ACFT event specific performance

Table 3 contains statistically significant model terms and the adjusted R^2 of models predicting ACFT event-specific scores body measurements (for full model details see Online **Supplemental File 6)**. Bicep circumference (Label A **Figure 1**) appeared in the MDL, HRPU, and LT with coefficients indicating larger bicep circumferences was associated with better scores on these events. Height appeared as a statistically significant term in the SPR, SDC, and 2-mile

run models with taller individuals associated with better scores on the SPR and 2-mile run, and lower scores on the SDC. Males were positively related to performance for all events except the 2-mile run. **Figure 3** depicts the standardized coefficients and 95% confidence intervals for model terms. Larger lower bicep circumference (Label B **Figure 1**) was related to lower performance on all events while, higher chest-to-waist ratio was associated to positive performance on all events. Larger forearm circumference (Label C **Figure 1**) was associated to positive performance on all events except the 2-mile run.

Body Composition

Percent Body Fat by Cluster

Clusters with higher percent females had highest percent body fat (**Table 1**) and Cluster 3 has the highest mean percent body fat among the clusters, while Cluster 4 has the lowest mean percent body fat among the clusters (**Table 1**).

Percent body fat was statistically different in Clusters 1 and 3, Clusters 1 and 5, Clusters 2 and 4, Clusters 3 and 4, and Clusters 4 and 5, however after multiple comparison corrections, statistically significant differences in percent body fat remained between Clusters 1 and 3 ($p=0.0004$), Clusters 1 and 5 ($p=0.0012$) and Clusters 3 and 4 ($p=0.0031$) (Online **Supplemental File 4**).

Correlation of Body Composition and ACFT performance

Percent body fat was significantly correlated ($p<0.001$) to performance on all events (**Table 2**). Percent body fat was inversely correlated to better performance on all events with the highest association on the HRPU ($r=-0.65$), SDC ($r=0.67$), and LT ($r=-0.65$) (Online **Supplemental File 7)**.

Correlation of Body Composition and Body Shape

Body circumferences with highest correlation to percent fat were waist related circumferences (Online **Supplemental File 7)**. Female body circumferences had consistently higher correlations which were significant with percent body fat. For example, for females, the association between lower bicep circumference ($r=0.70$, $p<0.01$), forearm circumference (0.74, $p<0.001$), and lower waist ($r=0.70$, $p<0.01$) with percent body fat was stronger than male lower bicep circumference (-0.006, not statistically significant), forearm circumference (-0.08, not statistically significant), and lower waist (0.49, $p<0.01$).

DISCUSSION

There are differences between male and female performance on military fitness tests[4 17-20], however, grouping performance data solely by sex places males and females into distinct clusters with no overlap. Here we used a clustering algorithm that grouped 239 cadets at the United States Military Academy into five clusters of similar body shapes. Of these five groups, three resulted in a combination of males and females. While the top performing clusters were predominately male, the next performance rank was held by two clusters with a combination of males and females.

Existing studies in the US Army predicting physical performance largely focused on relationships to body composition[21-23] to the phased out Army Physical Fitness Test (APFT). Because the ACFT only became the US Army physical assessment of record in October 2020[24], there is only one existing study on factors that predict performance. A recent study in 68 U.S. Army Reserve Officer Training Corps (ROTC) cadets measured body composition using bioimpedance and examined relationships between body composition and ACFT performance[17]. The study[17] found that there were significant correlations between fat free mass index (FFMI) and fat free mass index (FFMI) with overall ACFT performance. These findings are synergistic

with our cluster analysis results in Cluster 1 outperforming other archetype shapes on the ACFT. The study[17] did not find correlations between BMI and overall ACFT scores, which is in contrast to our results in **Table 2**. We found that BMI was correlated to positive performance on all ACFT events except the 2-mile run.

The ACFT represents a combination of events that require muscular strength, power and anaerobic endurance (MDL, SPT, SDC) and aerobic endurance (2-mile run). To our knowledge, the role of body composition on the ACFT events like HRPU and LT have not been reported, however several studies have found that higher percent body fat is correlated to lower push-up and pull-up performance[11 12 25 26]. Our finding that higher percent body fat was associated with lower HRPU and LT performance corroborate and extend the existing findings to ACFT events.

The analysis here using 3D imaged body circumference also extends the literature by identifying key body circumferences that are associated with performance. Larger forearm and bicep circumference were associated with better performance on all events excluding the 2-mile run. On the other hand, larger lower bicep and mid-thigh circumferences were associated with lower performance on the HRPU, LT and 2-mile run. Because the circumference at the lower bicep in females was significantly correlated to percent body fat with $r=0.70$, $p<0.001$, the lower bicep may be a proxy for body fat in females.

Sex-specific differences in body shape, composition, and ACFT performance

It is well known that athletic performance differs between males and females[27-29] and that this difference is physiological. For example, females have lower body and muscle mass and strength, lower bone mass, and lower oxygen-transport capacity than males[30-32] and females in general have higher percent body fat and FMI than males[10 33]. These findings were concordant with our study, because within each cluster, males in general outperformed females on the ACFT

events and the clusters with majority females (Cluster 3 and 5) had the highest average percent body fat. Moreover, larger bicep and forearm circumference were identified as important factors predicting performance on all events except the 2-mile run. The largest bicep and forearm circumferences were exhibited by Clusters 1 and 2 which were predominately male (only 1 female). However, previous research on trained body builders found that female bicep circumferences are lower than males[34 35]. This suggests that even with training, it may not be feasible for females to have comparable bicep circumferences to males.

Despite our concordant findings of sex-differences in performance, we note that Clusters 3 and 4 did not have statistically significant differences in ACFT performance, while both Clusters 3 and 4 did have statistically significant differences in performance compared to Cluster 5. Solely focusing on differences in physical performance between males and females would have obscured this finding. Instead designing training programs to shift Cluster 5 body shapes to to Cluster 3 or 4 may be more encompassing. It remains to be shown if altering body shapes will improve performance.

Study Strengths and Limitations

The use of the Styku 3D scanner, was a major strength of the study. In addition, the carefully curated routine collection and storage of physical performance data at USMA allowed us to examine relationships between body shape and ACFT performance.

On the other hand, USMA cadets are not representative of the larger Army or the US population. In addition, the ACFT was not incorporated into USMA grades and cadets may not have been performing at maximal ability. However, our convenience sample may have self-selected motivated cadets.

There are several statistical limitations of our study. Human body proportions and circumferences have known relationships previously identified through allometric modeling[36 37]. There may be implausible combinations of body circumferences that do not obey these relationships reflected by the regression models. Despite this, the standardized model coefficients provided insights that were congruent with the literature. Moreover, the cluster analysis was performed so that each participant's circumferences were one data array in the cluster analysis.

Finally, the identified relationships between body shape and performance is correlation not causation. Longitudinal evidence is needed to determine whether changed body shape will lead to changes in physical performance on the ACFT. Moreover, with the new sex and age-specific scoring system and the replacement of the leg tuck event with a plank[1], some of our findings do not transfer to the modified ACFT.

Research on predicting performance has relied on body composition to predict performance[21]. Measurements from 3D body image scanners provide a new wealth of more detailed information on body shape that advances our understanding of which body types are associated with better performance. The scanners are affordable, do not require a special license to operate, provide measurements within seconds, and do not expose participants to radiation. Thus, they offer a feasible method to identify individuals and enroll them into targeted training programs.

Conclusions

Body shape predicts performance on the new ACFT better than body composition alone, as shown through the previously mentioned models and correlations. Archetype body shape

groups are associated with different levels of physical performance, providing new opportunities for targeted training that go beyond grouping populations by sex.

Contributions

Nicholas Clark, Andrew Inman, and Diana M. Thomas conceived this study. Charlotte Spencer, Maria Smith, Sarah Ferreira, Kevin Quigley, and Tyson Walsh performed Styku body scans. Maria Smith, Charlotte Spencer, Dusty Turner, Tyson Walsh, and Diana M. Thomas performed the statistical analysis. Nicholas Gist performed the dual energy X-ray absorptiometry scans. Maria Smith, Charlotte Spencer, and Diana M. Thomas wrote the first draft of the manuscript. All authors reviewed multiple versions of the manuscript.

Data Sharing Statement: Individual deidentified participant data used in this study (including data dictionaries) will be shared upon approval by the G5 Office of Institutional Research at the United States Military Academy. Statistical analyses that were performed are available as R Markdown files in the supplementary online information (**Online Supplemental File 6**).

Acknowledgements: The authors thank the following colleagues for reading and commenting on the final drafts of the manuscript prior to submission: Leslie Jones, Blake Schwartz, and Daniel Baller.

Financial Disclosure: Nicholas Clark, Diana M. Thomas and Nicholas Gist are supported by NIH 1U54TR004279

REFERENCES

1. Hardison CM, Mayberry PW, Krull H, et al. Independent Review of the Army Combat Fitness Test: Summary of Key Findings and Recommendations. Santa Monica, CA: RAND Corporation 2022.

2. Holistic Health and Fitness. In: Army, ed. Headquarters, Department of the Army, Washington, D.C.: United States Army, 2020.

3. Taguchi N. Developing interactional competence in a Japanese study abroad context. Bristol ; Buffalo: Multilingual Matters 2015.

4. Brading T. ACFT 3.0: Exploring a more inclusive scoring assessment, planks stay 2021 [Available from: https://www.army.mil/article/244220/acft_3_0_exploring_a_more_inclusive_scoring_assessment_planks_stay accessed June 10, 2021 2021.

5. Holistic Health and Fitness Testing. In: Army, ed. Washington D.C.: HEADQUARTERS, DEPARTMENT OF THE ARMY, 2020.

6. Nelson R, Cheatham J, Gallagher D, et al. Revisiting the United States Army body composition standards: a receiver operating characteristic analysis. *Int J Obes (Lond)* 2019;43(8):1508-15. doi: 10.1038/s41366-018-0195-x [published Online First: 2018/09/06]

7. Army Regulation 600-9 The Army Body Composition Program. In: DoD, ed. Washington DC: The Department of the Army, 2013.

8. Pierce J, Hauret K, Alemany J, et al. Physical Performance on the Occupational Physical Assessment Test (OPAT), Army Physical Fitness Test (APFT), and Relationship to Body Mass Index During Initial Entry Training - OPAT Phase 12018.

9. Pierce JR, DeGroot DW, Grier TL, et al. Body mass index predicts selected physical fitness attributes but is not associated with performance on military relevant tasks in U.S. Army Soldiers. *Journal of Science and Medicine in Sport* 2017;20:S79-S84. doi: https://doi.org/10.1016/j.jsams.2017.08.021

10. Thomas D, Das SK, Levine JA, et al. New fat free mass - fat mass model for use in physiological energy balance equations. *Nutr Metab (Lond)* 2010;7:39. doi: 10.1186/1743-7075-7-39 [published Online First: 2010/05/13]

11. Sanchez-Moreno M, Pareja-Blanco F, Diaz-Cueli D, et al. Determinant factors of pull-up performance in trained athletes. *J Sports Med Phys Fitness* 2016;56(7-8):825-33. [published Online First: 2015/07/16]

12. Johnson D, Lynch J, Nash K, et al. Relationship of lat-pull repetitions and pull-ups to maximal lat-pull and pull-up strength in men and women. *J Strength Cond Res* 2009;23(3):1022-8. doi: 10.1519/JSC.0b013e3181a2d7f5 [published Online First: 2009/04/24]

13. Vogel JA, Kirkpatrick JW, Fitzgerald PI, et al. Derivation of Anthropometry Based Body Fat Equations for the Army's Weight Control Program. In: MEDICINE ARIOE, ed. Natick, MA, 1988.

14. Army Combat Fitness Test Fort Eustis, Virginia [Available from: https://www.army.mil/acft/ accessed 05 SEPT 21 2021.

15. Sobhiyeh S, Dunkel A, Dechenaud M, et al. Digital Anthropometric Volumes: Toward the Development and Validation of a Universal Software. *Medical physics (Lancaster)* doi: 10.1002/mp.14829

16. Holm S. A Simple Sequentially Rejective Multiple Test Procedure. *Scandinavian Journal of Statistics* 1979;6(2):65-70.

17. Roberts BM, Rushing KA, Plaisance EP. Sex Differences in Body Composition and Fitness Scores in Military Reserve Officers' Training Corps Cadets. *Mil Med* 2021 doi: 10.1093/milmed/usaa496 [published Online First: 2021/01/16]

18. Steinhauer J. Where Fitness Is the Job, Army Struggles to Be a Fair Boss With Female Troops. *New York Times* 2021 03/11/

2021 Mar 11.

19. Teyhen DS, Shaffer SW, Goffar SL, et al. Identification of Risk Factors Prospectively Associated With Musculoskeletal Injury in a Warrior Athlete Population. *Sports Health* 2020;12(6):564-72. doi: 10.1177/1941738120902991

20. Johnson QR, Scraper J, Lockie R, et al. Sex-related Differences in Functional Movement Screen Scores Among Reserve Officers' Training Corps Cadets. *Mil Med* 2021 doi: 10.1093/milmed/usaa417 [published Online First: 2021/03/27]

21. Friedl KE, Leu JR. Body fat standards and individual physical readiness in a randomized Army sample: screening weights, methods of fat assessment, and linkage to physical fitness. *Mil Med* 2002;167(12):994-1000. [published Online First: 2002/12/28]

22. Friedl KE. Body composition and military performance--many things to many people. *J Strength Cond Res* 2012;26 Suppl 2:S87-100. doi: 10.1519/JSC.0b013e31825ced6c [published Online First: 2012/05/31]

23. Body composition and physical performance : applications for the military services. In: Marriott BM, Grumstrup-Scott J, eds. Washington, DC :: National Academy Press, 1992.

24. Army Combat Fitness Test Set to Become Test of Record. Washington: Federal Information & News Dispatch, LLC, 2018.

25. Steed CLMFNRD, Krull BRMFNRD, Morgan ALP, et al. Relationship Between Body Fat and Physical Fitness in Army ROTC Cadets. *Military Medicine* 2016;181(9):1007-12. doi: http://dx.doi.org/10.7205/MILMED-D-15-00425

26. Merrigan JJ, Burke AA, Fyock-Martin MB, et al. What Factors Predict Upper Body Push to Pull Ratios in Professional Firefighters? *International journal of exercise science* 2020;13(4):1605-14.

27. Fuchs PX, Menzel H-JK, Guidotti F, et al. Spike jump biomechanics in male versus female elite volleyball players. *Journal of sports sciences* 2019;37(21):2411-19. doi: 10.1080/02640414.2019.1639437

28. Beneke R, Leithäuser RM. Gender, Sex, Sex Differences, Doping in Athletic Performance. *International journal of sports physiology and performance*;14(7):869-70. doi: 10.1123/ijspp.2019-0483

29. Handelsman DJ, Hirschberg AL, Bermon S. Circulating Testosterone as the Hormonal Basis of Sex Differences in Athletic Performance. *Endocrine Reviews* 2018;39:803+.

30. Heo M, Faith MS, Pietrobelli A, et al. Percentage of body fat cutoffs by sex, age, and race-ethnicity in the US adult population from NHANES 1999–2004. *The American Journal*

of Clinical Nutrition 2012;95(3):594-602. doi: 10.3945/ajcn.111.025171 %J The American Journal of Clinical Nutrition

31. Reybrouck T, Fagard R. Gender differences in the oxygen transport system during maximal exercise in hypertensive subjects. *Chest* 1999;115(3):788-92. doi: 10.1378/chest.115.3.788 [published Online First: 1999/03/20]
32. LoMauro A, Aliverti A. Sex differences in respiratory function. *Breathe (Sheffield, England)* 2018;14(2):131-40. doi: 10.1183/20734735.000318 [published Online First: 2018/06/08]
33. Borrud LG, Flegal KM, Looker AC, et al. Body composition data for individuals 8 years of age and older: U.S. population, 1999-2004. *Vital and health statistics Series 11, Data from the National Health Survey* 2010(250):1-87. [published Online First: 2010/09/04]
34. Alway SE, Grumbt WH, Gonyea WJ, et al. Contrasts in muscle and myofibers of elite male and female bodybuilders. 1989;67(1):24-31. doi: 10.1152/jappl.1989.67.1.24
35. Fry AC, Ryan AJ, Schwab RJ, et al. Anthropometric characteristics as discriminators of body- building success. *Journal of sports sciences* 1991;9(1):23-32. doi: 10.1080/02640419108729852
36. Watts K, Hwaung P, Grymes J, et al. Allometric models of adult regional body lengths and circumferences to height: Insights from a three-dimensional body image scanner. *American journal of human biology : the official journal of the Human Biology Council* 2020;32(3):e23349. doi: 10.1002/ajhb.23349 [published Online First: 2019/10/28]
37. Hwaung P, Heo M, Kennedy S, et al. Optimum waist circumference-height indices for evaluating adult adiposity: An analytic review. *Obes Rev* 2020;21(1):e12947. doi: 10.1111/obr.12947 [published Online First: 2019/09/12]

Table 1: Table contains mean (±SD) BMI and Styku body circumferences at five different sites by cluster, sex and totals. Waist circumference was the Styku measurement at the abdomen (labeled "I" in Figure 1) and thigh circumference was the Styku measurement at the lower thigh (labeled "F" in Figure 1). Mean (±SD) of the raw scores for each ACFT event are also provided. Finally, mean (±SD) of percent body fat in 47 participants are presented by cluster with sample sizes: Cluster 1 (13) Cluster 2 (8), Cluster 3 (10), Cluster 4 (5), and Cluster 5 (11).

[*]Body composition was collected in 47 of the participants with 40% females.

*Abbreviations are N – number of participants, %F – percent female, BMI – body mass index, MDL – maximum deadlift, SPT – standing power throw, HRPU – hand release push-up, SDC – sprint-drag-carry, LT – leg tuck.

Cluster Number & Group	1	2	3	4	5	Female	Male	Total
**N**	70	27	50	39	53	83	156	239
**%F**	1	0	64	8	89	---	---	35
**BMI (kg/m^2)**	26.10±1.73	29.31±1.66	24.13±1.93	22.30±1.44	21.00±1.30	22.36±2.22	25.33±2.99	24.30±3.09
**%Fat***	15.4±5.02	22.0±7.84	27.6±5.71	12.7±6.47	22.4±4.15	25.5±5.64	17.35±6.76	20.5±7.47
**Bicep (cm)**	32.65±1.92	34.95±2.24	28.16±2.16	28.67±2.02	24.37±1.51	25.60±2.18	31.55±3.20	29.48±4.05
**Waist (cm)**	78.57±3.10	88.19±3.92	77.49±4.24	71.92±3.45	68.89±3.65	72.31±5.41	78.27±6.56	76.20±6.80
**Thigh (cm)**	45.74±2.21	48.18±2.16	44.07±2.22	41.22±2.33	40.00±2.20	41.96±3.02	44.56±3.45	43.66±2.53
**Chest (cm)**	101.10±4.36	107.80±5.82	90.26±3.81	91.52±5.34	82.49±3.51	85.90±5.62	98.17±8.24	93.91±9.45
**Forearm (cm)**	28.21±1.61	29.48±1.63	24.79±2.09	25.32±1.72	22.00±1.20	22.81±1.77	27.37±2.36	25.79±3.07
**MDL (lb)**	331.30±19.26	324.10±33.54	225.00±43.81	272.60±44.94	178.10±26.24	186.60±30.49	306.20±43.59	264.70±69.38
**SPT (m)**	10.91±1.47	11.88±1.88	7.59±1.94	8.82±1.76	5.82±1.07	6.11±1.22	10.31±2.02	8.85±2.68
**HRPU**	51.04±8.10	45.00±10.02	34.34±10.70	46.85±10.50	32.60±10.21	31.25±9.73	47.86±9.55	42.09±12.44
**SDC (sec)**	87.33±5.98	89.22±8.08	108.60±14.07	97.90±11.83	123.11±12.30	119.50±12.80	92.17±11.13	101.60±17.50
**LT**	17.63±3.67	13.07±5.92	7.00±5.89	16.00±5.21	6.64±6.05	5.13±4.84	15.94±4.99	12.19±7.13
**2-mile run (sec)**	854.10±83.74	916.90±123.60	962.10±122.2	880.90±128.70	957.30±113.90	963.90±15.60	880.30±110.80	911.10±119.90

[*]Body composition was collected in 47 of the participants with 40% females. The participant numbers for each cluster are: Cluster 1 (13) Cluster 2 (8), Cluster 3 (10), Cluster 4 (5), Cluster 5 (11)

Table 2: Correlations between body height, site circumference, weight, BMI and % body fat with raw scores on the six ACFT events. Significant correlations are denoted with asterisks at significance level *p < 0.05; **significant at p < 0.001; *** significant at p < 0.0001. Abbreviations are MDL – maximum deadlift, SPT – standing power throw, HRPU – hand-release push-up, SDC – spring drag carry, LT – leg tuck, BMI – body mass index.

**Measurement**	**MDL**	**SPT**	**HRPU**	**SDC**	**LT**	**2-mile Run**
Height	0.66***	0.74***	0.31***	-0.72***	0.42***	-0.35***
Bicep	0.82***	0.78***	0.56***	-0.72***	0.54***	-0.23***
Lower Bicep	0.78***	0.77***	0.510***	-0.72***	0.515***	-0.22***
Chest to Hip	0.65***	0.55***	0.604***	-0.54***	0.60***	-0.27***
Chest to Waist	0.70***	0.58***	0.67***	-0.61***	0.69***	-0.35***
Calf	0.54***	0.53***	0.34***	-0.52***	0.29***	-0.10***
Chest	0.78***	0.75***	0.51***	-0.69***	0.51***	-0.21**
Forearm	0.80***	0.79***	0.55***	-0.74***	0.56***	-0.23***
High Hip	0.32***	0.40***	-0.03	-0.33***	-0.02	0.06
Hip	0.41***	0.47***	0.06	-0.41***	0.06	0.01

Mid-thigh	0.65***	0.67***	0.31***	-0.60***	0.26***	-0.09
Neck	0.75***	0.71***	0.48***	-0.65***	0.50***	-0.28***
Lower Thigh	0.57***	0.58***	0.26***	-0.53***	0.21***	-0.11
Upper Thigh	0.58***	0.57***	0.21***	-0.52***	0.20***	-0.02
Waist Abdominal	0.55***	0.60***	0.19***	-0.49***	0.15*	-0.03
Waist to Hip	0.42***	0.44***	0.26***	-0.33***	0.19**	-0.08
Lower Waist	0.18**	0.27***	-0.14*	-0.17**	-0.18**	0.15*
Narrowest Waist	0.74***	0.75***	0.40***	-0.66***	0.38***	-0.17**
Weight	0.76***	0.81***	0.37***	-0.73***	0.38***	-0.21**
BMI	0.64***	0.63***	0.32***	-0.54***	0.25***	-0.04
% Body Fat	-0.50***	-0.50***	-0.65***	0.67***	-0.65***	0.59***

Table 3 Statistically significant terms and adjusted R^2 for regression model predicting event scores from Styku measured circumferences. Additional model details are provided in the Supplemental Materials (**Online Supplemental File 6**).

*Abbreviations are MDL – maximum deadlift, SPT – standing power throw, HRPU – hand release push-up, SDC – sprint-drag-carry, LT – leg tuck.

	**MDL**	**SPT**	**HRPU**	**SDC**	**LT**	**2-mile run**
**Adj R^2**	0.83	0.77	0.59	0.72	0.67	0.25
**Statistically significant**	Sex (p<0.001)	Sex (p=0.005)	Sex (p=0.002)	Sex (p=0.022)	Sex (p<0.001)	Height (p<0.001)

**(p<0.05) terms**	Bicep (p=0.003)	Height (0.005) Forearm (0.014)	Bicep (p<0.001) Upper Thigh (p=0.034)	Height (p<0.001) Chest (p=0.032)	Bicep (p<0.001) Forearm (p=0.024) Lower Bicep (p=0.034)	Neck (p=0.002) Lower Thigh (p=0.006) Upper Thigh (p=0.041)

Figure Legends:

Figure 1: The avatar (on right) and circumferences that are output from the Styku scan device software. Left and right circumference measurements were averaged for statistical analysis.

Figure 2: Heat map of body measurements by cluster depicting the number of standard deviations from the mean value. Red shading indicates that the value is above the mean with darker red shading corresponding to higher distance away from the mean. Similarly, green shading indicates that the value is below the mean with darker green corresponding to a higher distance away from the mean.

Figure 3: Standardized coefficients resulting from regression models that predict performance by event from Styku measurements. Positive coefficients represent terms that lead to higher scores, negative coefficients represent terms that lead to lower scores. Coefficients highest in magnitude are most influential.

Click here to access/download;Figure;Figure 1.docx

Circumferences	Body Site (L=Left, R=Right)
A	Bicep (L, R)
B	Lower Bicep (L, R)
C	Forearm (L, R)
D	Upper Thigh (L, R)
E	Mid-thigh (L, R)
F	Lower Thigh (L, R)
G	Calf (L, R)
H	Chest
I	Abdominal Waist
J	Narrowest Waist
K	Lower Waist
L	High Hip
M	Hip

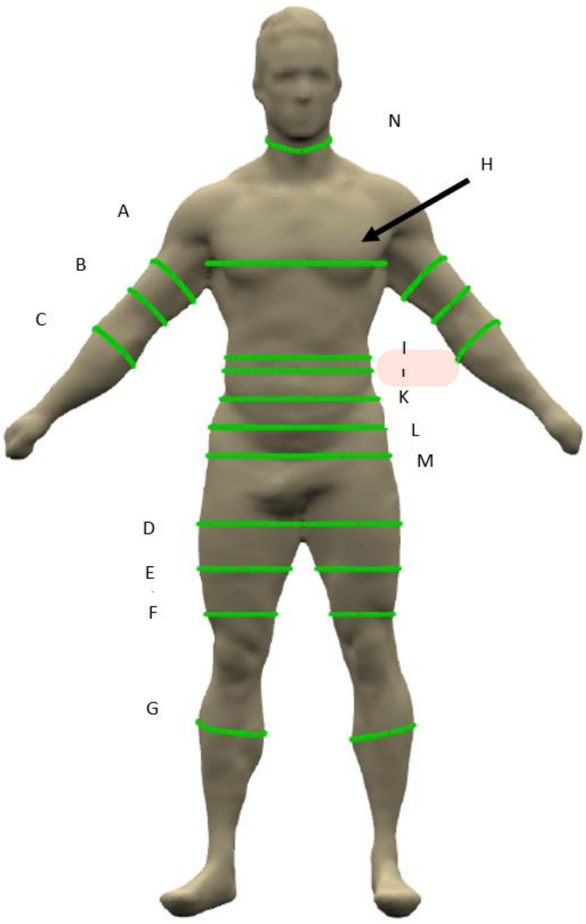

Figure

Click here to access/download;Figure;Figure 2.png

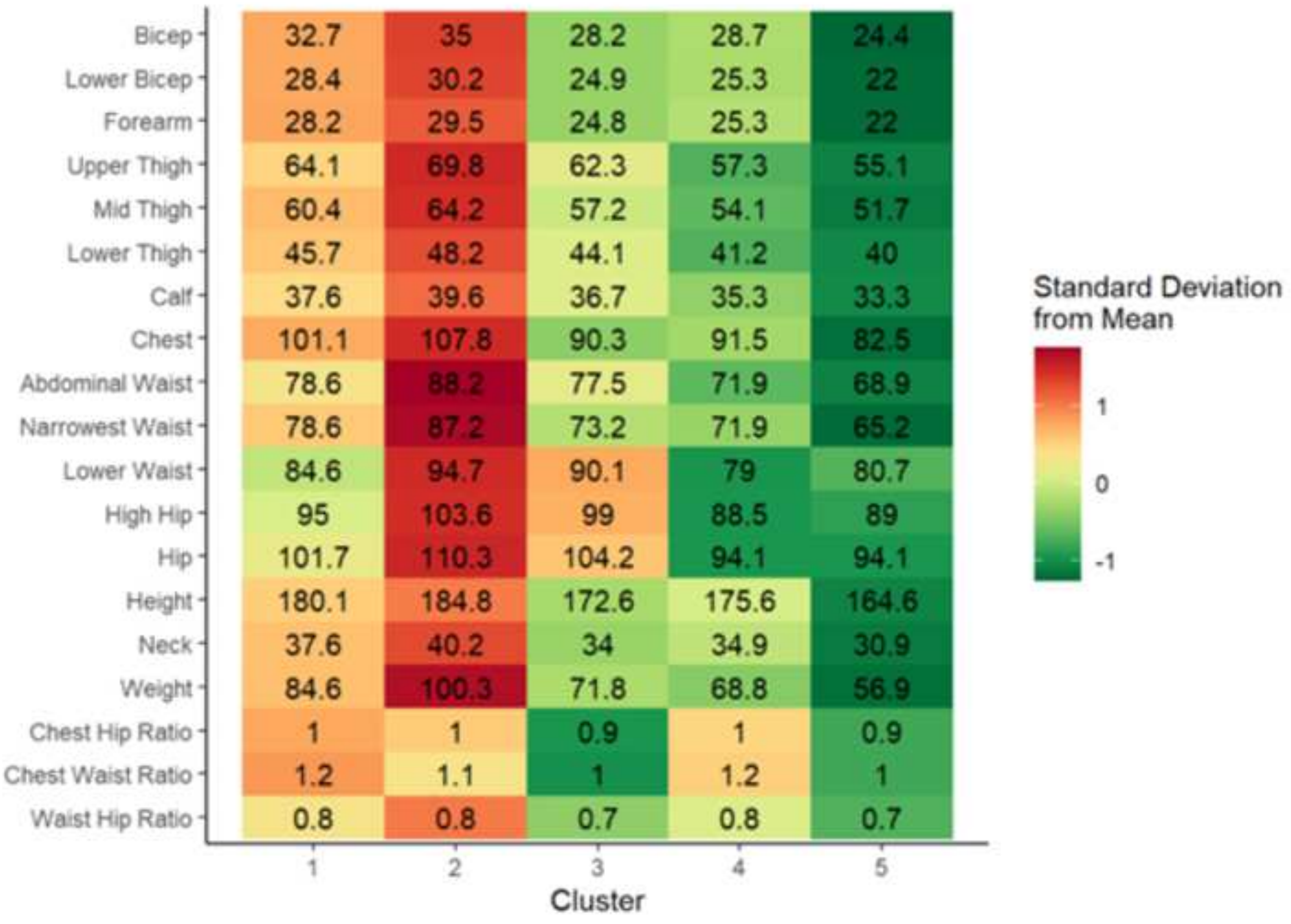

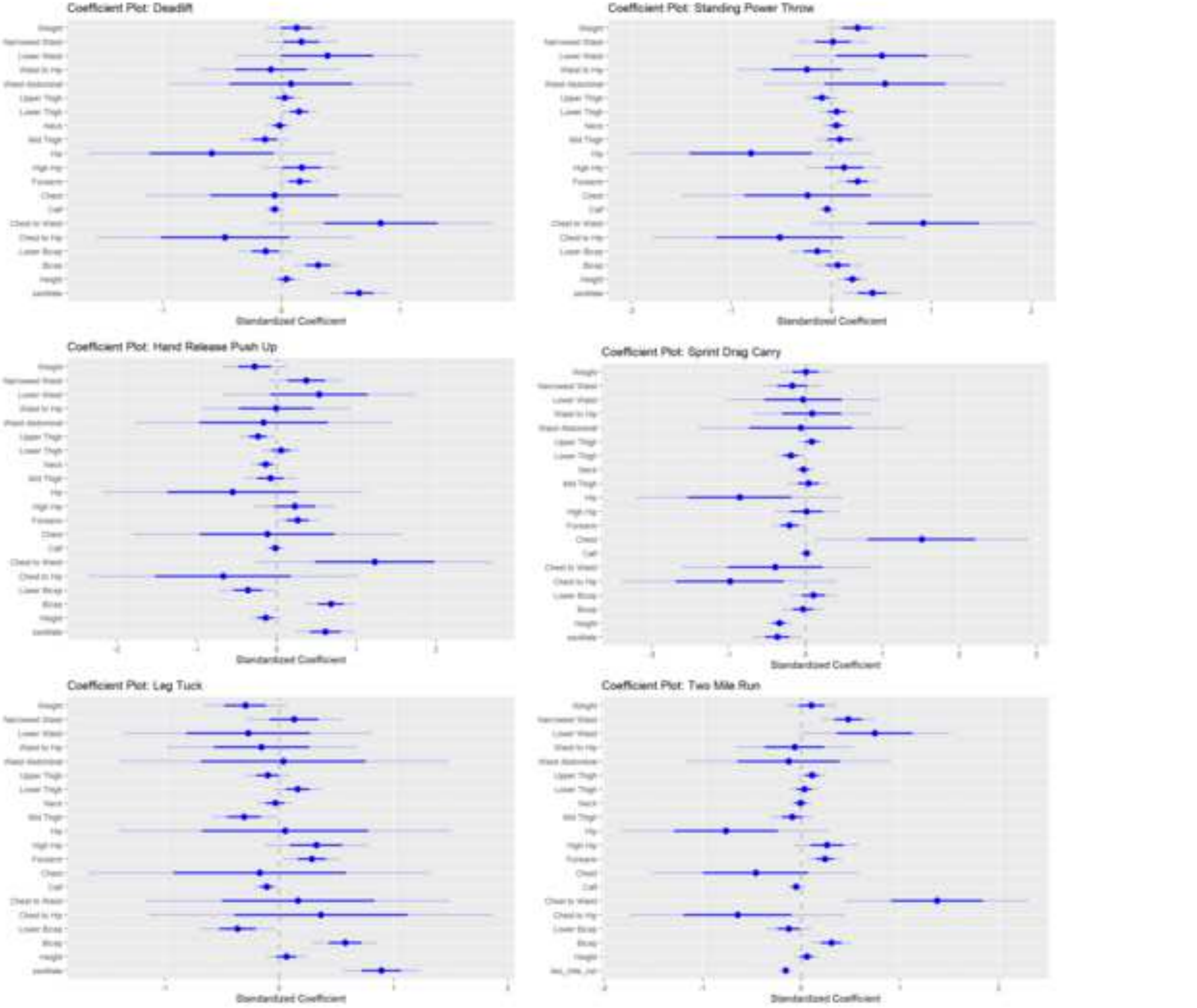
Coefficient Plot: Deadlift
Standardized Coefficient
Coefficient Plot: Standing Power Throw
Standardized Coefficient
Coefficient Plot: Hand Release Push Up
Standardized Coefficient
Coefficient Plot: Sprint Drag Carry
Standardized Coefficient
Coefficient Plot: Leg Tuck
Standardized Coefficient
Coefficient Plot: Two Mile Run
Standardized Coefficient

ACFT Description

Click here to access/download
Supporting Information
Supplemental File 1 (ACFT description).docx

Scree Plot

Click here to access/download
Supporting Information
Supplemental File 2 (scree plot).docx

Box Plots of Performance by Cluster

Click here to access/download
Supporting Information
Supplemental File 3 (box plots performance).pdf

Pairwise t-tests

Click here to access/download
Supporting Information
Supplemental File 4 (difference in means performance).pdf

Correlation Matrix Plots Performance and Shape

Click here to access/download
Supporting Information
Supplemental File 5 (correlation matrix plots).pdf

Click here to access/download
Supporting Information
Supplemental File 6 (R Markdown).pdf

Click here to access/download
Supporting Information
Supplemental File 7 (correlation matrix plots percent fat).pdf
