# Supplementary material for: Body shape and performance on the US Army Combat Fitness Test: Insights from a 3D body image scanner
Source: PLoS One. 2023 May 3;18(5):e0283566. doi: 10.1371/journal.pone.0283566 (PMC10155989; doi:10.1371/journal.pone.0283566)
Supplement: Attachment — Submitted filename: Response to Reviewers R2.docx [file pone.0283566.s010.docx]

**Response to Reviewers**

Thank you to the reviewers for the time and effort they took to improve the manuscript. We realized that a previously reviewed section that had no comments was accidently omitted in Revision 2 when we rearranged the flow of the manuscript. In addition a section that appeared in the response to reviewers on regression models with cluster as a independent variable was omitted from the manuscript We have placed these sections back at the end of the results (highlighted) and paste it below here for the Reviewer’s convenience.

*ACFT performance by cluster*

Overall average performance on every ACFT event was highest in Cluster 1 on every event except the SPT (**Table 1, Box Plots Online Supplemental File 3**). Participants classified to Cluster 2 had the highest performance on the SPT event and second highest performance on the MDL and SDC events. Individuals in this cluster had similar performance to Cluster 4 on the HRPU event and performed below Cluster 4 on the LT and the 2-mile run. Participants in Cluster 3 performed above Cluster 5 on all events except the 2-mile run.

Comparison of means between clusters revealed that Clusters 1 and 2 had statistically significant better performance than Clusters 3, 4 and 5 for the MDL (Online **Supplemental File 5**). Additionally, Clusters 3 and 4 had significantly better performance on the MDL compared to Cluster 5. For SPT, HRPU and SDC and LT, Clusters 1 and 2 had statistically significant better performance than Clusters 3 and 5. Finally, Cluster 4 had statistically significant better performance on the SDC and LT compared to Cluster 5. There were no statistically significant differences in performance over any ACFT event between Cluster 3 and Cluster 4.

*Regression Models with Cluster Number as a covariate*

Similar adjusted R^2^ were found for the six models predicting performance on each ACFT event compared to the original multiple linear regression models that were developed without cluster number as a covariate. The full statistical output can be found in Online **Supplemental File 6** as a R Markdown file.

We had only two comments to address which we address below:

Reviewer #1

**Comment 1:** Dear Authors the revised version of the manuscript appear improved.
However, in the reviewer's opinion, the body composition part is not fully within the scope of the study and given the lack of data it is suggested that the DXA analysis part (also from the title) be deleted. This would make the manuscript more organized and linear, with a clearer purpose of the study. Given the sample size, it seems to be a solution that allows for better understanding of the text.

**Response:** We have removed all references to body composition, percent fat and DXA in the manuscript. We have eliminated body composition from the title. These changes are not visible in the marked up manuscript, because you cannot highlight a section you have deleted, but we hope the reviewer can see the change easily.

**Comment 2:** The purpose of the study is not clear at the end of the introduction section.

**Response:** We agree and we have changed this last paragraph in the introduction to reflect the purpose of the study. The paragraph is pasted below for the reviewer’s convenience.

**We wanted to understand the role body shape on ACFT performance. Specifically, what are the archetype body shapes that predispose individuals to better ACFT performance and alternatively which archetype body shapes that are associated to poorer ACFT performance. Addressing these questions can offer opportunities for training programs to target specific body sites associated with better ACFT performance.**
